# Antihypertensive treatment guided by genetics: PEARL-HT, the randomized proof-of-concept trial comparing rostafuroxin with losartan

**DOI:** 10.1038/s41397-021-00214-y

**Published:** 2021-03-01

**Authors:** Lorena Citterio, Giuseppe Bianchi, Giuseppe A. Scioli, Nicola Glorioso, Roberto Bigazzi, Daniele Cusi, Jan A. Staessen, Silvio Cavuto, Mara Ferrandi, Chiara Lanzani, Xiaoyi Li, Lit-Fui Lau, Chern-En Chiang, Tzung-Dau Wang, Kang-Ling Wang, Patrizia Ferrari, Paolo Manunta

**Affiliations:** 1 Genomics of Renal Diseases and Hypertension Unit, IRCCS San Raffaele Scientific Institute, Università Vita Salute San Raffaele, Milano, Italy; 2 Università Vita Salute San Raffaele, Milan, Italy; 3 Hypertension and Cardiovascular Prevention Center, Ospedale Ferdinando Veneziale, Isernia, Italy; 4 Hypertension and Related Diseases Center, Department of Clinical and Experimental Medicine, University of Sassari, Sassari, Italy; 5 Nephrology and Dialysis Unit, Livorno, Italy; 6 Institute of Biomedical Technologies Milano National Research Council of Italy (CNR), Segrate, Milano, Italy; 7 Bio4Dreams Scientific Unit, Bio4Dreams—Business Nursery for Life Sciences, Milano, Italy; 8 Research Unit Hypertension and Cardiovascular Epidemiology KU Leuven Department of Cardiovascular Sciences, University of Leuven, Leuven, Belgium; 9 Clinical Trials and Statistics Unit, Azienda USL—IRCCS di Reggio Emilia, Reggio Emilia, Italy; 10 Windtree Therapeutics, Warrington, PA USA; 11 CVie Therapeutics, Taipei, Taiwan; 12 Zhaoke (Guangzhou) Ophthalmology Pharmaceutical Limited, Guangzhou, China; 13 General Clinical Research Center, Taipei Veterans General Hospital and National Yang-Ming University, Taipei, Taiwan; 14 Cardiovascular Center and Division of Cardiology, Department of Internal Medicine, National Taiwan University Hospital and College of Medicine, National Taiwan University, Taipei, Taiwan

**Keywords:** Predictive markers, Cardiovascular diseases, Genetic association study, Disease genetics

## Abstract

We compared a standard antihypertensive losartan treatment with a pharmacogenomics-guided rostafuroxin treatment in never-treated Caucasian and Chinese patients with primary hypertension. Rostafuroxin is a digitoxigenin derivative that selectively disrupts the binding to the cSrc-SH2 domain of mutant α-adducin and of the ouabain-activated Na-K pump at 10^–11^ M. Of 902 patients screened, 172 were enrolled in Italy and 107 in Taiwan. After stratification for country and genetic background, patients were randomized to rostafuroxin or losartan, being the difference in the fall in office systolic blood pressure (OSBP) after 2-month treatment the primary endpoint. Three pharmacogenomic profiles (P) were examined, considering: P1, adding to the gene variants included in the subsequent P2, the variants detected by post-hoc analysis of a previous trial; P2, variants of genes encoding enzymes for endogenous ouabain (EO) synthesis (*LSS* and *HSD3B1*), EO transport (*MDR1/ABCB1*), adducin (*ADD1* and *ADD3*); P3, variants of the *LSS* gene only. In Caucasians, the group differences (rostafuroxin 50 μg minus losartan 50 mg in OSBP mmHg) were significant both in P2 adjusted for genetic heterogeneity (P2a) and P3 *LSS* rs2254524 AA [9.8 (0.6–19.0), *P* = 0.038 and 13.4 (25.4–2.5), *P* = 0.031, respectively]. In human H295R cells transfected with *LSS* A and *LSS* C variants, the EO production was greater in the former (*P* = 0.038); this difference was abolished by rostafuroxin at 10^–11^ M. Chinese patients had a similar drop in OSBP to Caucasians with losartan but no change in OSBP with rostafuroxin. These results show that genetics may guide drug treatment for primary hypertension in Caucasians.

## Introduction

Rostafuroxin has been developed as a selective inhibitor of the ouabain blood pressure effects initially [1–3]. Later rostafuroxin has also been found to inhibit the effects of mutant adducin [4, 5] that was previously shown to be associated with hypertension in the Milan Hypertensive Rat Strain [6, 7] and in humans [8–11]. Rostafuroxin exerts its dual mechanism by selectively disrupting the binding to the cSrc-SH2 domain of mutant α-adducin and of the ouabain-activated Na-K pump at 10^–11^ M, being inactive on the effects of wild adducin or of other 35 proteins or receptors involved in blood pressure regulation up to 10^–5^ M [12, 13]. Therefore, rostafuroxin may be used as a safe, small molecule molecular probe [14, 15] to disentangle the genetic complexity of primary hypertension in humans and rats, facilitating and strengthening the comparisons between the two species. Rostafuroxin may display its therapeutic benefits by reducing the magnitude of cSrc triggered signal transduction, which favors renal tubular sodium reabsorption [9, 10, 15–17], hypertension, and cardiac/renal damage [2, 5, 18–20] in carriers of gene variants affecting adducin function and/or ouabain tissue concentrations.

Four issues should be properly addressed when applying genetic tools to improve the beneficial effects of therapy of primary hypertension in clinical practice: (1) genetic heterogeneity, where different genes or different alleles within the same gene, may be involved in the regulation of the same phenotype [15, 21]; (2) epistasis, where many gene modifiers may either enhance or inhibit the effect of a given gene [22, 23]. Since the influence of modifiers differ among species, this hampers any kind of straightforward animal model-human comparison needed to assess causation; (3) moving from association to causation, relatively straightforward move in rodents with DNA manipulations [24], harder in humans, where a selective drug, that inhibits a specific genetically mediated pathway is needed; (4) avoiding the influence of the phases of hypertension and of the confounding effects of previous therapy that may take over, magnify or blunt the initial triggering mechanisms as shown in two well known causes of hypertension [25, 26]. After having obtained data supporting a plausible role of adducin variants or of endogenous ouabain (EO) in triggering hypertension in a rodent model and in humans [1, 2, 6–11], we developed a strategy that takes into account the above mentioned four issues by, (1) applying the concept of genetic profile, in order to capture at least a portion of the genetic heterogeneity underlying adducin and EO functions [13]; (2) limiting the studies to newly discovered and never treated (naïve) patients [13]; (3) assessing causation with rostafuroxin [4, 12]. By applying this strategy, we identified the genetic profile 2 (P2), associated with the rostafuroxin blood pressure response, within the phase 2a OASIS-HT trial (ouabain and adducin for specific intervention on sodium in hypertension trial) [13]. P2 consists of variants at α-adducin (*ADD1* rs4961), γ-adducin (*ADD3* rs3731566), lanosterol synthase (*LSS* rs2254524), 3β-hydroxysteroid dehydrogenase/δ(5)-δ(4)isomerase type 1 (*HSD3B1* rs10923835), and ATP-binding cassette subfamily B member 1 (*ABCB1/MDR1* rs1045642) genes, the last three being involved in the regulation of EO tissue levels.

We undertook the present phase 2b, double-blind, randomized, controlled PEARL-HT trial (PharmacogEnetic Assessment of Rostafuroxin vs Losartan in HyperTension) for validating the previous findings [13]. To this aim, we compared the office systolic blood pressure (OSBP) response to rostafuroxin and losartan in three subsets of naïve hypertensives, who differed for the gene variant number and the validity of previous existing data supporting their involvement. These three subsets included patients with at least (i) one combination of the above listed P2 gene variants, plus other variants that have been detected by a post-hoc analysis on the previous OASIS-HT trial, named Profile 1 (P1); (ii) one gene combination of the P2 listed above; (iii) homozygous wild CC (major) and homozygous for the mutant genotype AA (minor) of *LSS* rs2254524 (P3). To confirm the role of EO in our hypothesis, we transfected EO producing human adrenocortical cells (H295R) with *LSS* A or *LSS* C variants, to test if the previously shown increased EO production [13] in the former was inhibited by rostafuroxin at 10^–11^ M.

## Materials and methods

### Study design, characteristics of the enrolled patients

This PEARL-HT clinical trial (EudraCT, identifier 2010-022073-34 and ClinicalTrials.gov, identifiers NCT01320397) phase 2b multicenter, double-blind, double-dummy, four-arms, parallel group, and active comparator-controlled study was conducted in newly discovered and never-treated (naïve) hypertensive patients enrolled in 13 Italian and in 15 Taiwanese centers (Table S1). Besides the fact that we aimed to compare a generic drug as losartan that is one of the mainly used in the treatment for hypertension, the rationale underlying the choice of naïve patients and the losartan as a comparator is explained in the online-only Supplementary Data.

**Fig. 1:**
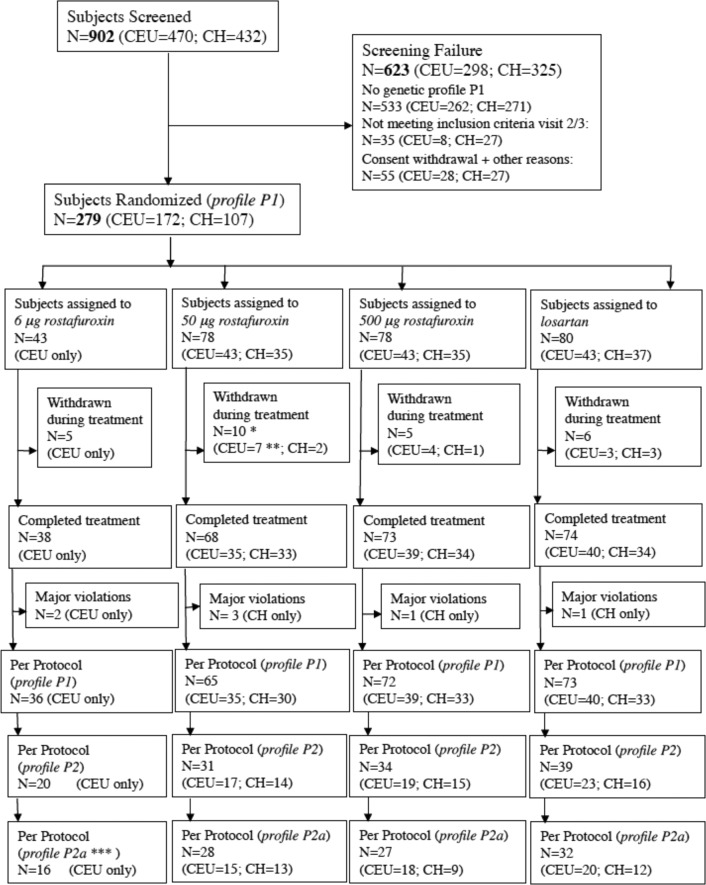
CONSORT patients flow for PEARL-HT clinical trial. *1 patient withdrawn without any treatment. **in the 50 μg treatment group, the DO of four patients was requested by the sponsor because of a non-compliance problem in the initial rostafuroxin 50 μg capsules. ***to comply with the pre-specified definition of the PP population to evaluate the primary end point, the patients randomized as the carrier of a gene pair alone or in combination, which are not present in all the four arms, have to be removed from the profile P2 to obtain the profile P2a. Therefore, four, two, one, and three patients were removed from the 6, 50, and 500 μg rostafuroxin and losartan arms, respectively. CONSORT, Consolidated Standards of Reporting Trials.

Two oral daily doses of rostafuroxin (50 and 500 μg) have been studied vs 50 mg losartan both in Caucasian and Chinese patients (Fig. 1), who received both one capsule containing one of the two doses of rostafuroxin or placebo of rostafuroxin and one tablet containing 50 mg losartan or placebo of losartan. The treatment period lasted 9 weeks. An additional arm of 6 μg rostafuroxin was carried out in Caucasian patients only. The chemical and pharmacologic characteristics of rostafuroxin are described elsewhere [4, 5].

The eligibility of the naïve hypertensive patients included: age between 25 and 60 years, being carrier of one or a combination of polymorphisms of the P1 (Table 1 and Table S2), with at least 50% of the enrolled patients having a combination of gene variants of the original P2 (OASIS-HT) [13], to have already undertaken lifestyle recommendations and still having sitting OSBP ranging between 140 and 169 mmHg and Office Diastolic BP (ODBP) ranging between 85 and 100 mmHg. Though eligible patients should not have been previously treated with any specific antihypertensive drug, a short period of treatment (no longer than seven days) was allowed before enrolment if a washout of about 1 month could be applied before randomization. Other criteria for non-eligibility were: statin treatment, presence of renal or adrenal secondary forms of hypertension, fasting plasma glucose >125 mg/dl (6.9 mmol/L), estimated creatinine clearance ≤50 ml/min, and if women, being pregnant, nursing, or of childbearing potential not taking anti-contraceptive medication. Local ethics committees approved the research protocol (Text S1).

**Table 1:** Overall profile 1 (P1) in Caucasian and Chinese.

Caucasian	Chinese
***ADD1*** **rs4961 GT** **+** **TT** ***& ADD3*** **rs3731566 GG**	***ADD1*** **rs4961** **GT** + **TT** *&* ***ADD3 rs2501574 TT***
***ABCB1/MDR1*** **rs1045642 TT** ***& HSD3B1*** **rs10923835 AT** **+** **TT**	***ABCB1/MDR1*** **rs1045642 TT** ***&*** ***HSD3B1 rs117585927 CG + CC***
***LSS*rs2254524 CA** **+** **AA** ***& ABCB1/MDR1*** **rs1045642 CC**	***LSS*rs2254524 CA** + **AA** ***& ABCB1/MDR1*** **rs1045642 CC**
***LSS*rs2254524 AA** ***& ADD1*** **rs4961 GT** **+** **TT**	***LSS rs2254524 AA & ADD1*** **rs4961 GT** + **TT**
rs16893522 = AA	rs16893522 = AA
rs2345088 = TT	rs2345088 = TT
rs2461911 = AA	rs2461911 = AA
rs16877182 = CT	rs16877182 = CT
rs12513375 = GG	rs12513375 = GG
*HSD3B1* rs947130 GG & *NEDD4L* rs4245268 CC	*HSD3B1* rs947130 GG & *NEDD4L* rs4245268 CC
*ABCB1/MDR1* rs1045642 CC & *AGTR1* rs2131127 CC	*ABCB1/MDR1* rs1045642 CC & *AGTR1* rs2131127 CC
*ADD2* rs4984 CC & rs10502933 CT	*ADD2 rs12470211 AG + GG* & rs10502933 CT
*LSS* rs2254524 AA & *WNK1* rs880054 AG + GG	*LSS* rs2254524 AA & *WNK1* rs880054 AG + GG

Genotype combinations in P1 and P2 (in bold) both in Caucasian and in Chinese. Genotype variations specific to Chinese are in italics and underlined, as explained in the online-only Supplementary Data. P1 was composed by adding to the gene variants included in the P2, the variants detected by post-hoc analysis of a previous OASIS-HT trial; P2, variants of genes encoding enzymes for endogenous ouabain (EO) synthesis (*LSS* and *HSD3B1*), EO transport (*MDR1/ABCB1*), adducin (*ADD1* and *ADD3*); P3, variants of the *LSS* gene only (homozygous wild CC (major) and homozygous for the mutant genotype AA (minor)).

### Central genotyping and randomization

Genomic DNA was extracted from peripheral blood samples collected at screening visit (visit 1) and genotyped using TaqMan OpenArray Genotyping System (Life Technologies, Foster City, CA) at San Raffaele Scientific Institute (Milan, Italy) according to the manufacturer’s instructions. See more details in the online-only Supplementary Data.

Assignment to treatment groups was determined by a computer-generated random sequence using an interactive web-based response system as described in the online-only Supplementary Data.

### Selection of the eligible patients and study procedures

The eligible patients were identified either by a Clinic Center Physician or a General Practitioner who prescribed life style changes. Then, these patients were followed by the physician in charge of this study at the hospital center, who carried out the following visits: at visit 1 screening: collection signed Informed Consent, check prior and concomitant medications, blood sampling for chemistry, pregnancy test, genotyping, ECG, providing recipient for 24-hour urine collection (to measure sodium excretion), OSBP, and ODBP. Check of verifiable inclusion/exclusion criteria (see Protocol in Text S1). Visit 1 was followed by other two run in visits 2 and 3, and post-randomization visits 4, 5, 6, and 7 at 2, 4, and 6 weeks, respectively. Visit 7 was carried out one day after visit 6, just to remove the 24-hour Ambulatory Blood Pressure (24h-ABPM) device. The baseline 24h-ABPM was carried out between visit 2 and visit 3. At visit 3 final inclusion/exclusion criteria, randomization and dispensing the drug boxes for the subsequent drug period; at visits 3, 4, 5, and 6 office BP and HR measurements as at visits 1 and 2, check of concomitant disease, medications, adverse events, and drug accountability; measurements, blood sampling for chemistry, and pregnancy test.

### Definition of confounders, distinction between primary and secondary variables and statistical analysis

The rationale for choosing the genetic background and the basal BP as confounders with the distinction between primary and secondary variables is described in the online-only Supplementary Data.

The average of the last three readings of OSBP were considered in the statistical analysis. All BP variables were assumed to be normally distributed. Standing the definition of the primary objective in the protocol, as the OSBP difference between visit 6 and visit 3, the primary efficacy analyses were carried out in the Per Protocol (PP) population, while the safety analyses were carried-out in the safety population. The one-way analysis of variance (ANOVA) was tested for the first step of analysis, with the corresponding mean values with SD. The comparison between treatment groups has been carried out with an analysis of covariance (ANCOVA) model with change from baseline to visit 6 as dependent variable, treatment, country, and baseline as covariate and the data presented as mean with standard error (SE) and 95% CI. The Levene’s test confirmed the homoscedasticity of variance of OSBP between the groups of comparison.

### Outcomes

The relatively long treatment duration of 2 months, implying the use of a PP population for primary analysis instead of an Intention to Treat (ITT) population, is based on the similarities between the rostafuroxin and spironolactone action mechanisms and pharmacodynamics. Both drugs counteract the excess renal tubular sodium reabsorption responsible for BP increase in carriers of the P2 or adrenal adenoma, respectively. Indeed, a treatment of several weeks is needed to fully exploit the spironolactone antihypertensive activity [27].

The two primary objectives were to demonstrate that at least one of the two highest doses of rostafuroxin (50 or 500 μg) reduces significantly OSBP at visit 6 compared to 50 mg losartan, in patients bearing either:

(1) at least one pair of gene variants of the P2, or

(2) at least one variant or pair of variants included in the P1.

Because the considerable variation among the frequencies of the four gene-pairs of the P2 detected before unblinding, in the statistical analysis plan (SAP) (Text S2) and in the data review report (DRR) (Text S3) it was stated: “the need to minimize the genetic heterogeneity across the treatment arms by maximizing the frequency similarity of the gene-pairs across the different arms”.

Thus, the results of the treatment efficacy within the four arms are given both as a P2 and P2a, representing, respectively, the unadjusted and the adjusted results for the genetic heterogeneity, being the latter considered the one to use to assess the primary end-point, as pre-specified in the SAP. An additional analysis, also pre-specified in SAP, has been performed considering *LSS* rs2254524 polymorphism (genotypes AA and CC) alone to confirm previous data [13].

### *LSS* expression analysis and endogenous ouabain quantification in H295R cells

Transfection with *LSS* major (C-642Val) and minor (A-642Leu) variants was investigated in human adrenocortical cells (H295R cells), as described [13]. EO was determined by radioimmunoassay on C18–extracted samples using a specific antiserum [3]. More details of these methods are given in the online-only Supplementary Data.

### Study power and cell data statistical analysis

According to the assumptions in the protocol (Text S1) and SAP (Text S2), a total sample size of 120 patients (50% of the total sample), i.e., 40 patients in each group, is suitable for discriminating an OSBP mean difference of 6.5 mmHg between the groups of the two highest doses of rostafuroxin and losartan, with a standard deviation of 10 mmHg, a power of 80%, an alpha level of 0.05, two tailed test, in the PP and ITT genetic P2 populations, and assuming a 10% drop-out rate. However, as shown in Table 2 discussed in “Results”, the lack of OSBP response to rostafuroxin in Chinese patients required an analysis by country, thus the above sample size was reduced by 50%.

**Table 2:** OSBP baseline values and changes (mmHg) after two months based on treatment in carriers of genetic profiles P1 and P2 for Caucasian (IT) and Chinese (TW).

		6 μg rostafuroxin		50 μg rostafuroxin		500 μg rostafuroxin		losartan	
OSBP		OSBP baseline (mmHg)	OSBP change after 2 months (mmHg)	OSBP baseline (mmHg)	OSBP change after 2 months (mmHg)	OSBP baseline (mmHg)	OSBP change after 2 months (mmHg)	OSBP baseline (mmHg)	OSBP change after 2 months (mmHg)
		**P1**		P1		**P1**		P1	
**IT**	**Mean**	149.6	−5.8*	151.2	−17.3**	150.4	−16.0**	151.4	−17.5**
***n*** = **150**	**SD**	6.1	14.4	6.2	16.2	5.9	14.4	7.5	14.9
		***n*** = **36**		***n*** = **35**		***n*** = **39**		***n*** = **40**	
**TW**	**Mean**	*–*	*–*	146.5	−2.7^§^	146.2	−4.6^§§^	146.6	−15.9^§§^
***n*** = **96**	**SD**	*–*	*–*	6.5	11.1	7.2	9.9	7.1	11.7
				***n*** = **30**		***n*** = **33**		***n*** = **33**	
		**P2**		P2		**P2**		P2	
**IT**	**Mean**	151.9	−8.7^#^	151.4	−22.2^##^	151.7	−19.7^##^	150.5	−13.1^##^
***n*** = **79**	**SD**	6.6	14.8	7.3	16.6	6.5	15.1	8.4	13.2
		***n*** = **20**		***n*** = **17**		***n*** = **19**		***n*** = **23**	
**TW**	**Mean**	*–*	*–*	144.7	−0.2^@^	145.9	−4.7^@@^	146.7	−15.4^@@@^
***n*** = **45**	**SD**	*–*	*–*	4.6	6.5	7.4	11.5	8.1	13.4
				***n*** = **14**		***n*** = **15**		***n*** = **16**	

Data from ANOVA are means ± SD. P1 IT, *P = 0.021; **P < 0.001. P2 IT, ^#^*P* = 0.010; ^##^*P* < 0.001. P1 TW, ^§^*P* = 0.174; ^§§^*P* = 0.018; ^§§§^*P* < 0.001. *P*2 TW, ^@^*P* > 0.5; ^@@^*P* = 0.108; ^@@@^*P* < 0.001.

–, not determined.

For H295R cell culture transfection, data were expressed as means ± SE with 95% CI. Statistical comparisons were analyzed by Student’s t test or one-way ANOVA. When the individual measurements were below 5, the single values and means were shown. A *P* value of 0.05 was chosen for statistical significance.

## Results

Globally, 902 hypertensives were screened (Fig. 1). Of them, 470 were screened and 172 randomized in Italy between June 26, 2013 and January 8, 2016, while 432 were screened and 107 randomized in Taiwan between December 18, 2015 and December 6, 2017. Only patients carrying at least one or a pair of gene variants included in the genetic profile P1 were randomized (Table 1), with at least 50% of them having the combination of the gene variants of the original P2 (OASIS-HT) [13]. The clinical characteristics of the Caucasians are given in Table S3, while those of Chinese are in Table S4. The percentage of male was about twice that of female in both populations (70% vs 30% in Caucasians, 64% vs 36% in Chinese). No mean age difference was shown between genders in both the Caucasians and the Chinese (47.8 (±7.4) males vs 50.8 (±5.9) females, and 45.9 (±8.6) males vs 48.9 (±6.4) females, respectively). As explained in “Outcome” and shown in the flowchart (Fig. 1), the P2 and P2a represented the unadjusted and the adjusted results for the genetic heterogeneity among study arms, respectively, being the latter considered the one to be used to assess the primary end point, as pre-specified in the SAP (Text S2).

### Chinese patients do not respond to rostafuroxin but to losartan

OSBP did not change in Chinese carrying either the P1 or P2 at any tested dose of rostafuroxin (Table 2). The lack of response in Chinese (−0.2±6.5 mmHg with 50 μg rostafuroxin) implies an inhibition of the “placebo” response to rostafuroxin treatment, as previously observed in Caucasians carrying the P2 [13]. Conversely, the efficacy of losartan was similar in carriers of the P2 of both ethnic groups. Therefore, only the data on Caucasian are presented and discussed hereafter.

### Caucasian carriers of the P2 and P2a, but not of the P1, have a greater fall of OSBP to rostafuroxin than to losartan

**Fig. 2:**
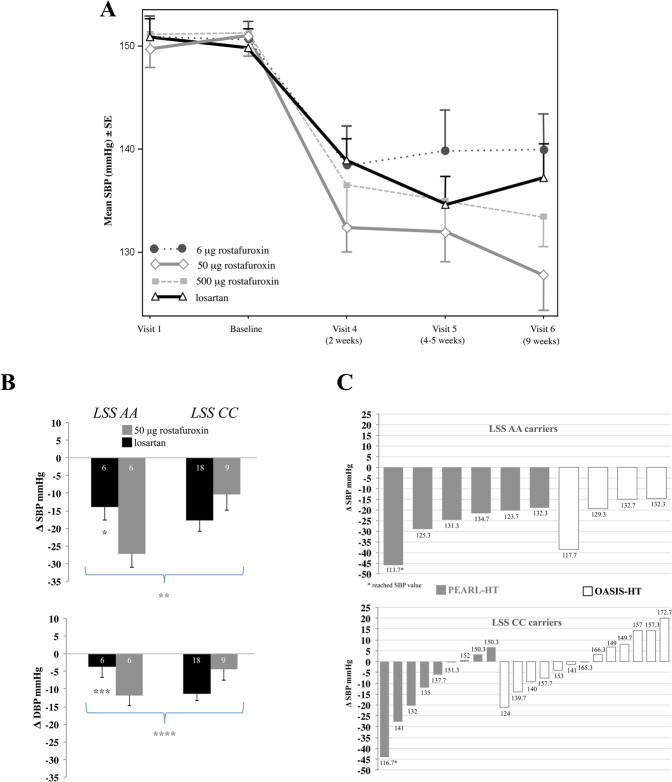
Blood pressure changes after rostafuroxin or losartan treatment in carriers of the profile P2a or of *LSS* AA or CC genotypes. **A** Time-course of mean OSBP changes (mmHg) at visit 1, at baseline, and visits 4 (2 weeks), 5 (4–5 weeks), and 6 (9 weeks) in profile P2a in the four treatments groups. Data are means ± SE. **B** OSBP and ODBP fall (mmHg) in *LSS* AA and CC carriers treated with 50 mg losartan or 50 µg rostafuroxin. ANCOVA test. Data adjusted for baseline are means (±SE). ∆ = delta OSBP or ODBP. Numbers in the columns refer to each subgroup sample size. * ∆ = 13.4 mmHg OSBP for *LSS* AA, 50 mg losartan vs 50 µg rostafuroxin, *P* = 0.031; ** *LSS* x treatment (OSBP), *P* = 0.023; *** ∆=9.1 mmHg ODBP for *LSS* AA, 50 mg losartan vs 50 µg rostafuroxin, *P* = 0.095; **** *LSS* x treatment (ODBP), *P* = 0.013. **C** OSBP fall (mmHg) in *LSS* AA and CC individual carriers treated with 50 µg rostafuroxin, in PEARL-HT and OASIS-HT trials. The values below the histograms are the OSBP levels reached by the individual patients at the end of the treatment period.

In Caucasian carrying the P1, both all rostafuroxin doses and losartan produced a statistically significant OSBP fall (*P* = 0.021 for rostafuroxin 6 μg, *P* < 0.001 for the other rostafuroxin doses and losartan; Table 2, ANOVA test). The fall of OSBP to losartan and to each of the highest doses of rostafuroxin were very similar (*P* > 0.5; ANOVA test), (Table 2). In P2a Caucasians, the OSBP decrease was statistically significant at any tested dose of rostafuroxin (Table 3 and Table S5) being greater with 50 μg rostafuroxin than with 500 μg rostafuroxin, even though this difference did not reach statistical significance (Table 3 and Table S5). This trend towards a bell-shaped curve of the rostafuroxin dose–effect relationship, with the peak at 50 μg, is consistent with previously published preclinical and clinical data of the OASIS-HT study [13]. Compared to losartan, the OSBP change was greater with the two highest doses of rostafuroxin, being this difference larger and statistically significant with the dose of 50 μg: −9.8 mmHg (−19.0; −0.6, [95% CI]) *P* = 0.038 (Table 3 and Table S5). Fig. 2a shows the OSBP values, in carriers of the P2a, at the different time points. The OSBP changes between visit 6 (at 9 weeks of treatment) and visit 5 (at 5 weeks of treatment) was +2.6 ± 1.7 mmHg and −4.2 ± 4.3 mmHg (mean ± SE) (*P* = 0.113) for losartan and rostafuroxin at 50 μg, respectively.

**Table 3:** Baseline values and the changes in OSBP from baseline to two months (mmHg), in the four arms of patients carrying the profile P2 (unadjusted for genetic heterogeneity) and P2a (adjusted for genetic heterogeneity), treated with 50 mg losartan and 6, 50, and 500 μg rostafuroxin. ANCOVA test.

	Genotypes					P2		P2a	
Treatment	*n*	P2	*n*	P2a	OSBP (mmHg)	Baseline	Change after 2 months	Baseline	Change after 2 months
**6** μ**g rostafuroxin**	4	*ADD1/ADD3*	4	*ADD1/ADD3*	***mean***	**151.9**	**−8.3**	**150.7**	**−10.8**
	4	*MDR1/HSD3B1*	4	*MDR1/HSD3B1*	***SD***	6.6		6.7	
	8	*MDR1/LSS*	8	*MDR1/LSS*	***95% CI***		−14.56, −2.10		−17.48, −4.01
	3	*ADD1/LSS*							
	1	*MDR1/LSS-ADD1/LSS*							
	total 20		total 16						
**50** μ**g rostafuroxin**	2	*ADD1/ADD3*	2	*ADD1/ADD3*	***mean***	**151.4**	**−22.2**^**#**^	**151.0**	−23.0*
	3	*MDR1/HSD3B1*	3	*MDR1/HSD3B1*	***SD***	7.3		7.7	
	10	*MDR1/LSS*	10	*MDR1/LSS*	***95% CI***		−28.92, −15.42		−29.91, −16.0
	–	*ADD1/LSS*							
	1	*ADD1/ADD3-MDR1/LSS*							
	1	*MDR1/LSS-ADD1/LSS*							
	total 17		total 15						
**500** μ**g rostafuroxin**	1	*ADD1/ADD3*	1	*ADD1/ADD3*	***mean***	**151.7**	**−19.5**^**##**^	**151.3**	−17.4**
	5	*MDR1/HSD3B1*	5	*MDR1/HSD3B1*	***SD***	6.5		6.5	
	12	*MDR1/LSS*	12	*MDR1/LSS*	***95% CI***		−25.88, −13.11		−23.74, −11.03
	1	*ADD1/LSS*							
	total 19		total 18						
**losartan**	3	*ADD1/ADD3*	3	*ADD1/ADD3*	***mean***	**150.5**	**−13.7**	**149.8**	**−13.2**
	4	*MDR1/HSD3B1*	4	*MDR1/HSD3B1*	***SD***	8.4		8.1	
	13	*MDR1/LSS*	13	*MDR1/LSS*	***95% CI***		−19.51, −7.88		−19.22, −7.15
	1	*ADD1/LSS*							
	1	*ADD1/ADD3-MDR1/HSD3B1*							
	1	*ADD1/ADD3-MDR1/LSS-ADD1/LSS*							
	total 23		total 20						

For OSBP changes data are adjusted for baseline (means and 95% CI). ^#^P2, losartan vs 50 μg rostafuroxin, *P* = 0.062; ^##^P2, losartan vs 500 μg rostafuroxin *P* = 0.185; *P2a, losartan vs 50 μg rostafuroxin, *P* = 0.038; **P2a, losartan vs 500 μg rostafuroxin *P* = 0.342.

Data are means ± SD for baseline.

Table 3 also shows the OSBP changes in P2 carries, not adjusted for the genetic heterogeneity. The magnitude of the difference between rostafuroxin and losartan was slightly lower than that of P2a (−22.2 vs −23 mmHg), as the significance (*P* = 0.062 vs *P* = 0.038).

### The *LSS* rs2254524 AA and CC genotypes differently affect the pressor response to rostafuroxin and to losartan

**Fig. 3:**
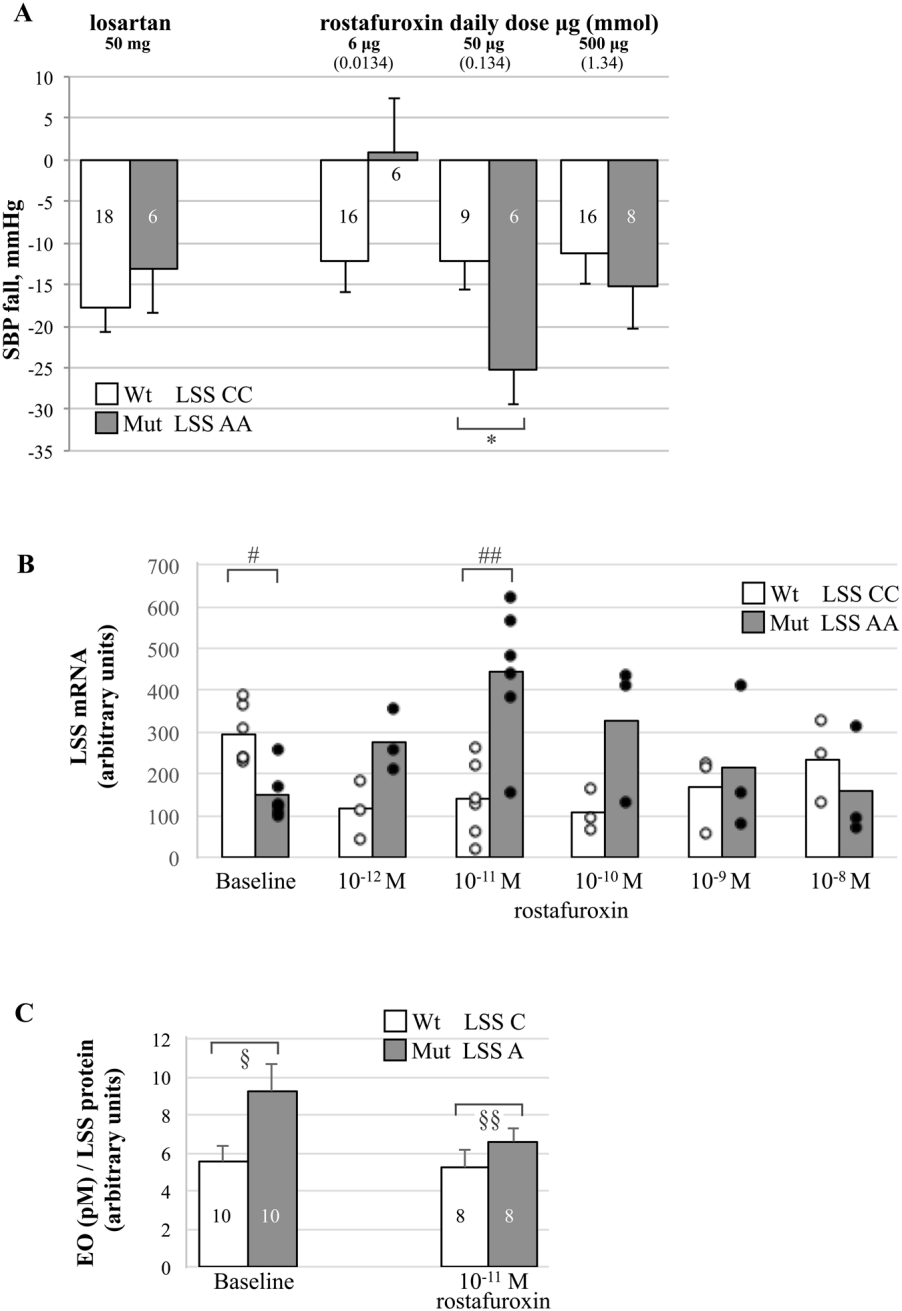
Rostafuroxin effects in Caucasian carriers of *LSS* AA and CC genotypes on OSBP changes and in H295R cells transfected with LSS A or C variants on LSS mRNA and EO levels. **A** OSBP fall (mmHg) in *LSS* AA and CC carriers treated with 50 mg losartan or 6, 50, and 500 µg rostafuroxin. Data adjusted for baseline are means ± SE. * ANCOVA (mean, 95% CI) 50 μg rostafuroxin *LSS* AA −25.2 (−34.3; −16.0) vs CC −12.2 (−19.6; −4.7), *P* = 0.034. **B** *LSS* mRNA levels in H295R transfected cells at baseline and incubated with different concentrations of rostafuroxin. Single data of each subgroup are represented by a dot. # *T* test at baseline (mean, 95% CI) major C 295.7 (222.6; 368.7) vs minor A 149.8 (87.3; 212.3), *P* = 0.003. ## *T* test in the presence of rostafuroxin 10^–11^ M, (mean, 95% CI) major C 141.3 (45.2; 237.5) vs minor A 442.0 (271.2; 612.8), *P* = 0.0028. **C** EO levels in H295R transfected cells incubated with 10^–11^ M rostafuroxin. Data are means ± SE. § *T* test at baseline (mean, 95% CI) major C 5.5 (3.6; 7.4) vs minor A 9.2 (6.0; 12.5), *P* = 0.038. §§ *T* test in the presence of rostafuroxin 10^–11^ M (mean, 95% CI) major C 5.2 (2.8; 7.5) vs minor A 6.6 (5.0; 8.2), *P* = 0.26.

To confirm previous findings [13], a pre-specified analysis was carried out on the pressor response to rostafuroxin and losartan in the context of *LSS* rs2254524 AA and CC genotypes alone and summarized in Fig. 2b. Compared to losartan, 50 μg rostafuroxin produced a larger OSBP fall in *LSS* AA (mean mmHg, (95% CI)) −13.4 (−25.4; −1.5) (*P* = 0.031) but a smaller one in *LSS* CC + 7.3 (−3.9; +18.6). The interaction between genotypes and drug responses was statistically significant (*P* = 0.023 for OSBP and *P* = 0.013 for ODBP). As in the P2a carriers, also in the *LSS* AA carriers, the OSBP responses to the three doses of rostafuroxin displayed a trend towards a bell shape curve, with the peak effect at the dose of 50 μg (Fig. 3a).

**Fig. 4:**
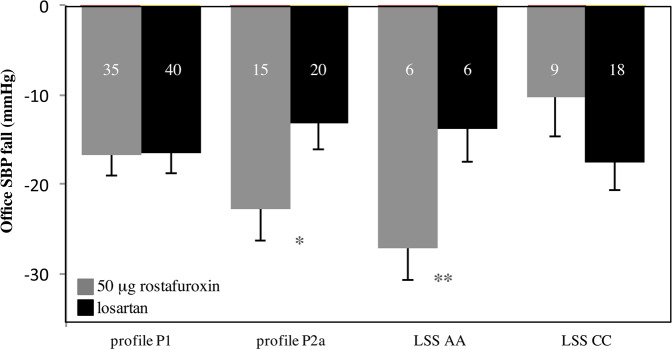
Summary of OSBP fall (mmHg) in 50 μg rostafuroxin and in losartan treatment arms across the different genetic backgrounds, Caucasian subgroups. Numbers in the columns refer to each subgroup sample size. ANCOVA test. Data adjusted for basal OSBP are means ± SE. * Δ = 9.8 mmHg OSBP for P2a, 50 mg losartan vs 50 μg rostafuroxin, *P* = 0.038; ** Δ = 13.4 mmHg OSBP for *LSS* AA, 50 mg losartan vs 50 μg rostafuroxin, *P* = 0.031.

Fig. 4 pools OSBP changes from Tables 2, 3 and Fig. 2b allowing a comparison among the various subsets of patients, according to their genotypes. The differential OSBP lowering effect of losartan and rostafuroxin was dependent upon the underlying genotypes.

Thirteen patients dropped out at visit 4 or before and six at visit 5. As shown in Table S6, one subject treated with 500 μg rostafuroxin reported a skin rash as adverse event. Detailed safety data for treatment-emergent adverse drug reactions in Caucasians and Chinese are reported in Table S7. No serious adverse events were reported in Caucasian, as described in Table S8, while one was present in Chinese and regarded an episode of severe vertigo.

### The OSBP changes to 50 μg rostafuroxin in *LSS* rs2254524 AA and CC carriers are similar to that of previous OASIS-HT trial

The previous OASIS-HT study aimed at assessing the hypotensive efficacy of rostafuroxin on individuals bearing specific allele combinations of the P2. Compared to PEARL-HT, it had lower duration of treatment (5 weeks) and placebo was used as a head-to-head comparator [13]. Furthermore, due to a low number of patients, the results of the rostafuroxin effect were provided by grouping the lower and the higher doses. To verify whether the preliminary results of the OASIS-HT trial could be confirmed in the present study, the individual OSBP data of the OASIS-HT patients treated with 50 μg rostafuroxin and carrying the P2 (6 patients) or the *LSS* AA (4 patients) and *LSS* CC (13 patients) genotypes were retrieved. These baseline values and changes after 5 weeks of treatment are reported in Table S9, where also the patients treated with losartan are included. Treatment with 50 μg rostafuroxin reached the OSBP target level of 135 mmHg in all 10 patients carrying the *LSS* AA genotype, but only in 4 out of 22 patients carrying the *LSS* CC genotype (Fig. 2c). It is noteworthy that a change of only one aminoacid, from Valine to Leucine at position 642 in the *LSS* protein, produced such a remarkable difference in the OSBP response to rostafuroxin. In Table S10 are shown the pooled OSBP responses to rostafuroxin 50 μg and to losartan 50 mg from the previous clinical trials [13] and the present PEARL-HT studies in the P2, *LSS* AA, and *LSS* CC Caucasian carriers. The level of statistical significance of the difference between rostafuroxin and losartan OSBP responses found in the present PEARL-HT study is increased in the pooled data. In particular, in the P2 carriers, unadjusted for genetic heterogeneity, the *P* values were 0.062 and 0.035 in the PEARL-HT and pooled data, respectively.

For the secondary end-points, ODBP, night- and 24h-ABPM, a statistically significant fall of BP was observed in the losartan and rostafuroxin 50 μg treated patients, but these BP changes were not different between the two treated groups, as reported in the online-only Supplementary Data.

### Rostafuroxin differently affects *LSS* mRNA level and EO production in human adrenocortical cells transfected with *LSS* rs2254524 variants

We previously showed that, compared to the cells transfected with the *LSS* major allele (C-642Val), the cells transfected with the minor allele (A-642Leu) have lower LSS mRNA levels and higher EO in the supernatant [13]. Here, we confirm these previous data. Compared to the major *LSS* C transfected cells values at baseline, the minor *LSS* A displayed lower levels of *LSS* mRNA (arbitrary units from 295 ± 49 to 149 ± 0.42; *P* = 0.003), associated with higher EO values (EO (Pm) per unit of *LSS* protein from 5.5 ± 0.8 to 9.2 ± 1.4; *P* = 0.038), as shown in Fig. 3b, c, respectively.

Incubation with rostafuroxin at increasing concentrations from 10^–12^ to 10^–8^ M for 24 h differentially affected mRNA concentrations in cells transfected with the major or the minor *LSS* alleles. In minor A background, the *LSS* mRNA increased according to a bell shape curve with a peak at 10^–11^ M rostafuroxin, while in the major C, at the same rostafuroxin concentration, mRNA level decreased (Fig. 3b). Furthermore, the baseline difference of EO levels observed between major C and minor A transfected cells (Fig. 3c) was abolished by incubation with rostafuroxin at 10^–11^ M.

## Discussion

The main findings of the present study were as follows: (1) Caucasians naïve patients carrying the genetic profile P2a or *LSS* AA genotype had larger OSBP fall to rostafuroxin 50 μg than to losartan; (2) rostafuroxin, in the H295R cells transfected with *LSS* A or *LSS* C allele, abolished the baseline increased EO production in the *LSS* A cells over that of *LSS* C cells, and enhanced the depressed levels of *LSS* mRNA in the former cells, thus demonstrating a causal link between genotypes and rostafuroxin’s effect also at cellular levels.

The efficacy of rostafuroxin in the P2a and *LSS* AA Caucasians carriers was similar to that observed in the previous OASIS-HT trial [13]; therefore, the present results may be considered a replication of previously published results [13]. Moreover, the OSBP differences between rostafuroxin and losartan were larger than those described in previous studies where a head-to-head comparison among antihypertensive drugs was tested [28, 29] and, most importantly, they were genotype dependent. These OSBP changes were not associated with any serious adverse event, while the adverse events were not different from those of the comparator that was placebo in the OASIS-HT e and losartan in the present trial.

The dose–effect relationship of rostafuroxin tended to have a bell-shaped trend, particularly in carriers of *LSS* AA, having a peak effect at 50 μg. A similar trend towards a bell-shape curve was observed in the previous published OASIS-HT study [13] and is consistent with the previous and present data obtained in cell-free system and isolated rat and human transfected cells [2, 5, 12, 13]. In those settings, rostafuroxin selectively inhibits the altered biochemical step common to both mutant adducin and ouabain pressor mechanisms at 10^–10^ or 10^–11^ M, being this effect lower both at higher and lower concentrations. Moreover, there is a plausible correspondence between the very small daily oral dose of 50 μg (equal to 0.134 moles), with the previous [5, 12] and present concentration of 10^–11^ M shown to be effective in human cells or with the doses reducing BP in the animal model [5].

The following arguments support the appropriateness of the available data to move to the phase 3 studies. Both OASIS-HT and PEARL-HT trials had baseline OSBP around 150 mmHg and the degree of OSBP reduction produced by 50 μg rostafuroxin ranged from 23.0 to 27.3 mmHg in either case in carriers of the “pre-specified” profile, which is of remarkable clinical significance for a drug acting on a causal mechanism of hypertension. This observation strengthens the choice of the dose of 50 μg to move to phase 3 trial.

Both rat and human mutant α-adducin and ouabain increase of Na-K pump units on the cell surface of renal tubular cells leading to an enhancement of renal sodium reabsorption [9–11, 16, 17]. Moreover, past [2, 4, 5, 12] and present findings show that rostafuroxin is able to selectively antagonize the effects of mutant α-adducin and low concentrations of ouabain in vitro at concentration of 10^–11^ M, in rodent and human settings and in vivo, in a rodent model, without interacting with 35 others targets including hormones receptors or proteins involved in cardiovascular regulation up to concentration of 10^–5^ M.

Since rostafuroxin acts by selectively blocking a molecular mechanism common to both hypertension and organ damage [2, 4, 12, 30–32], for any given BP level, it may be endowed with an organ damage protection greater than the 20–30% achievable with the available drugs.

The magnitude of the clinical and costs benefits may be inferred from the frequencies of the *LSS* AA genotype and P2a in Caucasians, about 10% and 20%, respectively.

The major limitation of this study is the lack of BP response to rostafuroxin of Chinese patients being the magnitude of the losartan BP response similar to that of Caucasians. As discussed in the online Supplementary Data, in all the Randomized Controlled Trials (RCTs), the placebo treatment is associated with a significant BP fall ranging from 6 to 9 mmHg [33]. The present findings suggested that in the Chinese P2 carriers the potential placebo effect associated to the rostafuroxin capsules administration was inhibited. Similarly, in the OASIS-HT study in Caucasian [13], placebo capsules significantly reduce BP in the general population of hypertensives, but they have no effect in carriers of the P2. The most logical explanation for this intriguing observation is to admit a faster metabolism or lower bioavailability of rostafuroxin in Chinese patients that were partially overridden by the dose of 500 μg.

Although the present results on Caucasian patients were obtained in a rather small sample size, they however showed a large effect size and replicates the results of OASIS-HT [13]. Moreover, according to Goodman [34] and Munafò [35], the degree of consistency of the present results with the previous ones discussed above, obtained both in humans and animals in different experimental and clinical settings, may strengthen the credibility of the present data.

If the present findings that are consistent with those of other two previous trials involving overall 658 Caucasian hypertensive patients were confirmed by the next phase 3 trial, they may contribute to the development of precision medicine in Caucasian patients with primary hypertension [36].

## Supplementary information

Supplementary Legends

Supplemental Material

Text S1

Text S2

Text S3
