# Supplementary material for: Antihypertensive treatment guided by genetics: PEARL-HT, the randomized proof-of-concept trial comparing rostafuroxin with losartan
Source: Pharmacogenomics J. 2021 Mar 1;21(3):346–58. doi: 10.1038/s41397-021-00214-y (PMC8159753; doi:10.1038/s41397-021-00214-y)
Supplement: Supplementary file 1 — Supplementary Legends [file 41397_2021_214_MOESM1_ESM.docx]

**Text S1.** PEARL-HT Protocol.

**Text S2.** PEARL-HT Statistical Analysis Plan (SAP).

**Text S3.** PEARL-HT Data Review Report (DRR).

**Table S1. Studies centers for PEARL-HT.**

**Table S2.** **Single nucleotide polymorphisms composing the genetic profile 1 (P1) in randomized Caucasian and Chinese population.**

**Table S3. Baseline characteristics of Caucasian patients included in the profiles P2, P2a, and in *LSS* AA and *LSS* CC subgroups.** Data are means (±SD) and range. Data are mean (SD), range, except for Gender (M/F) n (%).

**Table S4. Baseline characteristics of Chinese patients included in the profiles P2, P2a, and in *LSS* AA and *LSS* CC subgroups.** Data are means (±SD) and range.

**Table S5. OSBP fall and treatment differences (mmHg) in Caucasian carriers of the profile P2a treated with 6 μg, 50 μg, 500 μg rostafuroxin and 50 mg losartan.** ANCOVA test. Data (means and 95% CI) are delta OSBP adjusted for baseline.

**Table S6. Dropout patients with OSBP level at various time point and dropout reason.** Data are means (mmHg).

**Table S7.** **Treatment-Emergent Adverse Drug Reactions (TEADR) by System Organ Class and Preferred Term - Caucasian subgroup and Chinese subgroup.** Data are number (%). An adverse drug reaction is an adverse event judged as suspected to be study drug related.

**Table S8. Summary of Treatment-Emergent Adverse Events (TEAEs) in Caucasian PEARL-HT.** Adverse events with onset date ≥ date of first randomized study drug intake are presented in this table. SAE, Serious Adverse Event; ADR, Adverse Drug Reaction. Percentages are calculated on the number of patients (N).

**Table S9. Individual and mean OSBP values (mmHg) at baseline and after five weeks treatment for profile P2, and *LSS* AA_CC carriers.** Means are (±SD) detected in OASIS-HT study for rostafuroxin 50 μg [1], and in SOPHIA study for losartan [1,4].

**Table S10.** **Baseline and adjusted SBP change from baseline after 5 (OASIS-HT) or 9 (PEARL-HT) weeks of treatment in combined OASIS-HT/PEARL-HT studies.** Baseline means are (±SD) detected in OASIS-HT study for rostafuroxin 50 μg [1], and in SOPHIA study for losartan [1,4]. For OSBP changes data are adjusted for baseline (means and 95% CI).

**Table S11. Office DBP baseline values and changes based on treatment in carriers of P1 and P2 for Caucasian (IT) and Chinese (TW).** Delta means unadjusted for the baseline DBP values. Data from ANOVA are means ± SD. --, not determined. P1 IT, * *P*=0.003; ** *P*<0.001. P2 IT, # *P*=0.018; ## *P*=0.002; ### *P*<0.001; P1 TW, § *P*>0.5; §§ *P*<0.001; P2 TW, @ *P*>0.5; @@ *P*=0.004.
