# Supplementary material for: Antihypertensive treatment guided by genetics: PEARL-HT, the randomized proof-of-concept trial comparing rostafuroxin with losartan
Source: Pharmacogenomics J. 2021 Mar 1;21(3):346–58. doi: 10.1038/s41397-021-00214-y (PMC8159753; doi:10.1038/s41397-021-00214-y)
Supplement: Supplementary file 2 — Supplemental Material [file 41397_2021_214_MOESM2_ESM.docx]

**Online Supplementary Data**

**Antihypertensive treatment guided by genetics: PEARL-HT, the randomized proof-of-concept trial comparing rostafuroxin with losartan**

Lorena Citterio, MSc^1,2^; Giuseppe Bianchi, MD ^2,^*; Giuseppe A. Scioli, MD^3^; Nicola Glorioso, MD^4^; Roberto Bigazzi, MD^5^; Daniele Cusi, MD^6,7^; Jan A. Staessen, MD^8^; Silvio Cavuto, MSc^9^; Mara Ferrandi, MSc^10^; Chiara Lanzani, MD^1,2^; Xiaoyi Li, MD^11^; Lit-Fui Lau, PhD^12^; Chern-En Chiang, MD^13^; Tzung-Dau Wang, MD^14^; Kang-Ling Wang, MD^13^; Patrizia Ferrari, PhD^10^; Paolo Manunta, MD^1,2^

**Affiliations:**

^1^Genomics of Renal Diseases and Hypertension Unit, IRCCS San Raffaele Scientific Institute, Università Vita Salute San Raffaele, Milano, Italy

^2^Università Vita Salute San Raffaele, Milan, Italy

^3^Hypertension and Cardiovascular Prevention Center, Ospedale Ferdinando Veneziale, Isernia, Italy ^4^Hypertension and related diseases Center, Department of Clinical and Experimental Medicine, University of Sassari, Sassari, Italy

^5^Nephrology and Dialysis Unit, Livorno, Italy

^6^Institute of Biomedical Technologies Milano National Research Council of Italy (CNR), Segrate, Milano, Italy

^7^Bio4Dreams Scientific Unit, Bio4Dreams - Business Nursery for Life Sciences, Milano, Italy

^8^Research Unit Hypertension and Cardiovascular Epidemiology KU Leuven Department of Cardiovascular Sciences, University of Leuven, Leuven, Belgium

^9^Clinical Trials and Statistics Unit, Azienda USL - IRCCS di Reggio Emilia, Reggio Emilia, Italy

^10^Windtree Therapeutics, Warrington, PA, USA

^11^CVie Therapeutics, Taipei, Taiwan

^12^Zhaoke (Guangzhou) Ophthalmology Pharmaceutical Limited, Guangzhou, China

^13^General Clinical Research Center, Taipei Veterans General Hospital and National Yang-Ming University, Taipei, Taiwan

^14^Cardiovascular Center and Division of Cardiology, Department of Internal Medicine, National Taiwan University Hospital and College of Medicine, National Taiwan University, Taipei, Taiwan

**Short title: Modulation of rostafuroxin and losartan by genetics**

* Corresponding author:

Giuseppe Bianchi; MD

Università Vita Salute San Raffaele, Milan, Italy

Tel +39 0226435330, Fax +39 02 2643 2384

E-mail: [bianchi.giuseppe@hsr.it](mailto:bianchi.giuseppe@hsr.it)

**METHODS**

**Central genotyping**

All genomic DNA samples were loaded at 50 ng/μL. For analysis of the genotypes, we used auto calling methods as implemented in the TaqMan Genotyper software version 1.3 (Life Technologies). Next, genotype clusters were evaluated manually. Duplicate samples gave a reproducibility rate of 100%. Genotyping was performed within three days from sample reception for a quick genetic profile determination, essential for selecting the eligible patients for randomization. Both the Caucasian and the Chinese patients to be enrolled were screened for the P1. P1, P2 and were then analysed separately.

Three Single Nucleotide Polymorphisms (SNPs) on *ADD3*, *ADD2* and *HSD3B1* genes used in Caucasians in OASIS-HT study were not present in the Chinese (Table 1). Therefore, we set-up a study to detect other SNPs on the same genes present in both populations able to predict the BP response to rostafuroxin in Caucasians of the OASIS-HT study. Only for *HSD3B1* region we could not detect any SNP common to either populations. Thus, we selected *HSD3B1* rs117585927, which is unique for Chinese and maps only 117 bp from rs10923835, the Caucasian SNP (Table 1). Minor allele frequency and Hardy-Weinberg equilibrium for both Caucasian and Chinese randomized populations are reported in Table S2.

**Randomization**

Assignment to treatment groups was determined by a computer-generated random sequence using an interactive web-based response system (IWRS) provided by the CRO involved in the study. The randomization was stratified by country (i.e. Taiwan for Chinese population and Italy for Caucasian), by genetic subgroup (one stratum was constituted by the patients bearing a mutation included in the P2, the other stratum comprised all the remaining patients included into the P1 group). Blocking was used to keep the balance of the treatment allocations.

**Masking**

To preserve study blinding, access to the randomization table and treatment assignments was restricted to the responsible of pharmacovigilance for each country and to the manufacturers responsible for drug packaging prior to database lock.

The investigators were allowed to break individual codes exclusively when a serious adverse event occurred and knowledge of the treatment by the investigator was deemed useful as to the patient’s safety. After unblinding, the subject was discontinued from the study.

Breaking of individual randomization codes was also allowed to the drug safety unit of CVie Therapeutics, or its pharmacovigilance responsible delegate during the course of the study, in order to comply with the regulatory requirements, to the Data and Safety Monitoring Board (DSMB), in order to give advice to the sponsor in case of safety concerns.

**Choice of naïve patients and losartan as a comparator**

The well-known greater variability of BP at the initial stages of hypertension may affect the precision of the BP response to drug evaluation in a relatively short duration phase 2 study. In spite of this drawback, two independent reasons supported the choice of naïve patients for this study aimed at exploring the relationship between hypertension and its plausible genetic triggering primary mechanisms. First, the washout period of 15-30 days in already treated patients is not long enough to get rid of the persistency of the previous drug effects both on the related phenotypes and the corresponding gene expression [1]. Second, it has been clearly demonstrated that in secondary forms of hypertension triggered by well-known “causal” mechanisms, such as mono-lateral renal artery stenosis [2] or mono-lateral adrenal adenoma [3], the surgical removal of this “causal” mechanism may “cure” hypertension better when carried out at the initial stages than at the later ones, after the development of overt vascular or renal damages. These findings must be taken into account when testing the effect of a selective inhibitor of gene triggering mechanisms to prove their “causal” role.

Also the choice of losartan, as comparator, is supported by two reasons: 1) since this study included an initial run-in period with lifestyle changes, followed by a subsequent study period of two months, a placebo arms could not have been approved by some ECs; 2) the just completed SOPHIA study on losartan in naïve patients [4], whose clinical design is similar to that of the previous OASIS-HT study [1], furnished preliminary data for planning the present PEARL-HT study. Standing the well-established relationship between the body sodium and the pressor response to angiotensin II that increases at higher body sodium, the losartan efficacy may also be potentiated by gene variants that, by increasing renal tubular reabsorption, may enhance body sodium. This limits the validity to assess causation from the difference in the BP response, that may also regard any of the available antihypertensive drugs, acting on physiological mechanisms involved in BP regulation.

**The definition of confounders and the distinction between primary and secondary variables.**

The definition of confounders should be based on assumptions that need to be proved or plausible [5]. Also the most common adjustment for baseline level of OSBP may be influenced by the genetic background, when BP response to drugs is compared. In fact, in patients carrying genetic abnormalities leading to an increase of sodium reabsorption supported by, 1) a sodium channel increased activity at the kidney level or 2) aldosterone secretion by the adrenal glands, the magnitude of the BP fall after amiloride [6] or spironolactone [7], respectively, is much greater than that achieved with other drugs, independently from the baseline BP levels.

Considering the previous knowledge on the rostafuroxin BP effects in Caucasian population, two factors play a crucial role: 1) the genetic background and 2) the baseline BP values. For the former, a long list of published experimental and clinical findings [1,8-11] indicate that the gene variants affecting adducin and ouabain effects, together with their gene modifiers, if any, greatly influence the BP responses to rostafuroxin. The 24-hour Ambulatory Blood Pressure (24h-ABPM) was considered a secondary variable because we are dealing with a limited subset of newly discovered and never-treated (naïve) hypertensive patients, at an initial grade 1 type of hypertension, who have been selected according to an OSBP level >140 mmHg and ODBP >85 mmHg. Due to the well-known component of white coat hypertension, that particularly affects office BP levels at the initial stages (or lower grades) of hypertension [12], a portion of these patients may have a normal 24h-ABPM, particularly during the night that is considered the most important predictor for future CV events [13]. This issue must be particularly considered in view of the rostafuroxin action mechanism. In fact, rostafuroxin, differently from almost all the available antihypertensive drugs, blocks an underlying cause of hypertension without affecting the physiological mechanisms controlling BP. This implies that rostafuroxin may correct an abnormal BP level without further reducing an already “normal” level. For all these reasons patients with baseline level of SBP <120 or <130 mmHg for the night- or the 24h-ABPM, respectively, have been excluded, because these are values at the threshold between “normal” and “abnormal” BP levels [14-16]. Overall, 30 patients carrying the P2 were excluded both because the above reason and the exclusion criteria listed in the protocol (Text S1) and shown in the Data Review Report (DRR) (Text S3). Thus, due to this very small sample size, the validity of the comparison among arms of the 24h-ABPM variable was greatly reduced.

Being the patients selected in a range of ODBP between 85-100 mmHg, the corresponding mean baseline ODBP may be around 91-93 mmHg, that is only 6-8 mmHg above the considered normal ODBP threshold of 85 mmHg. Therefore, rostafuroxin has a smaller room to exploit its effect, thus also ODBP was considered a secondary variable.

***LSS* expression analysis and endogenous ouabain quantification in H295R cells**

Transfection with *LSS* major (C-642Val) and minor (A-642Leu) variants was investigated in human adrenocortical cells (H295R cells), as previously described [1]. H295R cells have been obtained from ATCC (NCI¬H295R [H295R] (ATCC® CRL¬2128™). Cells have been authenticated by STR profile and tested for mycoplasma contamination. For the H295R cell culture transfection and analysis, an expression vector for *LSS* (642Val variant, obtained from ImaGenes GmbH) was mutagenized with oligonucleotide 5'-cgaggagcggcgttatttgcagagtgcc to generate the minor variant 642Leu. H295R adrenocortical cells were transiently transfected with Nucleofector technology (Amaxa Biosystems). Three million cells were electrophoresed with 4 μg of recombinant plasmids and collected after 48 hours for mRNA and protein quantification. Quantitative target mRNA level for *LSS* was quantified by real-time PCR detection by using specific primers (forward: 5'-ttgcttcacctacggcacct and reverse: 5'-aggaagtcacaggcccgg) and MGB probe (5'-taccgagatgggactgc). Target *LSS* mRNA levels were normalized to GAPDH and expressed as arbitrary units.

For Western blotting analysis, 5 μg of total protein from H295R cells was lysed with Laemmli sample buffer and separated by SDS–polyacrylamide gel electrophoresis (Criterion XT, Bio-Rad), blotted on nitrocellulose membrane (Bio-Rad) for 90 minutes and incubated overnight at 4°C with specific primary antibodies, followed by 1 hour incubation with fluorescent secondary antibody (Alexa Fluor). The primary anti-ouabain polyclonal antibody used in the present study was an home-made antibody produced by Prassis, Settimo Milanese, Milano, Italy [1]. Western blotting was analyzed and quantified by Odyssey Infrared Imaging Detection System (LI-COR Biosciences). Quantitative analysis of the fluorescent signal for *LSS* and GAPDH protein was reported as integrated intensity of the fluorescent band. The densitometry analysis for *LSS* protein expression was normalized to GAPDH and expressed as arbitrary units. The primary antibodies for *LSS* and *GAPDH* were both from Santa Cruz Biotechnology Inc and were the following: *LSS* (OSC G-18, sc-83294); *GAPDH* (6C5, sc-32233).

EO was quantified in the cultured medium of H295R cells. Cells were serum-starved for 24 hours after transfection with 1:10 diluted medium and incubated for an additional 72 hours. The supernatant from 12 × 10^6^ transfected cells was collected for EO quantification. Samples were homogenized with methanol (ratio 1:4) and stirred overnight at 4°C, then centrifuged at 1500 g for 30 minutes and the supernatant dried under vacuum, as described [17]. The dried samples were reconstituted with 0.1% TFA and passed by vacuum over C18 bond eluted columns (Varian). Several washes with water and one wash with 2.5% acetonitrile were performed and EO was finally eluted from C18 columns with 25% acetonitrile. The eluate was dried, reconstituted with phosphate buffer and tested in a RIA assay, as described [17]. Briefly, extracted samples or commercial standard ouabain (Sigma Aldrich) were incubated with ^3^H-ouabain (2 nM, Amersham) and a previously characterized, highly specific, anti-ouabain polyclonal antibody for 15 hours. The incubation was stopped by filtration and radioactivity counted. EO levels were expressed as picomoles per liter and normalized for *LSS* protein expression.

The effect of rostafuroxin on *LSS* mRNA and EO levels, was evaluated in H295R cells incubated from the beginning of *LSS* gene transfection, in a cultured medium containing rostafuroxin from 10^-12^ to 10^-8^ M or vehicle. At the end of the incubation, the cultured medium was collected for EO measurement and the pellet containing H295R cells were used for *LSS* mRNA or *LSS* protein quantification.

**RESULTS**

**Office diastolic blood pressure (ODBP) changes are lower than the OSBP changes but with similar trends**

In carriers of P1 and P2, rostafuroxin lowered ODBP in Caucasians but not in Chinese (Table S11). In Caucasians carrying the P2a, the ODBP decrease was: (mean mmHg, (95% CI), number of patients) -8.0 (-11.7; -4.2), n=20, *P*<0.001 and -11.0 (-15.3; -6.6), n=15, *P*=0.002 in the losartan and rostafuroxin 50 𝜇g groups, respectively, being the differences between the treatment groups non-significant, *P*=0.30. In *LSS* AA carriers, the changes in ODBP (Fig. 2B) were similar to those of OSBP but did not reach the statistical significance (*P*=0.095), while the interaction between *LSS* genotypes and the ODBP fall with losartan vs. rostafuroxin 50 𝜇g was statistically significant (*P*=0.013).

The 24h-ABPM changes did not differ between rostafuroxin and losartan treatment in Caucasian carriers of P2a. The 24-hour SBP changes from the baseline values in the four arms were: (mean mmHg, (95% CI), number of patients, *P* value) -0.8, (-7.5 +5.8), 14, *P*>0.5; -6.8, (-14.9 +1.2), 10, *P*=0.094; -9.4, (-15.8, -3.1), 14, *P*=0.005; and -7.6, (-14.2, -1.0), 13, *P*=0.025) for rostafuroxin 6, 50, 500 μg and losartan 50 mg, respectively. The corresponding night SBP changes were: -2.2, (-12.8, +8.4), 8, *P*>0.5; -12.5, (-23.0, -2.0), 9, *P*=0.021; -10.4, (-20.5, -0.4), 9, *P*=0.042; and -11.1, (19.4, -2.8), 13, *P*=0.011. No statistical difference in the SBP changes was detected between the rostafuroxin 50 𝜇g and losartan *P*>0.5. As described in the methods session of primary and secondary variables, the remarkable reduction in the sample sizes of the 4 arms is explained in the specific section in the Methods (Definition of confounders, distinction between primary and secondary variables and statistical analysis).

**Table S1. Studies centers for PEARL-HT.**

| **ITALY** |  |  |
| --- | --- | --- |
| *Center N* | *Principal Investigator* | *Site* |
| 101 | Paolo Manunta | Università Vita-Salute San Raffaele Hospital Milano |
| 104 | Francesco Perticone | Policlinico Universitario Campus Germaneto Catanzaro |
| 105 | Giuseppe Antonio Scioli | Centro per l'Ipertensione - Ospedale F.Veneziale Isernia |
| 107 | Roberto Bigazzi | U.O. Nefrologia e Dialisi Spedali Riuniti Livorno |
| 108 | Flavio Scanferla | Emodialisi Ospedale dell'Angelo Mestre |
| 109 | Ferruccio Galletti | Medicina Clinica e Chirurgia - Università Federico II Napoli |
| 110 | Francesco Fallo | Clinica Medica 3 Università di Padova |
| 112 | Angelo Rigotti | U.O. Nefrologia e Dialisi - Ospedale degli Infermi di Rimini |
| 113 | Nicola Glorioso | Centro per l'Ipertensione - A.S.L. n.1 Sassari |
| 114 | Paolo Mulatero | S.C.U. Medicina Interna 4 - Città della Salute e della Scienza Torino |
| 115 | Leonardo Sechi | Dipartimento di Medicina Interna - A. Ospedaliero Universitaria Udine |
| 116 | Francesco Cocco | Divisione di Cardiologia e UTIC Ospedale "Marianna Giannuzzi" Manduria (TA) |
| 117 | Goffredo del Rosso | U.O. Nefrologia e Dialisi Presidio Ospedaliero "Giuseppe Mazzini" Teramo |
| **TAIWAN** |  |  |
| *Center N* | *Principal Investigator* | *Site* |
| 201 | Kang-Ling Wang | Taipei Veterans General Hospital |
| 202 | Zhih-Cherng Chen | Chi-Mei Medical Center |
| 204 | Tsung-Hsien Lin | Kaohsiung Medical University Chung-Ho memorial Hospital |
| 205 | Kuan-Cheng Chang | China Medical University Hospital |
| 206 | I-Chang Hsieh | Chang Gung Medical Foundation – Linkou Branch |
| 207 | Wei-Hsian Yin | Cheng Hsin General Hospital |
| 208 | Ming-En Liu | Mackay Memorial Hospital – Hsinchu Branch |
| 209 | Kwo-Chang Ueng | Chung Shan Medical University Hospital |
| 210 | Hung-I Yeh | Mackay Memorial Hospital-Tamshui Branch |
| 213 | Ting-Hsing Chao | National Cheng Kung University Hospital |
| 214 | Tzung-Dau Wang | National Taiwan University Hospital |
| 215 | Chih-Cheng Wu | National Taiwan University Hospital – Hsin-Chu Branch |
| 216 | Yen-Wen Wu | Far Eastern Memorial Hospital |
| 217 | Wen-Chin Ko | Cathay General Hospital |
| 219 | Chang-Min Chung | Chang Gung Medical Foundation - Chiayi |

**Table S2. Single nucleotide polymorphisms composing the genetic profile 1 (P1) in randomized Caucasian and Chinese population.**

SNPs are ranked by chromosome and position based on GRCh38 (release 108) assembly. SNPs included in the genetic profile 2 (P2) are in bold, while those specific to Chinese are in italics. MAF, Minor Allele Frequency; IT, PEARL-HT Italy (Caucasian); TW, PEARL-HT Taiwan (Chinese); H-W, Hardy-Weinberg equilibrium.

*Gene name or flanking region (±500 Kb)

**Pearson *P* value

**Table S3**. **Baseline characteristics of Caucasian patients included in the profiles P2, P2a, and in *LSS* AA and *LSS* CC subgroups.** Data are means (±SD) and range. Data are mean (SD), range, except for Gender (M/F) n (%).

| **Genetic background (frequency)** | **treatment** | **Age (years)** *mean (SD) range* | **Gender (M/F)** *n (%)* | **Height**  **(cm)***  *mean (SD) range* | **Weight (kg)*** *mean (SD) range* | **BMI (kg/m2)** *mean (SD) range* | **Time to disease**  **onset (months)**  *mean (SD) range* | **Potassium (mEq/L)**  mean (SD) range | **Creatinine (mg/dL)**  mean (SD) range |
| --- | --- | --- | --- | --- | --- | --- | --- | --- | --- |
| **P2** | *6 μg rostafuroxin (n=20)* | 49.0 (7.0) 30-59 | 11 (55.0%) / 9 (45.0%) | 167.6 (8.9) 148-180 | 72.6 (12.0) 55-91 | 25.8 (2.9) 21-30 | 17.0 (30.4) 1-128 | 4.3 (0.27) 3.9-4.8 | 0.77 (0.12) 0.5-0.9 |
|  | *50 μg rostafuroxin (n=17)* | 46.7 (7.2) 31-56 | 11 (64.7%) / 6 (35.3%) | 171.0 (7.4) 157-181 | 79.3 (8.9) 60-93 | 27.1 (2.5) 23-30 | 11.1 (14.3) 2-60 | 4.4 (0.33) 4.0-5.2 | 0.75 (0.20) 0.5-1.2 |
|  | *500 μg rostafuroxin (n=19)* | 48.2 (5.6) 38-56 | 14 (73.7%) / 5 (26.3%) | 172.6 (7.8) 159-186 | 79.7 (13.3) 59-100 | 26.6 (2.7) 21-30 | 18.6 (36.8) 1-130 | 4.4 (0.30) 3.7-4.9 | 0.80 (0.16) 0.5-1.1 |
|  | *losartan (n=23)* | 50.7 (7.9) 29-62 | 19 (82.6%) / 4 (17.4%) | 174.8 (9.3) 158-192 | 81.4 (13.9) 50-106 | 26.5 (3.1) 17-30 | 18.0 (28.2) 1-125 | 4.3 (0.46) 3.7-5.2 | 0.88 (0.24) 0.5-1.5 |
| **P2a** | *6 μg rostafuroxin (n=16)* | 48.6 (7.5) 30-59 | 8 (50.0%) /  8 (50.0%) | 166.6 (9.7) 148-180 | 71.9 (13.0) 55-91 | 25.8 (3.0) 21-30 | 20.3 (33.4) 1-128 | 4.4 (0.27) 3.9-4.8 | 0.76 (0.12) 0.5-0.9 |
|  | *50 μg rostafuroxin (n=15)* | 46.9 (7.1) 31-56 | 9 (60.0%) /  6 (40.0%) | 170.2 (7.5) 157-181 | 78.0 (8.7) 60-93 | 26.9 (2.6) 23-30 | 10.5 (14.7) 2-60 | 4.5 (0.34) 4.0-5.2 | 0.73 (0.21) 0.5-1.2 |
|  | *500 μg rostafuroxin (n=18)* | 48.4 (5.7) 38-56 | 14 (77.8%) / 4 (22.2%) | 173.2 (7.7) 159-186 | 80.2 (13.5) 59-100 | 26.6 (2.8) 21-30 | 19.4 (37.7) 1-130 | 4.3 (0.30) 3.7-4.9 | 0.81 (0.16) 0.5-1.1 |
|  | *losartan (n=20)* | 50.3 (8.2) 29-62 | 16 (80.0%) / 4 (20.0%) | 173.5 (8.8) 158-185 | 78.8 (12.6) 50-100 | 26.1 (3.1) 17-30 | 17.4 (28.7) 1-125 | 4.4 (0.45) 3.7-5.2 | 0.88 (0.25) 0.5-1.5 |
| ***LSS* AA** | *6 μg rostafuroxin (n=6)* | 48.2 (5.8) 40-57 | 4 (66.7%) /  2 (33.3%) | 170.0 (5.6) 160-175 | 73.8 (6.7) 65-84 | 25.6 (2.1) 23-28 | 4.1 (1.0) 3-5 | 4.2 (0.24) 4.0-4.5 | 0.76 (0.15) 0.5-0.9 |
|  | *50 μg rostafuroxin (n=6)* | 46.5 (10.7) 31-61 | 3 (50.0%) /  3 (50.0%) | 170.3 (7.8) 156-179 | 74.2 (13.4) 57-90 | 25.5 (3.5) 20-30 | 25.6 (54.8) 1-137 | 4.4 (0.22) 4.1-4.7 | 0.81 (0.18) 0.6-1.1 |
|  | *500 μg rostafuroxin (n=8)* | 50.1 (5.4) 41-56 | 5 (62.5%) /  3 (37.5%) | 169.5 (11.5) 160-193 | 74.6 (15.9) 58-109 | 25.7 (2.8) 23-30 | 12.7 (18.7) 1-58 | 4.5 (0.28) 4.1-4.8 | 0.79 (0.17) 0.6-1.2 |
|  | *losartan (n=6)* | 49.8 (7.1) 41-59 | 5 (83.3%) /  1 (16.7%) | 172.0 (11.2) 156-185 | 83.0 (16.7) 60-101 | 27.8 (2.7) 24-30 | 4.1 (1.2) 2-6 | 4.4 (0.32) 3.9-4.9 | 0.75 (0.12) 0.5-0.9 |
| ***LSS* CC** | *6 μg rostafuroxin (n=16)* | 45.9 (8.8) 30-59 | 9 (56.3%) /  7 (43.8%) | 167.7 (6.9) 155-178 | 72.3 (10.8) 57-93 | 25.6 (3.1) 20-29 | 18.8 (35.6) 1-130 | 4.3 (0.29) 3.8-4.9 | 0.82 (0.18) 0.6-1.1 |
|  | *50 μg rostafuroxin (n=9)* | 47.8 (6.8) 35-54 | 4 (44.4%) /  5 (55.6%) | 165.6 (6.6) 153-176 | 73.1 (9.8) 60-92 | 26.6 (2.7) 23-30 | 15.4 (18.9) 2-60 | 4.3 (0.30) 3.7-4.8 | 0.69 (0.21) 0.4-1.1 |
|  | *500 μg rostafuroxin (n=16)* | 51.1 (5.2) 43-59 | 13 (81.3%) / 3 (18.8%) | 170.4 (7.6) 153-182 | 77.4 (10.7) 50-90 | 26.6 (3.1) 21-30 | 17.2 (32.2) 1-130 | 4.4 (0.31) 4.0-5.0 | 0.84 (0.10) 0.6-1.0 |
|  | *losartan (n=18)* | 50.4 (7.2) 35-59 | 16 (88.9%) / 2 (11.1%) | 174.0 (9.1) 162-198 | 78.1 (14.4) 50-106 | 25.7 (3.4) 17-30 | 13.2 (15.7) 1-56 | 4.4 (0.47) 3.7-5.8 | 0.90 (0.18) 0.5-1.3 |

**Table S4. Baseline characteristics of Chinese patients included in the profiles P2, P2a, and in *LSS* AA and *LSS* CC subgroups.** Data are means (±SD) and range, except for Gender (M/F) n (%).

| **Genetic background (frequency)** | **treatment** | **Age (years)** *mean (SD) range* | **Gender (M/F)** *n (%)* | **Height**  **(cm)***  *mean (SD) range* | **Weight (kg)*** *mean (SD) range* | **BMI (kg/m2)** *mean (SD) range* |
| --- | --- | --- | --- | --- | --- | --- |
| **P2** | *50 μg rostafuroxin (n=14)* | 45.5 (6.4) 33-57 | 10 (71.4%) / 4 (28.6%) | 166.2 (10.8) 150-183 | 71.7 (12.4) 48-90 | 25.8 (2.6) 20-29 |
|  | *500 μg rostafuroxin (n=15)* | 47.5 (9.7) 28-60 | 11 (73.3%) / 4 (26.7%) | 167.8 (9.9) 144-182 | 74.6 (12.3) 44-89 | 26.3 (3.1) 21-30 |
|  | *losartan (n=16)* | 47.6 (7.7) 36-60 | 8 (50.0%) /  8 (50.0%) | 162.2 (7.8) 151-175 | 67.0 (12.9) 44-85 | 25.3 (3.4) 18-30 |
| **P2a** | *50 μg rostafuroxin (n=13)* | 45.2 (6.6) 33-57 | 9 (69.2%) /  4 (30.8%) | 165.5 (10.9) 150-183 | 71.2 (12.8) 48-90 | 25.8 (2.7) 20-29 |
|  | *500 μg rostafuroxin (n=9)* | 48.3 (10.0) 33-60 | 8 (88.9%) /  1 (11.1%) | 170.0 (6.3) 160-182 | 79.1 (6.4) 68-89 | 27.4 (2.6) 24-30 |
|  | *losartan (n=12)* | 48.0 (8.4) 36-60 | 6 (50.0%) /  6 (50.0%) | 162.3 (8.1) 151-175 | 67.6 (14.0) 44-85 | 25.5 (3.8) 18-30 |
| ***LSS* AA** | *50 μg rostafuroxin (n=1)* | 57.0 (--)  -- | -- (--) /  1 (100.0%) | 152.0 (--) -- | 53.2 (--) -- | 23 (--)  -- |
|  | *500 μg rostafuroxin (n=6)* | 48.3 (5.8) 40-55 | 2 (33.3%) /  4 (66.7%) | 161.4 (11.9) 144-176 | 66.1 (15.7) 44-85 | 25.0 (3.3) 21-29 |
|  | *losartan (n=5)* | 44.8 (3.3) 38-52 | 2 (40.0%) /  3 (60.0%) | 161.5 (6.7) 152-171 | 64.4 (9.1) 56-80 | 24.6 (1.7) 23-27 |
| ***LSS* CC** | *50 μg rostafuroxin (n=11)* | 44.7 (4.9) 37-52 | 5 (45.5%) /  6 (54.5%) | 167.6 (13.3) 150-186 | 72.5 (15.9) 48-94 | 25.6 (3.4) 20-30 |
|  | *500 μg rostafuroxin (n=16)* | 41.9 (8.7) 29-60 | 10 (62.5%) / 6 (37.3%) | 167.9 (8.7) 153-183 | 74.7 (12.1) 51-98 | 26.4 (3.5) 19-30 |
|  | *losartan (n=15)* | 45.0 (9.3) 31-58 | 7 (46.7%) /  8 (53.3%) | 165.5 (9.4) 152-181 | 74.7 (13.5) 53-95 | 27.1 (3.1) 21-30 |

**Table S5. OSBP fall and treatment differences (mmHg) in Caucasian carriers of the profile P2a treated with 6 μg, 50 μg, 500 μg rostafuroxin and 50 mg losartan.** ANCOVA test. Data (means and 95% CI) are delta OSBP adjusted for baseline.

|  | | | | | |
| --- | --- | --- | --- | --- | --- |
| ***Number of patients considered in the model (n)*** | | | | | |
| 6 μg rostafuroxin | 16 |  |  |  |  |
| 50 μg rostafuroxin | 15 |  |  |  |  |
| 500 μg rostafuroxin | 18 |  |  |  |  |
| losartan | 20 |  |  |  |  |
|  | | | | | |
|  | | | | | |
| ***Adjusted means (mmHg)*** |  | **95% CI** | **P** |  |  |
| 6 μg rostafuroxin | -10.8 | -17.48, -4.01 | 0.002 |  |  |
| 50 μg rostafuroxin | -23.0 | -29.91, -16.0 | <0.001 |  |  |
| 500 μg rostafuroxin | -17.4 | -23.74, -11.03 | <0.001 |  |  |
| losartan | -13.2 | -19.22, -7.15 | <0.001 |  |  |
|  | | | | | |
|  | | | | | |
| ***Treatment difference (mmHg)*** | | | | | |
| 6 μg rostafuroxin - 50 μg rostafuroxin | 12.2 | 2.53, 21.89 | 0.014 |  |  |
| 6 μg rostafuroxin - 500 μg rostafuroxin | 6.6 | -2.62, 15.89 | 0.157 |  |  |
| 6 μg rostafuroxin - losartan | 2.4 | -6.61, 11.48 | 0.592 |  |  |
| 50 μg rostafuroxin - 500 μg rostafuroxin | -5.6 | -15.0, 3.85 | 0.242 |  |  |
| 50 μg rostafuroxin - losartan | -9.8 | -18.99, -0.56 | 0.038 |  |  |
| 500 μg rostafuroxin - losartan | -4.2 | -12.98, 4.57 | 0.342 |  |  |
| 50/500 μg rostafuroxin - losartan | -7.0 | -14.65, 0.68 | 0.073 |  |  |
|  | | | | | |

**Table S6. Dropout patients with OSBP level at various time point and dropout reason.** Data are means (mmHg).

| **Patient ID** | **Treatment** | **P2** | ***LSS*** | **Visit 3**  **(mmHg)** | **Visit 4 (mmHg)** | **Visit 5 (mmHg)** | **Reason** |
| --- | --- | --- | --- | --- | --- | --- | --- |
| 10102 | 6 μg rostafuroxin | yes | AA | 146.7 | 149 | 138.7 | patient consent withdrawal |
| 10107 | 6 μg rostafuroxin | no | CA | 155.7 | 145.7 | -- | patient consent withdrawal |
| 10507 | 6 μg rostafuroxin | no | CA | 144.3 | 124 | -- | patient consent withdrawal |
| 10514 | 6 μg rostafuroxin | no | CC | 149 | 172 | -- | hypertension |
| 10720 | 6 μg rostafuroxin | no | CC | 140 | 131 | -- | patient consent withdrawal |
| 10122 | 50 μg rostafuroxin | no | CC | 150.3 | 171.7 | -- | hypertension |
| 10127 | 50 μg rostafuroxin | no | AA | 142.3 | 149.7 | -- | patient consent withdrawal |
| 105233 | 50 μg rostafuroxin | yes | CA | 155 | 128.7 | 126.7 | patient consent withdrawal |
| 10704 | 50 μg rostafuroxin | yes | AA | 144.7 | 174 | -- | interruption, requested by the sponsor |
| 11303 | 50 μg rostafuroxin | yes | CA | 146.3 | 133.7 | 129 | interruption, requested by the sponsor |
| 11309 | 50 μg rostafuroxin | no | CC | 159.7 | 171.7 | -- | interruption, requested by the sponsor |
| 11313 | 50 μg rostafuroxin | yes | CA | 157 | 156.3 | -- | interruption, requested by the sponsor |
| 10118 | 500 μg rostafuroxin | yes | CA | 152.3 | -- | -- | adverse event: skin rash |
| 10401 | 500 μg rostafuroxin | no | CA | 147.3 | 167.7 | -- | hypertension |
| 10542 | 500 μg rostafuroxin | yes | CA | 155.7 | -- | -- | administration of prohibited drugs |
| 11364 | 500 μg rostafuroxin | yes | CA | 149 | 139.3 | 165.3 | hypertension |
| 10508 | losartan | no | CA | 144 | 147.3 | -- | patient consent withdrawal |
| 10517 | losartan | no | CC | 146.7 | 156 | 147 | investigator's decision |
| 10519 | losartan | yes | CA | 145.7 | 111 | 113.7 | patient consent withdrawal |

**Table S7.** **Treatment-Emergent Adverse Drug Reactions (TEADR) by System Organ Class and Preferred Term - Caucasian subgroup and Chinese subgroup.** Data are number (%). An adverse drug reaction is an adverse event judged as suspected to be study drug related.

|  | **6 μg rostafuroxin** | | **50 μg rostafuroxin** | | **500 μg rostafuroxin** | | **losartan** | |
| --- | --- | --- | --- | --- | --- | --- | --- | --- |
| System Organ Class Preferred Term | Number (%) of Patients | Number of Events | Number (%) of Patients | Number of Events | Number (%) of Patients | Number of Events | Number (%) of Patients | Number of Events |
| **Any TEADR - CAUCASIAN** | 0 | 0 | 1 (2.4%) | 8 | 4 (9.3%) | 5 | 1 (2.3%) | 3 |
| Nervous system disorders | 0 | 0 | 1 (2.4%) | 8 | 1 (2.3%) | 1 | 1 (2.3%) | 3 |
| Headache | 0 | 0 | 1 (2.4%) | 8 | 1 (2.3%) | 1 | 1 (2.3%) | 3 |
| Musculoskeletal and connective tissue disorders | 0 | 0 | 0 | 0 | 1 (2.3%) | 2 | 0 | 0 |
| Muscle spasms | 0 | 0 | 0 | 0 | 1 (2.3%) | 2 | 0 | 0 |
| Reproductive system and breast disorders | 0 | 0 | 0 | 0 | 1 (2.3%) | 1 | 0 | 0 |
| Female sexual dysfunction | 0 | 0 | 0 | 0 | 1 (2.3%) | 1 | 0 | 0 |
| Skin and subcutaneous tissue disorders | 0 | 0 | 0 | 0 | 1 (2.3%) | 1 | 0 | 0 |
| Rash | 0 | 0 | 0 | 0 | 1 (2.3%) | 1 | 0 | 0 |
|  |  |  |  |  |  |  |  |  |
| **Any TEADR - CHINESE** | -- | -- | 3 (8.6%) | 4 | 3 (8.8%) | 3 | 2 (5.7%) | 2 |
| Gastrointestinal disorders | -- | -- | 0 |  | 2 (5.9%) | 2 | 0 | 0 |
| Gastrointestinal hypermotility | -- | -- | 0 | 0 | 1 (2.9%) | 1 | 0 | 0 |
| Gastrooesophageal reflux disease | -- | -- | 0 | 0 | 1 (2.9%) | 1 | 0 | 0 |
| Hepatobiliary disorders | -- | -- | 0 | 0 | 1(2.9%) | 1 | 1 (2.9%) | 1 |
| Hepatic function abnormal | -- | -- | 0 | 0 | 1 (2.9%) | 1 | 1 (2.9%) | 1 |
| General disorders and administration site conditions | -- | -- | 1 (2.9%) | 1 | 0 | 0 | 0 | 0 |
| Oedema peripheral | -- | -- | 1 (2.9%) | 1 | 0 | 0 | 0 | 0 |
| Immune system disorders | -- | -- | 0 | 1 | 0 | 0 | 1 (2.9%) | 1 |
| Hypersensitivity | -- | -- | 0 | 0 | 0 | 0 | 1 (2.9%) | 1 |
| Infections and infestations | -- | -- | 1 (2.9%) | 0 | 0 | 0 | 0 | 0 |
| Nasopharyngitis | -- | -- | 1 (2.9%) | 1 | 0 | 0 | 0 | 0 |
| Musculoskeletal and connective tissue disorders | -- | -- | 1 (2.9%) | 1 | 0 | 0 | 0 | 0 |
| Arthralgia | -- | -- | 1 (2.9%) | 1 | 0 | 0 | 0 | 0 |
| Nervous system disorders | -- | -- | 1 (2.9%) | 1 | 0 | 0 | 0 | 0 |
| Dizziness | -- | -- | 1 (2.9%) | 1 | 0 | 0 | 0 | 0 |

**Table S8. Summary of Treatment-Emergent Adverse Events (TEAEs) in Caucasian PEARL-HT.** Adverse events with onset date ≥ date of first randomized study drug intake are presented in this table. SAE, Serious Adverse Event; ADR, Adverse Drug Reaction. Percentages are calculated on the number of patients (N).

|  | **6 μg rostafuroxin N=42** | **50 μg rostafuroxin N=42** | **500 μg rostafuroxin N=43** | **losartan N=43** |
| --- | --- | --- | --- | --- |
| Number of AEs | 11 | 16 | 11 | 16 |
| Number of patients with AEs | 5 (11.9%) | 5 (11.9%) | 7 (16.3%) | 8 (18.6%) |
|  |  |  |  |  |
| Number of SAEs | 0 | 0 | 0 | 0 |
| Number of patients with SAEs | 0 | 0 | 0 | 0 |
|  |  |  |  |  |
| Number of ADRs | 0 | 8 | 5 | 3 |
| Number of patients with ADRs | 0 | 1 (2.4%) | 4 (9.3%) | 1 (2.3%) |
|  |  |  |  |  |
| Number of AEs leading to discontinuation | 0 | 0 | 2 | 0 |
| Number of patients with AEs leading to discontinuation | 0 | 0 | 2 (4.7%) | 0 |
|  |  |  |  |  |
| Number of AEs leading to death | 0 | 0 | 0 | 0 |
| Number of patients with AEs leading to death | 0 | 0 | 0 | 0 |

**Table S9. Individual and mean OSBP values (mmHg) at baseline and after five weeks treatment for profile P2, and *LSS* AA_CC carriers.** Means are (±SD) detected in OASIS-HT study for rostafuroxin 50 μg [1] and in SOPHIA study for losartan [1,4].

| ***OASIS-HT* Profile2 rostafuroxin 50 μg*** | **OSBP baseline** | **OSBP change at visit 6** | **ODBP baseline** | **ODBP change at visit 6** | ***SOPHIA* Profile2 losartan 50 mg*** | **OSBP baseline** | **OSBP change at visit 6** | **ODBP baseline** | **ODBP change at visit 6** |
| --- | --- | --- | --- | --- | --- | --- | --- | --- | --- |
|  | 156.3 | -38.6 | 96.3 | -13.3 |  | 145.0 | -5.00 | 91.3 | -6.0 |
|  | 148.7 | -19.4 | 91.3 | -10.3 |  | 145.0 | -4.00 | 90.0 | -4.0 |
|  | 153.3 | -16.6 | 93.7 | -2.0 |  | 140.7 | 5.67 | 90.0 | 5.7 |
|  | 147.7 | -15.0 | 83.7 | -5.0 |  | 144.0 | 1.00 | 91.0 | 1.0 |
|  | 148.3 | -14.6 | 98.3 | -8.0 |  | 142.0 | -1.00 | 91.7 | -1.0 |
|  | 156.0 | -8.0 | 94.7 | -2.7 |  | 149.4 | -6.40 | 95.8 | -6.4 |
| n=6 |  |  |  |  |  | 143.0 | -10.00 | 96.7 | -10.0 |
| ***mean*** | 151.7 | **-18.7** | 93.0 | **-6.9** |  | 140.0 | -9.00 | 92.0 | -7.0 |
| ***SD*** | 4.0 | **10.4** | 5.1 | **4.4** |  | 148.0 | -16.00 | 92.0 | -9.0 |
|  |  |  |  |  |  | 161.0 | -33.67 | 99.0 | -33.7 |
|  |  |  |  |  |  | 160.0 | -34.00 | 95.0 | -34.0 |
|  |  |  |  |  |  | 147.0 | -13.67 | 95.7 | -15.7 |
|  |  |  |  |  |  | 143.2 | -11.87 | 96.7 | -11.9 |
|  |  |  |  |  | n=13 |  |  |  |  |
|  |  |  |  |  | ***mean*** | 146.8 | **-10.6** | 93.6 | **-10.2** |
|  |  |  |  |  | ***SD*** | 6.7 | **11.9** | 3.0 | **11.9** |
|  |  |  |  |  |  |  |  |  |  |
| ***OASIS-HT* LSS rostafuroxin 50 μg*** | **OSBP baseline** | **OSBP change at visit 6** | **ODBP baseline** | **ODBP change at visit 6** | ***SOPHIA****  ***LSS***  ***losartan 50 mg*** | **OSBP baseline** | **OSBP change at visit 6** | **ODBP baseline** | **ODBP change at visit 6** |
| AA | 156.3 | -38.6 | 96.3 | -13.3 | AA | 162.0 | -32.7 | 101.3 | -16.6 |
| AA | 147.0 | -14.7 | 94.3 | -6.3 | AA | 143.7 | -22.0 | 100 | -22.0 |
| AA | 147.7 | -15.0 | 83.7 | -5 | AA | 151.0 | -21.0 | 96 | -21.0 |
| AA | 148.7 | -19.4 | 91.3 | -10.3 | AA | 149.3 | -8.0 | 94 | -8.0 |
| n=4 |  |  |  |  | AA | 142.1 | -6.1 | 99.7 | -6.7 |
| ***mean*** | 149.9 | **-21.9** | 91.4 | **-8.7** | AA | 145.0 | -4.0 | 90 | -4.0 |
| ***SD*** | 4.3 | **11.3** | 5.5 | **3.8** | AA | 143.7 | -3.7 | 97.7 | -3.7 |
|  |  |  |  |  | AA | 144.0 | -3.0 | 99.7 | -3.7 |
|  |  |  |  |  | AA | 144.0 | 2.3 | 90.7 | 2.3 |
|  |  |  |  |  | n=9 |  |  |  |  |
|  |  |  |  |  | ***mean*** | 147.2 | **-10.9** | 96.6 | **-9.3** |
|  |  |  |  |  | ***SD*** | 6.3 | **11.6** | 4.2 | **8.6** |
|  |  |  |  |  |  |  |  |  |  |
| CC | 149.3 | -9.3 | 78.3 | -5.3 | CC | 146.7 | -34.7 | 94.3 | -19.3 |
| CC | 153.7 | -14.0 | 101.7 | -8.0 | CC | 161.0 | -33.7 | 99.0 | -33.7 |
| CC | 157.0 | -4.0 | 98.7 | -4.0 | CC | 147.3 | -29.3 | 91.3 | -10.0 |
| CC | 165.3 | -7.6 | 95.7 | 2.0 | CC | 148.3 | -23.3 | 106.7 | -23.3 |
| CC | 152.7 | 20.0 | 87.3 | 6.4 | CC | 146.7 | -19.4 | 93.7 | -19.4 |
| CC | 145.0 | -21.0 | 82.7 | -4.4 | CC | 147.3 | -16.6 | 91.3 | -16.6 |
| CC | 163.0 | 3.3 | 91.7 | 9.0 | CC | 148.0 | -16.0 | 92.0 | -9.0 |
| CC | 142.3 | 6.7 | 91.7 | 1.0 | CC | 144.0 | -16.0 | 94.0 | -14.0 |
| CC | 141.7 | 8.0 | 98 | 1.0 | CC | 148.7 | -14.0 | 94.7 | -14.0 |
| CC | 165.7 | -0.4 | 103.3 | -3.3 | CC | 157.0 | -14.0 | 95.0 | -14.0 |
| CC | 142.3 | -1.3 | 92 | 2.0 | CC | 147.0 | -13.7 | 95.7 | -15.7 |
| CC | 143.0 | 14.3 | 84 | -4.7 | CC | 151.3 | -13.0 | 96.7 | -13.0 |
| CC | 142.7 | 14.3 | 93.7 | -1.7 | CC | 157.0 | -13.0 | 95.0 | -10.0 |
| n=13 |  |  |  |  | CC | 147.3 | -12.7 | 97.3 | -14.0 |
| ***mean*** | 151.1 | **0.7** | 92.2 | **-0.8** | CC | 148.4 | -11.0 | 98.4 | -11.0 |
| ***SD*** | 9.2 | **12.0** | 7.5 | **4.9** | CC | 150.0 | -10.3 | 99.3 | -10.3 |
|  |  |  |  |  | CC | 142.7 | -10.0 | 98.7 | -18.7 |
|  |  |  |  |  | CC | 140.0 | -10.0 | 90.0 | -7.3 |
|  |  |  |  |  | CC | 142.7 | -9.7 | 93.4 | -9.7 |
|  |  |  |  |  | CC | 141.7 | -9.7 | 103.3 | -24.0 |
|  |  |  |  |  | CC | 148.0 | -9.3 | 100.3 | -9.3 |
|  |  |  |  |  | CC | 142.3 | -9.3 | 98.0 | -9.3 |
|  |  |  |  |  | CC | 140.0 | -9.0 | 92.0 | -7.0 |
|  |  |  |  |  | CC | 146.0 | -6.0 | 96.7 | -6.0 |
|  |  |  |  |  | CC | 142.0 | -4.7 | 96.0 | -14.7 |
|  |  |  |  |  | CC | 146.3 | -2.3 | 99.3 | -2.3 |
|  |  |  |  |  | CC | 144.0 | 9.7 | 102.0 | -4.7 |
|  |  |  |  |  | n=27 |  |  |  |  |
|  |  |  |  |  | ***mean*** | 147.1 | **-13.4** | 96.4 | **-13.3** |
|  |  |  |  |  | ***SD*** | 5.0 | **9.2** | 3.9 | **6.7** |

*5 weeks for OASIS-HT study; 4 weeks for SOPHIA

**Table S10.** **Baseline and adjusted SBP change from baseline after 5 (OASIS-HT) or 9 (PEARL-HT) weeks of treatment in combined OASIS-HT/PEARL-HT studies.** Baseline means are (±SD) detected in OASIS-HT study for rostafuroxin 50 μg [1], and in SOPHIA study for losartan [1,4]. For OSBP changes data are adjusted for baseline (means and 95% CI).

| **OASIS-HT + PEARL-HT *** | | **rostafuroxin 50 μg** | **losartan** | **OSBP difference rostafuroxin_losartan** | ***P* value** |
| --- | --- | --- | --- | --- | --- |
|  | ***n*** | *23* | *36* |  |  |
|  | ***baseline SBP*** | 151.5 | 149.2 |  |  |
|  | ***SD*** | 6.5 | 8 |  |  |
|  |  |  |  |  |  |
| **Profile P2** | ***delta SBP* (adjusted mean)*** | -20.1 | -13 | -7.1 |  |
|  | ***95% CI*** | -25.29, -14.97 | -17.09, -8.86 |  | 0.035 |
|  |  |  |  |  |  |
|  | ***n*** | *10* | *15* |  |  |
|  | ***baseline SBP*** | 152.1 | 150.1 |  |  |
|  | ***SD*** | 5.1 | 7.5 |  |  |
|  |  |  |  |  |  |
| ***LSS* AA** | ***delta SBP* (adjusted mean)*** | -23.4 | -13.3 | -10.1 |  |
|  | ***95% CI*** | -28.68,-18.11 | -17.59, -8.98 |  | 0.006 |
|  |  |  |  |  |  |
|  | ***n*** | *22* | *45* |  |  |
|  | ***baseline SBP*** | 151.4 | 148.4 |  |  |
|  | ***SD*** | 8.6 | 5.7 |  |  |
|  |  |  |  |  |  |
| ***LSS* CC** | ***delta SBP* (adjusted mean)*** | -2.8 | -15.6 | 12.8 |  |
|  | ***95% CI*** | -7.82, 2.15 | -19.02, -12.10 |  | 0.0001 |
|  |  |  |  |  | 0.00001 (*LSS**treatment) |

* 5 weeks for OASIS-HT study; 9 weeks for PEARL-HT

**Table S11. Office DBP baseline values and changes based on treatment in carriers of P1 and P2 for Caucasian (IT) and Chinese (TW).** Delta means unadjusted for the baseline DBP values. Data from ANOVA are means ± SD. --, not determined. P1 IT, * *P*=0.003; ** *P*<0.001. P2 IT, # *P*=0.018; ## *P*=0.002; ### *P*<0.001; P1 TW, § *P*>0.5; §§ *P*<0.001; P2 TW, @ *P*>0.5; @@ *P*=0.004.

|  |  | **6 μg**  **rostafuroxin** | |  | **50 μg**  **rostafuroxin** | |  | **500 μg**  **rostafuroxin** | |  | **losartan** | |
| --- | --- | --- | --- | --- | --- | --- | --- | --- | --- | --- | --- | --- |
| **ODBP** |  | **ODBP baseline (mmHg)** | **ODBP change**  **after two months (mmHg)** |  | **ODBP baseline (mmHg)** | **ODBP change**  **after two months (mmHg)** |  | **ODBP baseline (mmHg)** | **ODBP change**  **after two months (mmHg)** |  | **ODBP baseline (mmHg)** | **ODBP change**  **after two months (mmHg)** |
|  |  | **P1** | |  | **P1** | |  | **P1** | |  | **P1** | |
| **IT** | **mean** | 91.9 | -4.0 * |  | 92.3 | -9.2 ** |  | 92.4 | -8.7 ** |  | 93.5 | -10.3 ** |
| **n=150** | **SD** | 3.8 | 7.4 |  | 4.2 | 8.6 |  | 3.6 | 9.1 |  | 3.8 | 9.5 |
|  |  | **n=36** |  |  | **n=35** |  |  | **n=39** |  |  | **n=40** |  |
|  |  |  |  |  |  |  |  |  |  |  |  |  |
| **TW** | **mean** | *--* | *--* |  | 95.4 | -0.2 § |  | 95.2 | -0.6 § |  | 93.9 | -6.9 §§ |
| **n=96** | **SD** | *--* | *--* |  | 4.5 | 5.6 |  | 4.3 | 6.2 |  | 4.5 | 8.1 |
|  |  |  |  |  | **n=30** |  |  | **n=33** |  |  | **n=33** |  |
|  |  |  |  |  |  |  |  |  |  |  |  |  |
|  |  | **P2** | |  | **P2** | |  | **P2** | |  | **P2** | |
| **IT** | **mean** | 91.7 | -4.1 # |  | 92.5 | -9.8 ## |  | 92.0 | -10.3 ### |  | 92.8 | -8.2 ### |
| **n=79** | **SD** | 3.8 | 7.1 |  | 4.7 | 10.8 |  | 3.2 | 7.2 |  | 4.3 | 8.5 |
|  |  | **n=20** |  |  | **n=17** |  |  | **n=19** |  |  | **n=23** |  |
|  |  |  |  |  |  |  |  |  |  |  |  |  |
| **TW** | **mean** | *--* | *--* |  | 96.3 | 0.8 @ |  | 94.1 | -0.5 @ |  | 95.2 | -7.5 @@ |
| **n=45** | **SD** | *--* | *--* |  | 3.7 | 5.2 |  | 4.8 | 6.7 |  | 4.0 | 8.9 |
|  |  |  |  |  | **n=14** |  |  | **n=15** |  |  | **n=16** |  |

--, not determined.

**Supporting information**

**Text S1.** PEARL-HT Protocol.

**Text S2.** PEARL-HT Statistical Analysis Plan (SAP).

**Text S3.** PEARL-HT Data Review Report (DRR).

**References**

1. Lanzani C, Citterio L, Glorioso N, Manunta P, Tripodi G, Salvi E, et al. Adducin- and ouabain-related gene variants predict the antihypertensive activity of rostafuroxin, part 2: clinical studies. Science Translational Medicine. 2010;2:59ra87–7.

2. Herrmann SM, Textor SC. Current Concepts in the Treatment of Renovascular Hypertension. Am J Hypertens. 2018;31:139-149.

3. Williams TA, Lenders JWM, Mulatero P, Burrello J, Rottenkolber M, Adolf C, et al. Outcomes after adrenalectomy for unilateral primary aldosteronism: an international consensus on outcome measures and analysis of remission rates in an international cohort. Lancet Diabetes Endocrinol. 2017;5:689-699.

4. Frau F, Zaninello R, Salvi E, Ortu MF, Braga D, Velayutham D, et al. Genome-wide association study identifies CAMKID variants involved in blood pressure response to losartan: the SOPHIA study. Pharmacogenomics. 2014;15:1643-52.

5. Kats MH. Multivariable Analysis. A Practical Guide for Clinicians. Cambridge University Press. 2006,www.cambridge.org/9780521840514.

6. Warnock DG. The amiloride-sensitive endothelial sodium channel and vascular tone. Hypertension. 2013;61:952-4.

7. Brown JJ, Davies DL, Ferriss JB, Fraser R, Haywood E, Lever AF, et al. Comparison of surgery and prolonged spironolactone therapy in patients with hypertension, aldosterone excess, and low plasma renin. Br Med J. 1972;2:729-34.

8. Manunta P, Ferrandi M, Cusi D, Staessen J, Bianchi G. Personalized Therapy of Hypertension: the Past and the Future. Curr Hypertens Rep. 2016;18:24–11.

9. Manunta P, Maillard M, Tantardini C, Simonini M, Lanzani C, Citterio L, et al. Relationships among endogenous ouabain, alpha-adducin polymorphisms and renal sodium handling in primary hypertension. Journal of Hypertension. 2008;26:914–20.

10. Ferrandi M, Molinari I, Torielli L, Padoani G, Salardi S, Rastaldi MP, et al. Adducin- and Ouabain-Related Gene Variants Predict the Antihypertensive Activity of Rostafuroxin, Part 1: Experimental Studies. Science Translational Medicine. 2010;2:59ra86–6.

11. Citterio L, Lanzani C, Manunta P, Bianchi G. Genetics of primary hypertension: The clinical impact of adducin polymorphisms. Biochimica et Biophysica Acta (BBA) - Molecular Basis of Disease. 2010;1802:1285–98.

12. Muxfeldt ES, Fiszman R, de Souza F, Viegas B, Oliveira FC, Salles GF. Appropriate time interval to repeat ambulatory blood pressure monitoring in patients with white-coat resistant hypertension. Hypertension. 2012;59:384-9.

13. Hansen TW, Li Y, Boggia J, Thijs L, Richart T, Staessen JA. Predictive role of the nighttime blood pressure. Hypertension. 2011;57:3-10.

14. Parati G, Stergiou G, O'Brien E, Asmar R, Beilin L, Bilo G, et al. European Society of Hypertension Working Group on Blood Pressure Monitoring and Cardiovascular Variability. J Hypertens. 2014;32:1359-66.

15. Stergiou GS, Ntineri A, Kollias A. Defining Ambulatory Blood Pressure Thresholds for Decision Making in Hypertension: The Effect of Race and Methodology. Circulation. 2017;135:2481-2484.

16. Hinderliter AL, Voora RA, Viera AJ. Implementing ABPM into Clinical Practice. Curr Hypertens Rep. 2018;20:5.

17. Ferrandi M, Manunta P, Balzan S, Hamlyn JM, Bianchi G, Ferrari P. Ouabain-like factor quantification in mammalian tissues and plasma: comparison of two independent assays. Hypertension. 1997;30:886-896.
