# Supplementary material for: Antihypertensive treatment guided by genetics: PEARL-HT, the randomized proof-of-concept trial comparing rostafuroxin with losartan
Source: Pharmacogenomics J. 2021 Mar 1;21(3):346–58. doi: 10.1038/s41397-021-00214-y (PMC8159753; doi:10.1038/s41397-021-00214-y)
Supplement: Supplementary file 3 — Text S1 [file 41397_2021_214_MOESM3_ESM.pdf]

CVie Therapeutics Company Limited.
Unit110-111, Bio-informatics center,
No2. Science Park West Avenue,
Hong Kong Science Park, Shatin, N.T. HK –

Project No.:	CVT-CV-001

PROTOCOL No. Italy: PST 2238-DM-10-001

PROTOCOL No. Taiwan: CVT-CV-001

Version number: CVT-CV-001, 11 July 2017

(Include protocol PST 2238-DM-10-001 Final Version No. 3.0- 7 October 2010, Amendment N.1 - 25 October 2012, Amendment CVT-CV-001 IT3 27 March 2013 ; Amendment CVT CV-001 IT6 30 January 2014, Amendment CVT CV-001 IT6 v.2 27 May 2014, Amendment N.2.1-25 June 2015 and Amendment N.3 V.1 11 July 2017)

ANTIHYPERTENSIVE EFFECT OF DIFFERENT DOSES OF ROSTAFUROXIN IN COMPARISON WITH LOSARTAN, ASSESSED BY OFFICE AND AMBULATORY BLOOD PRESSURE MONITORING IN A HYPERTENSIVE POPULATION SELECTED ACCORDING TO A SPECIFIC GENETIC PROFILE

EudraCT Number 2010-022073-34

C O N F I D E N T I A L

Approved and Signed by: **Date**

Dr. ______________________________ ___/___/20___
Giuseppe Bianchi, MD (dd/mm/yy)
CVie Therapeutics Company Limited
Appointed Medical Expert

Dr. ______________________________ ___/___/20___
Marco Pannacci **(dd/mm/yy)**
CROS-NT.
Appointed Biostatistician

Dr. ______________________________ ___/___/20___
JIANG Su (dd/mm/yy)
CVie Therapeutics Company Limited
Appointed Clinical Quality Assurance

Dr. ______________________________ ___/___/20___
DONG Nathalie (dd/mm/yy)
CVie Therapeutics Company Limited
Pharmacovigilance Officer

______________________________ ___/___/20___
Dr. **Jan A. Staessen, MD, PhD** (dd/mm/yy)
University of Leuven
Chair of the Steering Committee

______________________________ ___/___/20___
Dr. **Chern-En Chiang, MD, PhD** (dd/mm/yy)
Taipei Veterans General Hospital
Co- Chair of the Steering Committee and Responsible for Taiwan

PROTOCOL APPROVAL BY THE PRINCIPAL INVESTIGATOR

I, the undersigned ____
Last Name *First Name*

Principal Investigator at the following site:

SITE ____

declares:

- to have read carefully and thoroughly the Study Protocol:

  CODE: PST2238-DM-10-001 for Italy; CVT- CV-001 for Taiwan

  TITLE: Antihypertensive effect of different doses of **ROSTAFUROXIN** in comparison with Losartan, assessed by office and ambulatory blood pressure monitoring in a hypertensive population selected according to a specific genetic profile.

  VERSION: CVT-CV-001, 11 July 2017

  Approved by the Sponsor: **CVie Therapeutic Company Limited,**
  and
  by the Chair of Steering Committee: **Dr Jan A. Staessen**

- to agree to the above-mentioned Protocol
- to conduct the Study in accordance with Study Protocol and in agreement with the Sponsor's and the Study Chairman's requests.

Signing this document I declare to have read the paragraph relevant to Study acknowledgement and confidentiality and authorise **CVie Therapeutics Company Limited**, to record my data on a computerised archive containing all the data pertinent to the Study.

Signature ______________________________ Date ____/____/20____
dd mm yy

TABLE OF CONTENTS

1.	**GENERAL INFORMATION**	8
**2.**	**STUDY SYNOPSIS**	10
**3.**	**INTRODUCTION**	20
3.1	Basic Information	20
3.2	**ROSTAFUROXIN** Description	23
3.3	**ROSTAFUROXIN** Pharmacology	23
**4.**	**TRIAL OBJECTIVES AND PURPOSES**	28
**5**.	**EXPERIMENTAL DESIGN**	29
5.1	Study Endpoints	30
5.2	Study Plan	30
5.3	Study Diagram	35
**6.**	**STUDY POPULATION**	36
6.1	Sample Size	36
6.2	Parameters Identifying Study Population	36
6.3	Patients Selection	36
6.3.1	Study selection criteria	36
6.4	Removing Patients from the Study	37
**7**.	**TREATMENT OF PATIENTS**	38
7.1	Lifestyle Recommendations	38
7.2	Study Treatments	38
7.2.1	Dosage, Posology and Administration	39
7.2.2	Composition of Used Treatments	39
7.2.3	Packaging and Labelling	40
7.2.4	Drug Accountability	41
7.3	Patients' Numbering	41
7.4	Randomisation	41
7.5	Prior and Concomitant Therapies	42
7.6	Compliance	43
7.7	Treatment Suspension/Discontinuation	43
**8**.	**ASSESSMENT OF EFFICACY**	43
8.1	Primary Efficacy Parameter	43
8.1.1	Clinic (Office) Blood Pressure (OBP) Measurement	43
8.2	Secondary Efficacy Parameters	44
8.2.1	Clinic (Office) sitting Diastolic Arterial Blood Pressure (DBP)	44
8.2.2	24 hours Systolic and Diastolic Blood Pressure Monitoring	44
**9**.	**BLOOD SAMPLING**	45
9.1	DNA management	45
9.2	Endogenous Ouabain management	46
**10**.	**ASSESSMENT OF SAFETY AND TOLERABILITY**	46
10.1	Safety and Tolerability Parameters	46
10.1.1	Heart Rate (HR)	47
10.1.2	Electrocardiogram (ECG)	47
10.1.3	Laboratory Evaluation	47
10.1.4	Adverse Events (AE) and Serious Adverse Events (SAE)	48

10.1.5	Clinical Laboratory Abnormalities and Other Abnormal Assessments as AEs and SAEs	50
10.1.6	Monitoring of AEs	50
10.1.7	Reporting AEs/SAEs	50
10.1.8	Expeditable Events	51
10.1.9	Overdose	51
**11.**	**DIRECT ACCESS TO ORIGINAL DOCUMENTS**	51
**12.**	**QUALITY CONTROL AND QUALITY ASSURANCE PROCEDURES**	52
12.1	Steering Committee	52
12.2	Data and Safety Monitoring Board (DSMB)	53
12.3	Case Report Form (CRF)	53
12.4	Clinical Monitoring	53
12.5	Audits	54
12.6	Inspections	54
**13.**	**DATA MANAGEMENT**	54
**14.**	**STATISTICAL METHODS**	55
14.1	Sample Size Calculation	55
14.2	Patient Population to be Analysed	56
14.3	Statistical Analysis	57
14.3.1	Efficacy Primary End-Point	57
14.3.2	Efficacy Secondary End-Points	58
14.3.3	Safety Analysis	58
**15.**	**ETHICS**	59
15.1	Ethical and Regulatory Authorisations	59
15.2	Informed Consent	59
**16.**	**ADMINISTRATIVE PROCEDURES**	59
16.1	Changes in the Conduct of the Study or Planned Analyses	59
16.2	Suspension/Interruption of the Study	60
16.3	Archiving	60
16.4	Use of the Information of the Publication of the Results	60
16.5	Insurance Covering Public Liabilility	61
16.6	Financial Aspects	61
**17.**	**INVESTIGATOR'S RESPONSIBILITY**	61
**18.**	**FINAL STUDY REPORT**	62
**19.**	**REFERENCES**	63
Appendix I:	**DECLARATION OF HELSINKI (LINK)**	72

LIST OF ABBREVIATIONS AND DEFINITION OF TERMS

ACE	Angiotensin Converting Enzime
AEs	Adverse Events
AM	Antimeridian
AR/SAR	Adverse Reaction/Severe Adverse Reaction
CEU	Caucasian
CH	Chinese
CRF	Case Report Form
CRO	Contract research Organization
DBP	Diastolic Blood Pressure
DNA	Deoxyribonucleic Acid
DOCA	Deoxycorticosterone acetate
DSMB	Data Sefety Monitoring Board
DSU	Drug Safety Unit
ECG	Electrocardiogram
eCRF	Electonic Case Report Form
EO	Endogenous Ouabain
GCP	Good Clinical Practice
γ-GT	Gamma-GlutamylTransferase
h	hour
HR	Heart Rate
IC_{50}	Inhibent Concentration 50%
ICH	International Conference on Harmonisation
IDHU	Investigational Drug Handling Unit
IEC	Independent Ethics Committee
ITT	Intention-To-Treat analysis
IWRS	Interactive Web-based Response System
K	Potassium
kg	kilogram
LD_{50}	Letal Dose 50%
LDL	Low Density Lipoproteins
LOCF	Last Observation Carried Forward
MedDRA	Medical Dictionary for Regulatory Activities
mg	milligram
min	minutes
ml	millilitre
mmHg	millimetres of mercury
mmol	millimoleslitres
MHS	Milan Hypertensive Strain
μg	microgram
n	number
Na	Sodium
OBP	Office Blood Pressure
PM	Postmeridian
PP	Protocol Population
PST2238	(Study Drug)
RAAS	Renin-Angiotensin-Aldosterone System
RNA	Ribonucleid Acid
RPM	Revolutions per Minute
SBP	Systolic Blood Pressure

SAEs	Serious Adverse Events
SGOT/AST	Serum Glutamic Oxaloacetic Transaminase
SGPT/ALT	Serum Glutamic Pyruvic Transaminase
SOPs	Standard Operating Procedures
WHO	World Health Organisation

1. GENERAL INFORMATION

**Protocol no.:**	PST2238-DM-10-001 for Italy, CVT-CV-001 for Taiwan
**Project no.:**	CVT-CV-001
**Title:**	Antihypertensive effect of different doses of **ROSTAFUROXIN** in comparison with Losartan, assessed by office and ambulatory blood pressure monitoring in a hypertensive population selected according to a specific genetic profile.
**Date and Version**	CVT-CV-001 25 Jun 2015 (Include protocol PST 2238-DM-10-001 Final Version No. 3.0- 7 October 2010, Amendment N.1 - 25 October 2012, Amendment CVT-CV-001 IT3 27 March 2013,Amendment CVT-CV-001 IT6 30 January 2014 Amendment CVT-CV-001 IT6 v2 27 May 2014, Amendment N.2.1 -25 Jun 2015 and Amendment N.3 -11 July 2017)
**Sponsor:**	**CVie Therapeutics Company Limited**<br>Unit110-111, Bio-informatics center, No2. Science Park Wes Avenue, Hong Kong Science Park, Shatin, N.T. HK Group
**Product:**	PST 2238
**Pharmaceutical form:**	Capsules
**Dosage:**	6 - 50 - 500 micrograms capsules
**Control Drug:**	Losartan 50 mg
**Pharmaceutical form:**	Tablets
**Chair of the Steering Committee:**	**Jan A. Staessen, MD, PhD**<br>**University of Leuven**
Co Chair of the Steering Committee:	**Chern-En Chiang, MD, PhD**
**Sponsor's Medical Expert:**	**Giuseppe Bianchi, MD**<br>**Cvie Therapeutics Company Limited**<br>Tel +39 348 8545560 +39 02 745569<br>p.zza Adigrat 4, 20133 Milan, ITALY
**Laboratory for Genotype Analysis**	Division of Nephrology and Hypertension<br>University of Milan - San Raffaele Hospital<br>Via Olgettina, 60 20132 Milan – ITALY

**Laboratory for Endogenous Ouabain:**	**Italy:** Division of Nephrology and Hypertension<br>University of Milan - San Raffaele Hospital,<br>Via Olgettina, 60 20132 Milan – ITALY
	**Taiwan:** Ruijin Hospital Affiliated to The Shanghai<br>Jiao Tong University Medical School<br>No.197, Rui Jin Er Road, Shanghai<br>200025 CHINA
**Laboratory for 24 hours Ambulatory Blood Pressure Analysis: Analysis**	Division of Nephrology and Hypertension<br>University of Milan - San Raffaele Hospital<br>Via Olgettina, 60 20132 Milan – ITALY

2. STUDY SYNOPSIS

**Title**	Antihypertensive effect of different doses of Rostafuroxin in comparison with Losartan, assessed by office and ambulatory blood pressure monitoring in a hypertensive population selected according to a specific genetic profile.
**Protocol No.**	PST2238-DM-10-001 for Italy, CVT-CV-001 for Taiwan
**Phase**	II
**Objectives**	***The two primary objectives are as follows:***<br>to demonstrate that the highest two doses of Rostafuroxin are able to show a statistically significant difference on reduction of office sitting systolic blood pressure in comparison to the group of patients treated with Losartan 50 mg in either the total population of CEU Italian and CH Taiwanese patients carrying at least one of the combinations of genotypes included in the **Genetic Profile 2**: or in the subset of patients of this total population carrying only at least one of the combinations of genotypes included in the **Genetic Profile 1**.<br><br>***The secondary objectives are as follows:***<br>According to the clinic blood pressure evaluations:<br>• to demonstrate that the two highest doses of Rostafuroxin are able to show a statistically significant difference on reduction of office sitting systolic blood pressure in comparison to the group of patients treated with Losartan 50 mg in the subset of patients carrying the **Genetic Profile 3**;<br>• to demonstrate that the two highest doses of Rostafuroxin are able to show a statistically significant difference on office sitting diastolic blood pressure in comparison to Losartan in the general population and/or in the two pre-defined subsets of patients; to compare the two highest doses of Rostafuroxin to each other and to Losartan in the CEU Italian and CH Taiwanese sub-population and in the total (CEU and CH) population;<br>• to compare the three doses of Rostafuroxin with each other and versus Losartan in the CEU Italian sub-population;<br>• to determine a dose/responder profile, if any, separately in the two subpopulations and in the total population.<br><br>According to the 24h-Ambulatory Blood Pressure Monitoring:<br>• to identify the oral doses of Rostafuroxin which lead to statistically significant differences in daytime, night-time and overall 24-hour ambulatory systolic blood pressure and/or diastolic blood pressure, in comparison with Losartan in the general population and/or in the two pre-defined subgroups; this analysis will be first run with the two highest doses of Rostafuroxin pulled together versus Losartan and then each single dose of Rostafuroxin will be compared vs. Losartan.

	Referring to the safety and tolerability:<br>• to determine the safety profile of the three doses of Rostafuroxin.
**Country**	Italy and Taiwan
**Total Number of Sites**	About 32
**Study Duration**	The Study will last 15 months. For each patient, the Study will last approximately 15 weeks and will be structured as follows:<br>A Screening Visit, a Run-In Period of a minimum of 15 days and a maximum of 42 days and a Treatment Period of 9 weeks (additional 7 days are allowed in case of late Visit).
**Planned first patient in**	February 2013 (Expected date)
**Study Population**	Patients with arterial hypertension, bearers of a specific genetic profile (**Genetic Profile 1**).
**Sample size**	Two hundred and eighty patients are planned to be randomized in this competition Study.<br>Estimate 120 patients CH Taiwanese patients will be randomized into three parallel arms of treatment with 50 mcg Rostafuroxin, 500mcg Rostafuroxin and Losartan in a 1:1:1 ratio. 160 CEU Italian patients will be randomized into four parallel arms of treatment with 6 mcg Rostafuroxin, 50 mcg Rostafuroxin, 500mcg Rostafuroxin and Losartan in a 1:1:1:1 ratio.<br>Approximately 50% of the whole sample is expected to bear a mutation included in the Genetic Profile 2. Therefore, the sample size of each arm in this pre-defined subset of patients bearing the Genetic Profile 2 will be approximately of :<br>40 patients for 50, 500, micrograms of Rostafuroxin and Losartan arms, and 20 CEU Italian patients in 6 micrograms of Rostafuroxin arm).<br>Sample size has been computed by assuming a standard deviation of 10 mmHg. The other assumption is that the **Genetic Profile 2** will comprise at least 50% of the total randomized patients.<br>Using these assumptions, a total sample size of 240 patients, i.e. 80 patients in the following groups (Rostafuroxin 50, 500 micrograms and Losartan 50 mg), will give a power of at least 80% for discriminating a SBP mean difference of 4.5 mmHg between the treatments of Rostafuroxin and Losartan, with a standard deviation of 10mmHg, at an alpha level of 0.05, two tailed test, in the PP population and assuming a 10% drop-out rate.<br>A total sample size of 120 patients (50% of the total sample), i.e. 40 patients in each group, is suitable for discriminating a SBP mean difference of 6.5 mmHg between the group of the two highest doses of Rostafuroxin and Losartan, with a standard deviation of 10 mmHg a power of 80%, an alpha level of 0.05, two tailed test, in the PP **Genetic Profile 2** population and assuming a 10% drop-out rate.<br>An interim blinded sample size re-assessment is planned and will be performed when approximately half of the patients (i.e. 140) will be randomized and will reach the final evaluation.

Interim blinded sample size re-assessment results

A sample size re-assessment was performed to confirm the sample size requirement for this trial. The endpoint considered for the standard deviation estimation was the change in the office measurement of sitting blood pressure from baseline (Visit 3) to Visit 6. Missing data was not replaced.

The sample size re-assessment showed a higher variability compared to the assumptions of the Study Protocol. In addition to this, as discussed in detail in the attached rationale, the very fast development of personalized medicine requires the most appropriate translation of the CRT data to the individualized day to day clinical practice. This has prompted the Sponsor to add as a co-primary end point an individual assessment of efficacy which identifies the responders and non-responders in each treatment arm.

Considering the sample size foreseen in the Study Protocol and the percentages of responders seen in previous trials, the table below shows the effect of these assumptions on the power:

Genetic Profile	Total No. of evaluable subjects	Total number of randomised subjects	Alpha Level	Reference proportion	Proportions difference scenarios	Power
1	216	240 (80 per group)	0.025	35%	20%	0.708
					30%	0.978
					40%	>.999
2	105	120 (40 per group)	0.025	30%	20%	0.380
					30%	0.758
					40%	0.963

Considering the actual number of patients who completed the study in Italy, at least 102 patients should be reached in Taiwan to guarantee the levels of power included in the table above.

Study design

This is a Phase II multicenter, double-blind, double-dummy, three-arms, parallel group, active comparator controlled Study. Three oral doses of Rostafuroxin (6- 50 - 500 micrograms) will be studied versus Losartan 50 mg. The Treatment Period will last 9 weeks. The arm of Rostafuroxin 6 mcg shall be carried out in CEU Italian patients, only

Study Product, Dose, Route, Regimen

Rostafuroxin and Losartan 50 mg will be administered in this Study once daily, under double-blind, double-dummy conditions. Each patient will be randomized to one of the following treatment schedules:

- 1 capsule of Rostafuroxin (6 micrograms) plus one tablet of placebo of Losartan, once a day before breakfast; for the active treatment this is the lowest reproducible amount of substance feasible for pharmaceutical manufacturing (GMP) and will be administered only

	at CEU Italian patients;<br>o 1 capsule of Rostafuroxin (50 micrograms) plus one tablet of placebo of Losartan, once a day before breakfast;<br>o 1 capsule of Rostafuroxin (500 micrograms) plus one tablet of placebo of Losartan, once a day before breakfast;<br>o 1 tablet of Losartan 50 mg plus one capsule of placebo of Rostafuroxin, once a day before breakfast.
**Duration of administration**	9 weeks
**Main eligibility criteria**	**Screening entrance condition**<br>In order to be eligible for the Study, patients have to be aged between 25 and 60 years, have already undertaken lifestyle recommendations and still having an abnormal systolic and diastolic blood pressure levels. Furthermore, they must not have been previously treated with any specific antihypertensive drug and they must not assume drugs like diuretics, ß-blocker agents, Ca-antagonist, ACE inhibitors and AT_1-receptor blockers, for other reasons. They must not be on statines treatment or to be Diabetic (fasting plasma glucose > 125 mg/dl).<br>Their value of the sitting systolic blood pressure (SBP) must range between 140 and 169 mmHg and the sitting diastolic blood pressure (DBP) must range between 85 and 100 mmHg, after an adequate period of lifestyle changes.<br>They do not have to present known causes of secondary hypertension, cardiac disease requiring prohibited pharmacological treatment or history of renal artery disease or a myocardial infarction occurred within the last 6 months.<br>All the above-cited evaluations are considered routine investigations to make a correct diagnosis of arterial hypertension and assessment of the patients cardiovascular risk, therefore no specific informed consent is requested. In fact, all these evaluations are usually performed by the Physician any time a patient is presenting with elevated blood pressure levels.<br>Pregnant or nursing women or women of childbearing potential not taking anti-contraceptive medication or not utilizing a double contraception method are not suitable for the Study.<br>Finally, known renal impairment (Estimated Creatinine Clearance $\leq$ 50 mL/min) and obesity ($\geq$ 30 kg/m^2) are conditions excluding patients from the possibility to participate in this Study.<br>If the Clinic Centre Physician or a General Practitioner is identifying, during his/her outpatient routine practice, a patient suitable for the Study, according to the above cited characteristics, he/she will inform the patient about the possibility to participate in such Study and if the patient declares to be interested to obtain more information about the Study, he/she will be referred for a next visit to the Clinic Centre, where a physician will explain to him/her the Study, in full.<br>If the patient has been identified by a General Practitioner, this doctor will

	inform the Clinic Centre of the presence of a suitable patient. The General Practitioner will send to the Clinic Centre a note of the patient medical history and of the tests performed to make the diagnosis of arterial hypertension and the assessment of the patient cardiovascular risk.<br>**Study selection criteria**<br>The Clinic Centre will evaluate the following inclusion and exclusion criteria during the scheduled Screening Visit (Visit 1) and following Run-In Visits (Visits 2 and 3):<br><u>Inclusion Criteria</u><br>• Signature of a written informed consent, included informed consent on genotype analysis.<br>• Male/female patient aged 25-60 years.<br>• Naive hypertensive patient (new diagnosed patient, never treated before or, if treated up to 7 days, after 1 month of washout).<br>• Documented mild to moderate arterial hypertension, as defined below:<br>o At Visits 1 and 2 the mean of the last 3 consecutive readings of SBP must be ≥ 140 mmHg and DBP must be ≥ 85 mmHg, when measured by Clinic (OBP);<br>o At Visit 3, the mean of the last 3 consecutive readings of SBP must be ≥ 140 mmHg ≤ 169 mmHg and DBP must be ≥ 85 mmHg and ≤ 100 mmHg, when measured by Clinic (OBP).<br>• Presence of at least one mutated genotype or combination of genotypes corresponding to the list provided in Genetic Profile 1.<br><br><u>Exclusion Criteria</u><br>• Known causes of secondary hypertension;<br>• Severe or malignant hypertension;<br>• History of renal artery disease;<br>• Significant renal or hepatic disease ;<br>• Cardiac disease requiring prohibited pharmacological treatment or history of myocardial infarction within the last 6 months;<br>• Atrial Fibrillation;<br>• Complete Left and/or Right Ventricle Bundle Branch Block;<br>• First degree AV-block exceeding 240 msec;<br>• Electrocardiographic evidence of left ventricular hypertrophy;<br>• Pregnant or nursing women or women of childbearing potential not taking anti-contraceptive medication or not utilizing a double contraceptive method;<br>• Surgery or disease of the gastrointestinal tract, which might influence absorption or elimination of the drug;<br>• Any concomitant condition that may, in the judgement of the Investigator, jeopardise participant adherence to the Protocol or ability to complete the trial;<br>• Concomitant therapy with medications that may affect blood pressure;

	• Treatment with any investigational drug in the previous 6 months;<br>• Predictable lack of cooperation;<br>• Obesity ≥ 30 kg/m^2;<br>• Diabetes mellitus;<br>• Statines treatment.
**Evaluated Parameters and collection times**	***Screening Visit***<br>All patients identified by General Practitioners, being themselves the first contacts with such patients on the field, or by Clinic Centre Physicians and judged suitable for the Study, will be informed about this Study and if interested in taking part of it, referred to the Investigational Clinic Centre to enter into the Study and to perform a Screening Visit and Run-In Period to verify the inclusion/exclusion criteria.<br>The Screening Visit consists of the following procedures:<br>**Visit 1 - Screening**<br>- patient's informed consents collection;<br>- recording of patient medical/surgical history including concomitant medications and prior medications;<br>- physical examination (including body weight and height measurement);<br>- ECG at rest;<br>- clinic (office) blood pressure and heart rate measurements;<br>- blood sampling for chemistry and haematology (within a time window of + 3 days from the Screening Visit and under fasting condition);<br>- urine sampling for urinalysis (within a time window of + 3 days from the Screening Visit);<br>- blood sampling for measurement of the genotype;<br>- blood pregnancy test to be performed on all women of childbearing potential.<br>- check of verifiable inclusion/exclusion criteria;<br>***Run-In Period:***<br>**Visit 2 – (between Day 14 and Day 42)**<br>(physical examination (including body weight);<br>- check of concomitant medications;<br>- check of concomitant diseases recovery.<br>- clinic (office) blood pressure and heart rate measurements;<br>- start measurement of the 24 hours ambulatory blood pressure;<br>- patient's delivering of the 24 hours urine collection;<br>**Visit 3 - The Day After Visit 2**<br>- stop of the 24 hours ambulatory blood pressure measurements;<br>- check of concomitant medications;<br>- check of concomitant diseases recovery;<br>- clinic (office) blood pressure and heart rate measurements;<br>- check of all inclusion/exclusion criteria;

	- blood sampling for Endogenous Ouabain (EO);<br>- randomization and drug dispensing for the subsequent Study Period;<br>- delivery to the patient of the Patient's diary;<br>- delivery to the patient of a letter for the General Practitioner.<br><br>**Visit 4 - Day 14** (Two weeks after the starting of the Treatment Period; time window allowed for this Visit is: scheduled time + 7 days):<br>- physical examination (including body weight measurement);<br>- clinic (office) blood pressure and heart rate measurement;<br>- check of concomitant medications;<br>- check of concomitant diseases recovery.<br>- recording AEs, if any;<br>**Visit 5 - Day 35** (Five weeks after the starting of the Treatment Period; time window allowed for this Visit is: scheduled time + 7 days):<br>- physical examination (including body weight measurement);<br>- clinic (office) blood pressure and heart rate measurement;<br>- check of concomitant medications;<br>- check of concomitant diseases recovery;<br>- ECG at rest;<br>- recording AEs, if any;<br>- Study Drug accountability relevant to the capsules returned by the patient;<br>- Study Drug dispensing for the subsequent Study Period.<br>**Visit 6 - Day 63** (Nine weeks after the starting of the Treatment Period; time window allowed for this Visit is: scheduled time + 7 days):<br>- physical examination (including body weight measurement);<br>- clinic (office) blood pressure and heart rate measurement;<br>- blood sampling for chemistry and haematology (within a time window of - 3 days from the scheduled Visit and under fasting condition);<br>- blood sampling for EO;<br>- urine sampling for urinalysis (within a time window of - 3 days from the scheduled Visit);<br>- blood pregnancy test to be performed on all women of childbearing potential.<br>- check of concomitant medications;<br>- check of concomitant diseases recovery;<br>- recording AEs, if any;<br>- starting of measurement of the 24 hours ambulatory blood pressure.<br>**Visit 7 - The Day After Visit 6**<br>During this Visit, the following procedures will be performed:<br>- stop of the 24 hours ambulatory blood pressure measurements;<br>- clinic (office) blood pressure and heart rate measurement;<br>- ECG at rest;<br>- check of concomitant medications;

	- check of concomitant diseases recovery;<br>- Study Drug accountability relevant to the capsules returned by the patient;<br>- delivering by the patient of the filled in patient's diary;<br>- recording AEs, if any;<br>- prescription of the standard antihypertensive treatment.
**Concomitant treatments**	*Prohibited drugs:*<br>• concomitant antihypertensive or anti-anginal treatments (such as ACE inhibitors, angiotensin II antagonists, Ca-channel blockers, beta-blocking agents, alpha-methyldopa, prazosin, reserpine and other centrally active antihypertensive drugs), diuretics;<br>• medications causing systemic vasodilation or vasoconstriction such as theophylline, papaverine, trycyclic antidepressants, neuroleptics, long-acting nitrates, sympathicomimetic nasal agents;<br>• anti-arrhythmic agents and digitalis;<br>• non-steroid anti-inflammatory agents (with the exception of aspirin at low doses) for a period longer than 1 week;<br>• chronic use of steroids;<br>• immunosuppressive or cytotoxic agents;<br>• statines.<br>The occasional use of short-acting nitrates will be allowed (on demand).<br>A short treatment period of a maximum of 7 days of the above listed treatments not limit the enrolment of a patient, but it is mandatory a wash - out period of at least 28 days before randomisation.
**Statistical Analysis for Efficacy and Safety**	All statistical analyses will be performed with SAS software (SAS Institute Inc.) version 9.2. The statistical analyses will be performed by the Biostatistics and Data Management Unit of CROS NT and detailed in the statistical analysis plan (SAP) which will be prepared by the same unit.<br><br>The primary efficacy endpoints will be:<br>• Change from baseline to Visit 6 in office sitting SBP.<br>• Proportions of responders at Visit 6, defined as "Patients having the mean office SBP (mean of last three measurements) <= 135 mmHg or having a reduction in mean office SBP (mean of the three last measurements) > or = 10% with respect to the baseline measurement" at Visit 6 (after two months of therapy).<br><br>Secondary efficacy and safety end-points will be:<br>• Change from baseline to Visit 6 in office sitting DBP.<br>• Office sitting DBP and office sitting SBP values and changes from baseline at each study visit.<br>• 24 hours SBP and DBP measurements. 24 hours, Day-time and night-time weighted mean value of both SBP and DBP.<br>• All standard safety endpoints (AEs, vital signs, ECG, laboratory data and physical examination).<br>• Office sitting SBP and DBP corrected as per seasonal variation of

	temperature (confounding factor).<br><br>All variables will be descriptively analysed by Treatment and Visit (mean, standard deviation, minimum and maximum for continuous variables that are normally distributed; median and 25th and 75th percentiles for continuous variables that are asymmetrically distributed; frequency distribution for categorical variables).<br>Efficacy analyses will be applied in both the ITT and the PP populations, while the safety analyses will be carried-out in the Safety population.<br>Results from the PP population will be considered the primary ones.<br><br>Hypotheses concerning the two Genetic profiles will be tested in a hierarchical order for the primary endpoints. The hypotheses (H_1 and H_2) related to the genetic profile 2 will be tested first. The alpha level will be adjusted for taking into account the two co-primary endpoints, linked by the "OR" condition. Therefore, the alpha level will be set to 0.025.<br>The same hypotheses (H_3 and H_4) will be tested in the genetic profile 1 only if at least one of H_1 or H_2 are rejected.<br>Further details of multiplicity adjustment procedure and alpha propagation method will be provided in the SAP.<br>An analysis of covariance (ANCOVA) model will be used for analysing the changes from Baseline to Visit 6. Treatment, country and the interaction between these two will be included as factors, and baseline will be included as a covariate.<br>In order to demonstrate that the two highest doses of **ROSTAFUROXIN** are able to show a statistically significant difference on reduction of office sitting systolic blood pressure in comparison to the group of patients treated with Losartan 50 mg, the appropriate contrast will be carried-out within this ANCOVA model. This analysis will be performed in the total population bearing a mutation included in the **Genetic Profile 1** and the subgroup of patients showing the **Genetic Profile 2**.<br><br>The Safety population will be used to evaluate safety and tolerability data.<br>Physical examinations, ECGs, vital signs, laboratory tests, adverse events and concomitant medications will be considered for the safety and tolerability evaluation.<br>Categorical variables (ECG normality, physical examination normality, etc.) will be analysed with shift tables (Baseline vs. final Visit), while continuous variables (laboratory parameters, vital signs, etc.) by descriptive summaries.<br>Laboratory data will also be analysed with shift tables (Baseline vs. Final Visit), considering each value as being normal/abnormal with respect to the appropriate normal ranges. Normal-abnormal shift tables will also be presented for physical examination results.<br>In order to facilitate identification of outliers and medical interpretation of results, normalised laboratory values will be graphically represented by

	means of plots of Baseline vs. Final Assessments.<br>Adverse events will be coded using the MedDRA dictionary. Adverse events and all related information will be listed by patient. Descriptive statistics will be performed stratifying the events by system organ class and preferred term; they will also be stratified by seriousness and relationship with the Study Treatment.<br>Prior and concomitant medications will be summarised using the WHO coding system. Prior and concurrent medical conditions will be coded using the MedDRA dictionary.

3. INTRODUCTION

3.1 Basic Information

About 30% of the world adult population is affected by hypertension in industrialised countries. Elevated arterial pressure is the major cause of cardiovascular mortality[1,2] and international guidelines emphasise the benefits of reducing blood pressure[3-5]. The current antihypertensive strategies may reduce by 20-30% the cardiovascular risk of hypertensive patients when this efficacy is measured in clinical trials in comparison with placebo[6]. A precise world-wide estimation of this efficacy both in term of patient burden and healthcare costs is not available[7-9]. However, a recent analysis suggests that the world-wide cost of hypertension associated cardiovascular complications is around 1,000 billions dollars[10]. Therefore, effective improvement in the diagnosis and treatment of hypertension can provide the most significant contribution to the decrease of cardiovascular mortality and reduction of the world-wide costs associated to treatment of hypertension complications.

Five classes of antihypertensive agents are recommended for the first-line treatment of hypertension; the newest one is the angiotensin II type 1 (AT1)-receptor blocker class that represents a class of effective and well tolerated orally active antihypertensive drugs. These agents effectively control hypertension when given once daily. The capacity of such drugs in reducing clinical events such as stroke or end-stage renal disease in hypertension has been shown in large clinical trials[11-13]. Losartan was the first available AT_1-receptor blocker and the dose of 50 mg once daily was proved to be effective in reducing arterial blood pressure in adult patients with mild-moderate essential hypertension[14-17].

Most of clinical trials, performed with the aim to show a reduction of the systolic blood pressure in hypertensive patients, show that reduction of systolic blood pressure is independent from the class of tested drugs as diuretics, β blockers, Ca channel blockers or inhibitors of RAS seem to have roughly the same efficacy[6]. These findings have been used as an argument to support the notion that the antihypertensive therapy efficacy in reducing cardiovascular risk depends on the magnitude of the blood pressure fall rather than on the mechanism of action of the drug. This view contrasts with the well established notion that the secondary prevention capacity in other cardiovascular diseases differs among these classes of drugs[18] with minor difference on the prevention of heart failure or stroke between the Ca antagonist and the other classes of drugs.

Furthermore, the recent findings[19-21] on genetic of hypertension taken together with the previous data on pathophysiology of hypertension and its cardiovascular complications are consistent with the notion that a variety of heterogeneous genetic-molecular mechanisms concur to develop the rather uniform clinical picture of primary hypertension. Drugs are small molecules that produce their effects by interacting with larger molecules (proteins) whose function or reactivity may vary from one patient to another because the variations within the gene encoding them. Therefore, it is logical to postulate that the consequence of this different interaction either in term of blood pressure reduction or cardiovascular risk prevention may vary from a patient to another according to the peculiar function of the proteins involved in a given patient.

In trying to understand the mechanisms of reduction of cardiovascular risk displayed by the various drugs in comparison to placebo in thousand of patients we have to consider:

a) the heterogeneity of the genetic-molecular mechanisms underlying primary hypertension with its cardiovascular complication;
b) the heterogeneous mechanism of action of the antihypertensive drug;
c) the similar reduction of cardiovascular risk (around 20-30%) among the various drugs.

A possible way to reconcile these established facts is the following:
a similar reduction of about 20% of cardiovascular risk observed with the different classes of drugs may occur in different subsets of patients for each drug. This may give an apparent similarity in efficacy among the drug that is only due to our inability to match the heterogeneous-molecular mechanisms of hypertension and its cardiovascular complications, with the heterogeneous action mechanisms of these drugs.
This matching may be achieved throughout the pharmacogenomic approach.

Another confounding aspect that must be taken into consideration when designing a clinical trial investigating a new antihypertensive drug is the previous treatment taken by the patients entering into a clinical trial. In almost all these studies the blood pressure response is measured as a difference between the level of blood pressure after a period of the specific treatment (ranging from 15 days to few months) and the baseline blood pressure level measured after an interval of washout from the previous therapy ranging from 1 to 30 days even if it is well recognised that a washout period of one month is considered insufficient to remove the effect of the previous therapy. Various authors(22) suggest that in placebo controlled studies a therapy-free baseline requires a withdrawal of previous therapy for at least eight weeks.
In addition, usually the Study populations of the clinical trials consist of patients with a new onset hypertension and patients with long lasting hypertension. These two populations should not be mixed in clinical trials because outcomes have been demonstrated to be different. Studies in never treated patients have shown that only 30-40% of patients may be considered as responders(23,24). Conversely in most clinical trials carried out in previously treated patients the rate of responders may double (from 60-80% patients). The reasons of this huge difference are not entirely clear but certainly more responders are recruited in clinical trials because these patients are more represented in the cohort of patients followed by the clinical centres contributing to the clinical trials. For instance, due to the very huge use of renin-angiotensin system inhibitors or diuretics, patients that have been responding to these drugs are more likely responders to a new drug that interferes with the renal Na handling or renin-angiotensin system mechanisms. Moreover, the rebound effect caused by the sudden withdrawal of the previous drug, contributes to the rise in blood pressure after withdrawal (for instance the increase of renin-angiotensin system activity after withdrawal of renin-angiotensin system inhibitors or diuretics) that, per se, may favour the effect of a new renin-angiotensin system inhibitor independently from the existing patients' genetic architecture triggering the initial blood pressure rise.
Experimental data have shown that in presence of a known cause of hypertension (for instance experimental induced renal injury) the mechanisms underlying hypertension and, consequently the response to the various classes of drugs differ across the different phases of hypertension(25-31). Very likely the same "phase effect" may also occur in the essential hypertension. For instance the cardiovascular remodelling on the associated secondary kidney functional change occurring when hypertension persists for a certain period of time (that may vary from one patient to another) may, per se, be a factor modulating the blood pressure response(32-34). This phase effect should also be taken into consideration in connections between DNA variations and clinical symptoms or phenotypes; in this respect we should not ignore the lesson from monogenic diseases. In fact carriers of the same culprit allele may have a severe disease at young age or just a mild biochemical abnormality at adult age depending upon the presence of peculiar modifier genes or environmental factors(35,36). This context dependency is

obviously magnified in complex multifactorial diseases. Therefore, any candidate gene allele effect must be assessed by considering its appropriate genetic and environmental context.
The importance of excluding or at least accounting for these confounding factors is clearly demonstrated by the OASIS Study data[37-39]. This Study included both new discovered and never treated patients and patients who followed a period of washout of 1 month from a previous therapy. The predicted genotype-blood pressure response relationship was present only in the first subset of patients[38].
As this prediction regarded genes that were also shown to be involved in cardiovascular complications[40], the definition of the target population by the presence of both a preliminary genetic profile and the response to the drug hitting these genetic mechanisms, may also improve our capacity to prevent cardiovascular complications.
After the demonstration that the candidates genes they hypothesized in 2005[41], successfully predicted the response to **ROSTAFUROXIN**, the analysis was extended to test whether other DNA regions could modulate these candidates gene-blood pressure response relationship. This extension was carried out with whole genome scanning by genotyping 1 million SNPs with the Illumina human 1M array. The DNA variations captured by these SNPs included both genes known to affect many physiological or pathological pathways, previously shown to be involved in causing hypertension and its cardiovascular complications or non-coding DNA regions whose function is largely unknown. The inclusion of these last DNA regions is indispensable since the genetics of complex disease or phenotype (like the response to a given drug) is moving from the classical paradigm: one gene – one mechanism – one clinical symptom (or phenotype) to a more complex paradigm where several factors: genetics (arising from variations within coding or not-coding DNA regimes), biological and environmental interact each others to form a network. In fact variation within non-coding transcripts, Non-coding RNAs or ncRNAs (miRNAs, siRNA, small RNA etc…) is becoming increasingly important in the regulation of gene expression, gene-networks and alternative splicing. Therefore, any Study of the genotype-phenotype relationship must include this comprehensive approach covering the whole spectrum of genetic factors governing the transmission of the information across enkaryotic organisms.

In conclusion, many confounding effects in most of the clinical trials performed in hypertensive patients could result in masking peculiar differences in blood pressure reduction and cardiovascular protection among the different classes of utilized drugs, and if all these considerations will be taken into account in future trials, probably the results on reduction of blood pressure in different groups of patients will not result to be equal for different treatments.
In fact, an historical comparison of the blood pressure fall with **ROSTAFUROXIN** in a specific gene selected population showed that the blood pressure fall was at least 50% greater than that obtained with the administration of Losartan or Hydrochlorothiazide (data on file) [38,40].
This population was affected by mutations in genes encoding for adducin proteins (ADD1, ADD2, ADD3) and the enzymes involved in EO synthesis. Various researchers have identified these two genetic-molecular mechanisms, adducin polymorphisms[41-45] and increase in endogenous Ouabain (EO)[46,47] as potential triggers of arterial hypertension and its cardiovascular complications, throughout their interaction with the Na,K pump function and Na,K-ATPase-SRC signalosome activation.

3.2 ROSTAFUROXIN description

ROSTAFUROXIN is a potent, selective and safe inhibitor of this altered mechanism and has been proved to be effective in reducing blood pressure levels in Adducin mutated rat strains and in chronic Ouabain infused rat model.

ROSTAFUROXIN (17β-(3-furyl)-5β-androstan-3β,14β,17α-triol) is a digitoxygenin derivative[48] able:

- to selectively block at nanomolar concentrations the increase of Na-KATPase produced by "hypertensive" α-adducin allele[49] and low concentrations of Ouabain in cell colture[50];
- to inhibit isolated and purified dog kidney Na-KATPase with an IC_{50} of 2.5 x 10^{-5} M[46,50];
- to reduce blood pressure and to normalize increased renal Na-KATPase activity both in MHS rats[49] and in rats with Ouabain- dependent hypertension[46,50].

ROSTAFUROXIN does not interfere with receptors involved in blood pressure regulation and steroid hormonal homeostasis, up to 10-5M[50], does not affect Na-K pump in normal cells[49-50], neither affects blood pressure and Na-K pump activity in normotensive animals[49,50]. Moreover, **ROSTAFUROXIN** does not behave as a typical diuretic[51] thus not inducing the unwanted side-effects of diuretics (activation of RAAS, alteration of the electrolyte, lipidic and glucidic pathways)[52-55].

ROSTAFUROXIN displays a favourable effect on vascular alterations in animal models of volume-dependent hypertension (DOCA-salt rats)[56]. It improves acethilcoline-dependent vasorelaxation by increasing the nitric-oxide synthesis and bioavailability and reduces the superoxide anion generation without changing Na-KATPase activity in mesenteric arteries from DOCA rats[56].

ROSTAFUROXIN is also effective in preventing the proteinuria associated with podocytopathies and decreased nephrin expression in Adducin mutated rat strains and in chronic Ouabain-infused rat model[57].

3.3 ROSTAFUROXIN Pharmacology

General and safety pharmacology studies indicate that **ROSTAFUROXIN** does not affect cardiac and vessel contractility both "in vitro" and "in vivo"[58,59]. It does not alter urinary composition and creatinine clearance after acute and chronic administration[51]. In particular, it does not induce the unwanted effects of diuretics such as activation of the renin-angiotensin-aldosterone-system and alteration of the lipidic, glucidic and electrolyte profiles[51].

ROSTAFUROXIN does not affect heamodynamics in the anaesthetised dog[60]. It is devoid of direct androgenic, estrogenic and corticomimetic effects "in vivo"[61,62], it does not alter either adrenocorticotropic hormone or prolactin secretion[61,62]; it does not affect either steroidogenesis "in vitro"[63] or gastrointestinal motility "in vivo"[64].

In particular, despite its digitalis-like structure, **ROSTAFUROXIN** does not affect either cardiac contractility or induce pro-arrhythmogenic effects both on isolated heart preparations and "in vivo"[65].

Acute and subchronic toxicology studies have been performed. Acute oral LD_{50} in rat and mouse is > 2000 mg/kg[66,67]. One- and three-month oral subchronic toxicity have been performed in both rat and monkey. One-month repeated administration to rat at 45-180 and 720 mg/kg[68] induced a 60 % of deaths only in male animals at the highest dose; in those animals general signs

of cachexia were evident. In both sexes dose-dependent signs of haematological and hepatic toxicity were present. Three-month repeated oral administration to rat up to 100 mg/kg[(69)], did not induce either mortality or significant changes of hematochemical parameters, except for a slight decrease of serum GOT and an increase in alkaline phosphatase in males. One-month repeated administration to monkey at 45-180 and 720 mg/kg caused neither death nor clinical abnormalities; mild signs of hepatic and haematological toxicity occurred at higher doses[(70)]. Three-month repeated oral administration to monkey up to 180 mg/kg did not cause any toxicological alteration[(71)].

In humans, multiple dose administration of up to 10 mg daily of **ROSTAFUROXIN** (7-days multiple dose) showed excellent tolerability[(72)]. No adverse events occurred in the treated subjects.

In essential hypertensive patients, a clinical trial named “OASIS” trial studied the effects of different doses of **ROSTAFUROXIN**[(37-39)].

OASIS trial was a double-blind controlled dose-finding Study comparing the effect of **ROSTAFUROXIN** with placebo with a crossover design in hypertensive patients[(37,38,41)]. After a four–week Run-In Period without treatment, 435 eligible never or previously treated patients were randomized to placebo or five oral doses of **ROSTAFUROXIN**: 0.05, 0.15, 0.5, 1.5 or 5 mg/day for periods of 5 weeks. The primary end point was the reduction of office systolic blood pressure (SBP) over placebo, while the secondary end point included the dependence of the blood pressure response on the variation of genes encoding for adducin proteins (ADD1, ADD2, ADD3) and the enzymes involved in EO synthesis (LSS, HSD3B1-B2, CYP11A1) or transport (MDR1 and SLC04C1) EO genes[(41)].

Data coming from the general population did not show an effect of **ROSTAFUROXIN** on blood pressure, but analyses performed in the subpopulation bearing some of the above mentioned genetic mutations, chosen “a priori” showed a clear effect in the subset of patients who were never pharmacologically treated before[(37,38)]. The previously discussed confounding effect of previous treatments and short wash-out period from previous therapies could account for the lack of a statistically significant effect when the whole population of patients bearing the gene mutations was investigated.

The magnitude of SBP fall after **ROSTAFUROXIN** administration was modulated by the polymorphisms of the selected genes variants, either when considered alone or in combination.

When single SNP analysis was carried out, LSS rs914247 was the only SNP found to be associated with a statistically significant larger systolic blood pressure fall with all the pulled **ROSTAFUROXIN** doses compared to placebo (model $p = 0.015$ interaction $p = 0.009$) with a blood pressure fall corrected by placebo of 9,5 mmHg in carriers of the mutated homozigous genotype[(37,38)]. The influence of LSS rs914247 variation on **ROSTAFUROXIN** effect was already present at the dose of 0.05 mg (about 2 nmoles/kg), consistently with rat results.

A list of further significant interactions among the carriers of gene variants with the corresponding “effect-size” are reported herewith below:

- LSS rs2254524 AA & ADD1 rs4961 GT+TT
- ADD1 rs4961 GT+TT & ADD3 rs3731566 GG for Caucasian (CEU) and ADD1 rs4961 GT+TT & ADD3 rs2501574TT for Chinese (CH)[(73)]
- LSS rs2254524 CA+AA & MDR1 rs1045642 CC
- MDR1 rs1045642 TT & HSD3B1 rs10923835 AT+TT for CEU and MDR1 rs1045642 TT & HSD3B1 rs117585927 CG+CC for CH[(73)]

If we hypothesize that the effect of **ROSTAFUROXIN** is dependent upon the presence of mutant adducin and a/or critical level of EO in some specific tissue, this may be achieved in the individual patients by each of the above listed interactions. In fact, variations in synthesis, transport or excretion of EO may all contribute to the tissue levels of EO. Therefore, to capture all the possible genetic mechanisms at work in the individual patient, all patients carrying at least one of the above interactions were included in a specific genetic profile. In the subset of patients carrying this profile (23% of the total sample), the average SBP fall was -16.2 ± 2.2 and -2.1 ± 2.4 mmHg with **ROSTAFUROXIN** and placebo, respectively ($p < 0.0001$).
Then, we investigated whether other profiles, including also the other two genes (CYP11A1, SLC04C1) with all the possible interactions could increase the odds ratio and the systolic blood pressure difference between patients receiving **ROSTAFUROXIN** or placebo. Only profiles involving the above cited gene mutation (slightly differently associated) provided slightly better values. Such differences were not statistically significant but, most importantly, all together confirmed that the blood pressure response to **ROSTAFUROXIN** was associated with the polymorphism of the genes we predicted in 2005(41). Only further studies in large cohort of patients may provide the power to detect a possible difference among these profiles.

From candidate gene to WGAS
The genetics of complex diseases is moving from the classical paradigm: one gene-one mechanism-one clinical symptom (or phenotype) to a more complex paradigm where several factors, genetic, biological and environmental, are interacting to each others and forming a network(74-81). Accordingly, the same phenotype could be modified by a variety of genetic mechanisms. In agreement with the well established concept of genetic heterogeneity, mutations at different sites of genes (i.e. different SNPs) may produce the same alteration in protein function. Analogously, the same hormonal level may be achieved throughout different pathways (involving different genes) regulating its synthesis, metabolism or excretion. Finally, these different processes are all regulated by different genes and new functional elements of the human genome have been recently recognized.
In this respect, the ENCODE project represents the major step towards a more comprehensive characterization of all the functional elements in the human genome(82). The results of this project have clearly shown that the definition of "gene" is changing and the non-coding transcripts (non coding RNAs or ncRNAs) are a very important component of the genetic information that is transcribed in eukaryotic cells. The ncRNAs can vary in size from 18 to 10.000 nucleotides (miRNAs, siRNAs, small RNA etc.). Their functions consist in the regulation of gene expression, gene-network and alternative splicing of individual genes(83-86). Indeed, some of these functions have been shown to be involved in the regulation of genes that modulate cardiovascular functions.
Any comprehensive approach to the genotype-phenotype relationship, including PGx, must cover the involvement of these ncRNA(87,88). WGAS is the most efficient technology to detect SNPs across coding and non coding DNA regions that could affect the phenotype of interest (that in our case is reduction in blood pressure associated to placebo or **ROSTAFUROXIN**).
Another relevant aspect is emerging from the most recent studies in rodents with chromosome substitution strains aimed at dissecting the overall genetic architecture of complex traits (obesity, hypertension etc.). The sum of the individual chromosome phenotypic effects was 928% of the parental difference(89). This implies that in the parental animals the contribution of each chromosome is not additive but includes epistatic interaction, as also shown by many other

studies[90,91]. These findings limit the biological significance of all the reductionist approaches aimed at establishing the role of a given gene or set of genes.
Conversely, they call for an integrated approach that should try to establish "causality", or to approximate to it, by leaving the whole genetic architecture of the trait[92-100]. This may be carried out with WGAS.
Due to this complexity, the assessment of the genetic network affecting the response to **ROSTAFUROXIN**, needs the most comprehensive approach (from candidate genes to WGAS). However, we also need to restrict the prediction analysis to the minimal set of SNPs that, joined in a profile, provide the best prediction of the blood pressure response to the drug in our patient's sample.

In Table A is shown the comparison between Rostafuroxin genetic profiles in CEU and CH[73]. Genotype combinations of Profile 1, 2 and 3 both in CEU and in CH are indicated with different colours. Profile 2 and 3 are also included in Profile 3. Genotype changes in CH are underlined in bold and italics (see ref.101 for the explanation of these changes)

Table A: Comparison between Rostafuroxin genetic profiles in Caucasians (CEU) and Chinese (CH) [73]
Genotype combinations of Profile 1, 2 and 3 both in CEU and in CH are indicated with different colours. Profile 2 and 3 are also included in Profile 3. Genotype changes in CH are underlined in bold and italics.

Caucasians (CEU)	Chinese (CH)
**Profile 1**	**Profile 1**
ADD1 rs4961 GT+TT & ADD3 rs3731566 GG	ADD1 rs4961 GT+TT & ***ADD3 rs2501574* TT***
MDR1 rs1045642 TT & HSD3B1 rs10923835 AT+TT	MDR1 rs1045642 TT & ***HSD3B1 rs117585927 CG+CC***
LSS rs2254524 CA+AA & MDR1 rs1045642 CC	LSS rs2254524 CA+AA & MDR1 rs1045642 CC
LSS rs2254524 AA & ADD1 rs4961 GT+TT	LSS rs2254524 AA & ADD1 rs4961 GT+TT
rs16893522=AA	rs16893522=AA
rs2345088=TT	rs2345088=TT
rs2461911=AA	rs2461911=AA
rs16877182=CT	rs16877182=CT
rs12513375=GG	rs12513375=GG
HSD3B1 rs947130 GG & NEDD4L rs4245268 CC	HSD3B1 rs947130 GG & NEDD4L rs4245268 CC
MDR1 rs1045642 CC & AGTR1 rs2131127 CC	MDR1 rs1045642 CC & AGTR1 rs2131127 CC
ADD2 rs4984 CC & top rs10502933 CT	***ADD2 rs12470211 AG+GG*** & top rs10502933 CT
LSS rs2254524 AA & WNK1 rs880054 AG+GG	LSS rs2254524 AA & WNK1 rs880054 AG+GG
**Profile 2**	**Profile 2**
ADD1 rs4961 GT+TT & ADD3 rs3731566 GG	ADD1 rs4961 GT+TT & ***ADD3 rs2501574* TT***
MDR1 rs1045642 TT & HSD3B1 rs10923835 AT+TT	MDR1 rs1045642 TT & ***HSD3B1 rs117585927 CG+CC***
LSS rs2254524 CA+AA & MDR1 rs1045642 CC	LSS rs2254524 CA+AA & MDR1 rs1045642 CC
LSS rs2254524 AA & ADD1 rs4961 GT+TT	LSS rs2254524 AA & ADD1 rs4961 GT+TT
**Profile 3**	**Profile 3**
rs16893522=AA	rs16893522=AA
rs2345088=TT	rs2345088=TT
rs2461911=AA	rs2461911=AA
rs16877182=CT	rs16877182=CT
rs12513375=GG	rs12513375=GG

* ADD3 rs2501574 is in perfect Linkage Disequilibrium with rs2501578 (r2=1) in CH population.

The WGAS analysis performed in the OASIS Trial patients resulted in the data reported in Table B, both under Profile 3 and Profile 1 where additional gene interactions are included (101).The data of Profile 2 have been in part described above. The results of table B indicate that these profiles are able to define subsets of patients with very clear differences in the blood pressure responses to **ROSTAFUROXIN** and placebo or between the presence or the absence of the profile in patients receiving **ROSTAFUROXIN**. For these analysis, all the five doses (0,05; 0,15; 0,5; 1,5; 5 mg/day) of **ROSTAFUROXIN**, utilized into the OASIS Trial, have been pulled together, since no clear difference in blood pressure response was detected among them. The lack of a dose-effect relationship was already observed in rats where **ROSTAFUROXIN** produces a comparable blood pressure fall in a dose range spanning from 0,1 µg/kg to 100 µg/kg being the dose of 0,01 µg/kg inactive. However, when the blood pressure responses to the lowest doses (0,05; 0,15 and 0,5 mg/day) were pulled and analysed, their magnitude appeared to be slightly larger than that of all the doses. The relatively low sample size of this patients' cohort doesn't allow an appropriate statistical analysis of the data. However, taken together with the rat data, the results in patients suggest to include only the lowest doses in the present trial. In the bottom of the Table (part C), the lack of a profile influence on the Losartan and HCTZ blood pressure responses is also shown (CVie Therapeutics Company Limited Internal Data) (37,38).

As shown in table A, Profile 1 includes both Profile 2 and Profile 3 (Therefore, Profile 1 is used to select patients in the present Study).

Table B. Profile influence on Blood Pressure response to Rostafuroxin and placebo in the previous OASIS trial

	Profile 1$	Profile 2$	Profile 3$	
pvalue	0.000	0.0000	0.0000	
OddsRatio	20.25	9.5	14.59	
SBP fall (mmHg) on Rostafuroxin In patients with profile mean ± SE (n)	-15.17 ± 1.6 (39)<br>-17.43 ± 2.1 (22)	-16.18 ± 2.2 (24)<br>-18.49 ± 2.9 (14)	-19.42 ± 2.3 (20)<br>-23.12 ± 3.2 (10)	patients treated with all doses*<br>patients treated with low doses
Patients without profile mean ± SE (n)	0.21 ± 1.3 (62)<br>-0.24 ± 1.6 (39)	-2.01 ± 1.2 (76)<br>-2.85 ± 1.6 (47)	-2.35 ± 1.2 (81)<br>-3.17 ± 1.4 (51)	patients treated with all doses<br>patients treated with low doses
Patients on Placebo with profile mean ± SE	-2.99±1.5	-2.06±2.3	-1.35±2	
Basal SBP (mmHg) on Rostafuroxin In patients with profile mean ± SD (n)	152.54 ± 7.5 (39)<br>152.69 ± 7.1 (22)	152.22 ± 7.3 (24)<br>151.21 ± 6.5 (14)	154.26 ± 7.8 (20)<br>154.16 ± 6.7 (10)	patients treated with all doses<br>patients treated with low doses
Patients without profile mean ± SE (n)	149.48 ± 7.2 (62)<br>151.21 ± 8.2 (39)	149.98 ± 7.4 (76)<br>151.90 ± 8.2 (47)	149.78 ± 7.2 (81)<br>151.27 ± 7.9 (51)	patients treated with all doses<br>patients treated with low doses
Patients on Placebo with profile mean ± SD	149.77 ± 8	150.06 ± 8.6	149.71 ± 7.7	

$ see Table A for the corresponding rs code and genotype combination

*All doses: Rostafuroxin 0.05, 0.15, 0.5, 1.5 or 5 mg/day; Low doses: Rostafuroxin 0.05, 0.15 or 0.5 mg/day

**Rostafuroxin treated vs placebo**	**39 vs 44**	**24 vs 21**	**20 vs 26**
**Total Nr. of patients with profile**	**83**	**45**	**46**
**% with profile on total (196 patients)**	**42.3%**	**23%**	**23.4%**

For the composition of the Profiles, see table A

Table C. Profile influence on Blood Pressure response Losartan and HCTZ observed in a parallel trial carried out according to a similar design

	Profile 1 yes / no	Profile 2 yes / no	Profile 3 yes / no
Losartan (1 month) (SBP fall mmHg)	-12.03±1.4 / -13.72±1.5	-10.61±3.3 / -13.11±1	-11.37±1.7 / -14.31±1.6
HCTZ (1 month) (SBP fall mmHg)	-8.4±1 / -7.55±1.3	-8.39±1.5 / -7.87±1	-9.18±1.5 / -7.60±1
HCTZ (2 months) (SBP fall mmHg)	-11.38±1.1/ -10.31±1.2	-11.15±1.7 / -10.80±0.9	-12.35±1.4 / -10.36±1

For the composition of the Profiles, see Table A

4. TRIAL OBJECTIVES AND PURPOSE

Aim of the present Study is to demonstrate, in a prospective way, that **ROSTAFUROXIN** activity in reducing blood pressure levels in never treated patients, bearing mutations in genes encoding for adducin proteins (ADD1, ADD2, ADD3) and the enzymes involved in EO synthesis, is higher as compared to the activity of the standard treatment with a recognized effective drug like Losartan.

The two primary objectives are as follows:

to demonstrate that the highest two doses of **ROSTAFUROXIN** are able to show a statistically significant difference on reduction of office sitting systolic blood pressure at Visit 6 in comparison to the group of patients treated with Losartan 50 mg in either:

the total population bearing a mutation included in the **Genetic Profile 2.**

or

the subset of patients of the Profile 1 carrying only the **Genetic Profile 1.**

The secondary objectives are as follows:

According to the clinic blood pressure evaluations:

- to demonstrate that the highest two doses of **ROSTAFUROXIN** are able to show a statistically significant difference on reduction of office sitting systolic blood pressure in comparison to the group of patients treated with Losartan 50 mg in the subgroup of patients of the Profile 1 carrying only the **Genetic Profile 3.**
- to demonstrate that the two highest doses of **ROSTAFUROXIN** are able to show a statistically significant difference on office sitting diastolic blood pressure in comparison to Losartan in the general population and/or in the two pre-defined subgroups of patients;
- to compare one another the three doses of **ROSTAFUROXIN** and each one versus Losartan;
- to determine a dose/responder profile, if any;
- to evaluate the effect of variation of outside temperature on Blood Pressure on the different type of BP levels and measurements [102-106].

According to the 24h-Ambulatory Blood Pressure Monitoring:

- to identify the oral doses of **ROSTAFUROXIN** which lead to statistically significant differences in daytime, night-time and overall 24-hour ambulatory systolic blood pressure and/or diastolic blood pressure, in comparison with Losartan in the general population and/or in the subgroup populations, this analysis will be first run with the two highest doses of **ROSTAFUROXIN** pulled together versus Losartan and then each single dose of **ROSTAFUROXIN** will be compared vs. Losartan.

According to the overall clinic blood pressure evaluations (sitting SBP and DBP and the 24h-Ambulatory Blood Pressure Monitoring):

- To identify the oral doses of Rostafuroxin which lead to statistically significant differences, in comparison with Losartan, in either the total CEU and CH patients or in the two separate sub-populations of patients carrying at least one gene included in the Genetic Profile 2 or a combination of the same genes either with the SNPs indicated in the Genetic Profile 2 or with other SNPs harboured on the genes of the Profile 2.

Referring to the safety and tolerability:

- to determine the safety profile of the three doses of **ROSTAFUROXIN**.

5. EXPERIMENTAL DESIGN

This is a Phase II multicenter, double-blind, double-dummy, parallel group, active comparator controlled Study. Three oral doses of **ROSTAFUROXIN** (6 - 50 - 500 micrograms) will be studied versus Losartan 50 mg. The Treatment Period will last 9 weeks.
The Study will last about 15 weeks and will be structured as follows:

A Screening Visit, a Run-In Period of 15 - 42 days (and a Treatment Period of 9 weeks (additional 7 days for Visits 4, 5 and 6 are allowed in case of late Visit).

Two hundred and eighty outpatients, approximately 120 CH Taiwanese (40 patients for 50, 500, micrograms of Rostafuroxin and Losartan arms) and 160 CEU Italian (40 patients for 6, 50, 500, micrograms of Rostafuroxin and Losartan arms,) who are just diagnosed with arterial hypertension and bearing a specific genetic profile (Genetic Profile 1), not assuming diuretics, ß-blocker agents, Ca-antagonist, ACE inhibitors and AT_1-receptor blockers for other reason and finally not assuming statines, will be randomized in this Study and followed according to the standards of Good Clinical Practice. In order to be randomized, patients will have to meet all of the inclusion and none of the exclusion criteria, as specified in section 6.3.1. Diagnosis of arterial hypertension and genetic profile will be confirmed at the end of the Run-In Period (Visit 3).

Eligible patients will be randomized into one of the four Treatment arms, according to a randomization list stratified with respect of Study countries (Taiwan and Italy) and within each of them with respect of genetic subgroup (i.e. patients bearing and not bearing a mutation included in the Genetic Profile 2).
The Study will involve about 32 centres.

5.1 Study Endpoints

The primary efficacy endpoints will be:

- Change from baseline to Visit 6 in office sitting SBP.
- Proportions of responders at Visit 6, defined as "Patients having the mean office SBP (mean of last three measurements) <= 135 mmHg or having a reduction in mean office SBP (mean of the three last measurements) > or = 10% with respect to the baseline measurement" at Visit 6 (after two months of therapy).

Secondary efficacy and safety end-points will be:

- Change from baseline to Visit 6 in office sitting DBP.
- Office sitting DBP and office sitting SBP values and changes from baseline at each study visit.
- 24 hours SBP and DBP measurements. 24 hours, Day-time and night-time weighted mean value of both SBP and DBP.
- All standard safety endpoints (AEs, vital signs, ECG, laboratory data and physical examination).
- Office sitting SBP and DBP corrected as per seasonal variation of temperature (confounding factor).

5.2 Study Plan

Screening entrance condition

In order to be eligible for the Study, patients have to be aged between 25 and 60 years, have already undertaken lifestyle recommendations and still having an abnormal systolic and diastolic blood pressure levels. Furthermore, they must not have been previously treated with any specific antihypertensive drug and they must not assume drugs like diuretics, ß-blocker agents, Ca-antagonist, ACE inhibitors and AT_1-receptor blockers, for other reasons. They must not be on statines treatment or to be Diabetic (fasting plasma glucose > 125mg/dl). A short period of treatment(, not longer than 7 days, does not prevent the enrolment of the patients if a period of washout of about 1 month can be applied before the randomization.

Their value of the sitting systolic blood pressure (SBP) must range between 140 and 169 mmHg and the sitting diastolic blood pressure (DBP) must range between 85 and 100 mmHg, after an adequate period of lifestyle changes.

They do not have to present known causes of secondary hypertension, cardiac disease requiring prohibited pharmacological treatment or history of renal artery disease or a myocardial infarction occurred within the last 6 months.

All the above cited evaluations are considered routine investigations to make a correct diagnosis of arterial hypertension and assessment of the patients cardiovascular risk, therefore no specific informed consent is requested. In fact, all these evaluations are usually performed by the Physician any time a patient is presenting with elevated blood pressure levels.

Pregnant or nursing women or women of childbearing potential not taking anti-contraceptive medication are not suitable for the Study.

Finally, known renal impairment (Estimated Creatinine Clearance ≤ 50 mL/min) and obesity

(≥ 30 kg/m^2) are conditions excluding patients from the possibility to participate in this Study.

If the Clinic Centre Physician or a General Practitioner is identifying, during his/her outpatient routine practice, a patient suitable for the Study, according to the above-cited characteristics, he/she will inform the patient about the possibility to participate in such Study and if the patient declares to be interested to obtain more information about the Study, he/she will be referred for a next visit to the Clinic Centre, where a Physician will explain to him/her the Study, in full.
Because only just new discovered patients affected by mild to moderate arterial hypertension will be enrolled into the Study, the participation of General Practitioners in the identification of suitable candidate for the Study could be effective, being themselves the first contact with such patients on the field. If appropriate, the patient can be screened again with one new ICF obtained and a different subject number assigned.

If the patient has been identified by a General Practitioner, this doctor will inform the Clinic Centre of the presence of a suitable patient. The General Practitioner will send to the Clinic Centre a note of the patient medical history and of the routine tests performed to make the diagnosis of arterial hypertension and the assessment of the patient cardiovascular risk.
The Clinic Centre will contact the patient to fix an appointment at the Clinic Centre where the inclusion in the Study will be evaluated during the scheduled Screening Visit (Visit 1) and following Run-In Visits (Visits 2 and 3). The Run-In Period will follow the Screening Visit and will last from 15 up to a maximum of 42 days. Screening Visit at the Clinic Centre will consist of a confirmation of the patient suitability for the Study, signature of an informed consent and the performance of all the activities foreseen on such Visit to evaluate some of the inclusion/exclusion criteria; this Visit will be followed by the Run-In Visit 2 after a minimum of 14 days and a maximum of 42 days, and a Final Visit (Visit 3) the day after with final evaluation of the inclusion/exclusion criteria in the Study and patient randomization.
The Treatment Period will include a Clinic Visit after 2 weeks from the starting of the investigational Treatment (Visit 4); a Visit after 5 weeks from the starting of the Treatment (Visit 5); a Clinic Visit after 9 weeks of Treatment (Visit 6) and a Final Visit the day after (Visit 7).
Time window allowed for these Visits is: scheduled time plus 7 days (excluding the last visit that must be performed the day after the previous one).

Screening Visit
When the patient refers to the investigational Clinic Centre, a Physician will check again his/her blood pressure values and if they are still above the normal levels, will explain again to the patient the aim of the Study and all the activities related to the performance of the Study. After having obtained the written informed confirmation of his/her willingness to participate in the Study, the following examinations will be performed:

Visit 1 - Screening Visit
- patient's informed consent collection;
- recording of patient medical history including concomitant medications and prior medications;
- physical examination (including body weight and height measurement);
- ECG at rest;
- clinic (office) blood pressure and heart rate measurements;

- blood sampling for chemistry and haematology (within a time window of + 3 days from the Screening Visit and under fasting condition);
- urine sampling for urinalysis (within a time window of + 3 days from the Screening Visit);
- blood sampling for measurement of genotypes potentially involved in blood pressure regulation and cardiovascular or renal function, in particular, adducin and the enzymes responsible for the biosynthesis of Endogenous Ouabain and Aldosterone;
- blood pregnancy test to be performed on all women of childbearing potential.
- check of verifiable inclusion/exclusion criteria;

The Physician will give to the patient a second appointment after a minimum of two weeks and a maximum of 6 weeks, for performing the Run-In Visit 2.
The Physician will also instruct the patient how to perform a 24 Hour Urine Collection and will ask him/her to perform this collection during the 24 hours before the subsequent Visit and to bring this collection in occasion of the next scheduled Visit.

Run-In Period

Visit 2 – (between Day 14 and Day 42)

- physical examination (including body weight);
- check of concomitant medications;
- check of concomitant diseases recovery;
- clinic (office) blood pressure and heart rate measurements;
- patient's delivering of the 24 hours urine collection.
- start measurement of the 24 hours ambulatory blood pressure;

The Physician will ask to the patient to come back the day after to stop the blood pressure monitoring. If subject is failed to meet the BP criteria on the day of V1 or the laboratory data out of criteria, the re-test is allowed in run-in period. Genotyping could not be re-tested.

Visit 3 - The Day After Visit 2

- stop of the 24 hours ambulatory blood pressure measurements;
- check of concomitant medications;
- check of concomitant diseases recovery;
- clinic (office) blood pressure and heart rate measurements;
- blood sampling for Endogenous Ouabain (EO);
- check of all inclusion/exclusion criteria;
- randomization and drug dispensing for the subsequent Study period;
- delivery to the patient of the Patient's diary;
- delivery to the patient of a letter for the General Practitioner
- schedule the next appointment (2 weeks later).

Treatment Period

Patient will start to assume the Study Drug from the next morning after the Randomization Day, before breakfast. He/She will report all adverse events, if any, in the patient's diary and will come back to the clinic for the next scheduled Visit after two weeks of Treatment (time window allowed for this Visit is: scheduled time + 7 days). Patient will also be instructed to contact the Clinic Centre in case of serious adverse events and inform the General Practitioner.

Visit 4 - Day 14 (Two weeks after the starting of the Treatment Period. Time window allowed for this Visit is: scheduled time + 7 days):
- physical examination (including body weight measurement);
- clinic (office) blood pressure and heart rate measurement;
- check of concomitant medications;
- check of concomitant diseases recovery;
- recording AEs, if any;
- scheduling of the next appointment (3 weeks later. Time window allowed for this Visit is: scheduled time + 7 days).

Visit 5 - Day 35 (Five weeks after the starting of the Treatment Period. Time window allowed for this Visit is: scheduled time + 7 days):
- physical examination (including body weight measurement);
- clinic (office) blood pressure and heart rate measurement;
- check of concomitant medications;
- check of concomitant diseases recovery;
- ECG at rest;
- recording AEs, if any;
- Study Drug accountability relevant to the capsules returned by the patient;
- Study Drug dispensing for the subsequent Study period;
- scheduling of the next appointment (4 weeks later. Time window allowed for this Visit is: scheduled time + 7 days).

Visit 6 - Day 63 (Nine weeks after the starting of the Treatment Period. Time window allowed for this Visit is: scheduled time + 7 days):
- physical examination (including body weight measurement);
- clinic (office) blood pressure and heart rate measurement;
- blood sampling for chemistry and haematology (within a time window of - 3 days from the scheduled Visit and under fasting condition);
- blood sampling for EO;
- blood pregnancy test to be performed on all women of childbearing potential.
- urine sampling for urinalysis (within a time window of - 3 days from the scheduled Visit);
- check of concomitant medications;
- check of concomitant diseases recovery
- recording AEs, if any;
- starting of measurement of the 24 hours ambulatory blood pressure.

The Physician will ask to the patient to come back the day after to stop the blood pressure monitoring and perform the Final Study Visit.

Visit 7 – The Day After Visit 6
During this Visit, the following procedures will be performed:
- stop of the 24 hours ambulatory blood pressure measurements;
- clinic (office) blood pressure and heart rate measurement;
- ECG at rest;
- check of concomitant diseases recovery;

- check of concomitant medication;
- Study Drug accountability relevant to the capsules returned by the patient;
- delivering by patient of the filled in patient’s diary;
- recording AEs if any;
- prescription of the standard antihypertensive treatment

5.3 Study Diagram

	Screening Visit	Run-In Period		Treatment Period			
	Visit 1 (Screening)	Visit 2 (between Day 14 and 42)	Visit 3 V2+1The day after Visit 2	Visit 4 (Day 14) Time window allowed: +7 days	Visit 5 (Day 35) Time window allowed: +7 days	Visit 6 (Day 63) Time window allowed: +7 days	Visit 7 (Day 64) The day after Visit 6
Patient's Informed Consent	X						
Past/Present Medical History	X						
Prior Medications	X						
Concomitant Medications	X	X	X	X	X	X	X
Physical Examination Including Body Weight and Height Measurement (only at Visit 1)	X	X		X	X	X	
Office Blood Pressure (OBP)	X	X	X	X	X	X	X
Heart Rate Measurement (HR)	X	X	X	X	X	X	X
ECG at Rest	X				X		X
Blood Sampling for Chemistry, Haematology and Pregnancy Test	X					X	
Urinalysis	X					X	
Blood Sampling for Genotype Determination	X						
Check of Inclusion/Exclusion Criteria	X		X				
Check of Concomitant Diseases Recovery		X	X	X	X	X	X
24 Hours Ambulatory Blood Pressure (with patient diary)		X…………	…………X			X…………	…………X
24 Hours Urine Collection [1]		…………X[1]					
Blood Sample for Endogenous Ouabain (EO)			X			X	
Randomization			X				
Delivery to the Patient of the Patient's Diary			X				
Delivery to the Patient of a Letter for General Practitioner			X				
Study Drug Dispensing			X		X		
AEs Recording (if any)				X	X	X	X
Study Drug Accountability					X		X
Delivering by the Patient of the Filled-In Patient's Diary							X
Prescription of the Standard Antihypertensive Treatment							X

[1] **- 24 Hours Urine Collection will start the 24 hours preceding the Visit 2.**

6. STUDY POPULATION

6.1 Sample Size

Two hundred and eighty patients are planned to be randomized in this Study, 160 CEU Italian patients and 120 CH Taiwanese patients. They will be allocated to four parallel arms (80 patients for 50, 500, micrograms of Rostafuroxin and Losartan arms, and 40 CEU Italian patients in 6 micrograms of Rostafuroxin arm). Approximately 50% of the whole sample is expected to bear a mutation included in the Genetic Profile 2. Therefore, the sample size of each arm in this pre-defined subgroup bearing Genetic Profile 2 will be approximately of 40 patients for 50, 500, micrograms of Rostafuroxin and Losartan arms, and 20 CEU Italian patients in 6 micrograms of Rostafuroxin arm.
An interim sample size re-assessment is planned and will be performed when approximately half of the patients will reach the final evaluation.

6.2 Parameters Identifying Study Population

To be eligible for the Study and to be randomized in the Treatment Period, patients will have to meet all of the Screening verifiable criteria first and then, before the randomization, all of the inclusion and none of the exclusion criteria, hereafter specified.
Eligible patients will be randomised to receive one of the doses of **ROSTAFUROXIN** (6, 50 or 500 micrograms for CEU Italian patients, and 50 or 500 for CH Taiwanese patients) or Losartan 50 mg.

6.3 Patients Selection

6.3.1 Study selection criteria

If the patient fulfils all the criteria that made him/her suitable for taking part in the Study and after having been informed about the possibility to participate in this Clinical trial and if interested, he/she will be referred for a next visit to the Clinic Centre where the following criteria will be evaluated during the Screening and the Run-In Visits:

<u>Inclusion Criteria</u>

- Signature of a written informed consent, included informed consent on genotype analysis.
- Male/female patient aged 25-60 years.
- Naive hypertensive patient (new diagnosed patient, never treated before or, if treated for a short period (up to 7 days), a washout of 1 month before randomization can be applied).
- Documented mild to moderate arterial hypertension, as defined below:
    - At Visits 1 and 2 the mean of the last 3 consecutive readings of SBP must be $\geq$ 140 mmHg and DBP must be $\geq$ 85 mmHg, when measured by Clinic (OBP);
    - At Visit 3, the mean of the last 3 consecutive readings of SBP must be $\geq$ 140 mmHg $\leq$ 169 mmHg and DBP must be $\geq$ 85 mmHg and $\leq$ 100 mmHg, when measured by Clinic (OBP).
- Presence of at least one mutated genotype or combination of genotypes corresponding to the list provided in Genetic Profile 1

<u>Exclusion Criteria</u>

- Known causes of secondary hypertension;
- Severe or malignant hypertension;

- History of renal artery disease;
- Significant renal (estimated creatinine clearance ≤ 50 mL/min) or hepatic disease (SGOT and/or SGPT greater than 2 times the upper limit of the normal range);
- Cardiac disease requiring prohibited pharmacological treatment or history of myocardial infarction within the last 6 months;
- Atrial Fibrillation;
- Complete Left and/or Right Ventricle Bundle Branch Block;
- First degree AV-block exceeding 240 msec;
- Electrocardiographic evidence of left ventricular hypertrophy, defined either as a Cornell voltage (6 mm adjustment in women) × QRS duration product exceeding 2440 mm × ms or as a Sokolow-Lyon voltage index of more than 38 mm (for technical details, see Circulation 1987; 75: 565-72 & JACC 1995; 26: 1022-29);
- Pregnant or nursing women or women of childbearing potential not taking anti-contraceptive medication or not utilizing a double contraceptive method (i.e.: intrauterine device plus condom);
- Surgery or disease of the gastrointestinal tract, which might influence absorption or elimination of the drug;
- Any concomitant condition that may, in the judgement of the Investigator, jeopardise participant adherence to the Protocol or ability to complete the trial (e.g., alcohol or drug abuse, disabling or terminal illness, personality or mental disorders, etc.);
- Concomitant therapy with medications that may affect blood pressure;
- Treatment with any investigational drug in the previous 6 months;
- Predictable lack of cooperation;
- Obesity ≥ 30 kg/m^2;
- Diabetes mellitus (fasting plasma glucose > 125 mg/dl).
- Statines treatment.

At the end of the Run-In Visits, if all the randomization selection criteria are fulfilled, patient will be randomized and Investigator will deliver to him/her the first box of Study Drug and the patient diary.
Those patients who will not result to be bearers of one of the gene mutations suitable for the Study, or not fulfilling all the selection criteria, will be given a standard antihypertensive treatment. Furthermore, they will be referred to the General Practitioner for being followed up. The knowledge of their specific genotype could in any case to drive the medical doctor to prescribe them the most successful treatment, according to the present status of art.

6.4 Removing Patients from the Study

The following conditions are foreseen for patient withdrawal from the Study:

- Informed consent withdrawal / Patient's request;
- SAEs, serious and unexpected worsening of clinical conditions, or any other reasons for which, in the Investigator's judgement, it is inadvisable for a patient to continue the Study;
- Uncontrolled hypertension (**SBP ≥ 179 mmHg or DBP ≥ 110 mmHg**) measured at the clinic by the Investigator. Before removing the patient from the Study, the Investigator has to retest once more the patient blood pressure within one week. If the clinic blood pressure is still above the previous indicated limits, the patient must be removed from the Study.

- Occurrence of disease(s) which can interfere with patient's final evaluation.
- Administration of prohibited drugs.
- Postural hypotension: In case the patient should refer symptoms ascribable at postural hypotension, the Investigator will give the patient all the conduct information to reduce such symptoms and ask to the patient to come to the clinic as soon as possible for an unscheduled Visit. On this occasion, the criterion for determining postural hypotension will be the difference between the sitting systolic blood pressure and the standing systolic blood pressure. If this difference is greater than 40 mmHg, the definition of postural hypotension will be considered met and the patient will be removed from the Study.

Patients will be replaced if their discontinuation is not due to uncontrolled hypertension (see above BP limits) or postural hypotension unless these blood pressure changes occur within the first month of treatment. If the discontinuation occurs at least after one month of study treatment administration discontinued patients will not be replaced. Any reason leading to the patient discontinuation will be properly recorded in the CRF. In case of patient discontinuation due to SAE or AE, the evolution of the SAE or AE will be followed up and recorded by the Investigator till the outcome definition. If possible, patients removed from the Study should perform concomitantly with the Study Drug interruption, a final physical examination, an ECG recording, a check of the concomitant medications, a blood sampling for chemistry and haematology, a recording of the clinic (office) blood pressure and heart rate.

7. TREATMENT OF PATIENTS

Three hundred and twenty patients will be randomised to receive **ROSTAFUROXIN** capsules (6 - 50 - 500 micrograms once daily) or Losartan 50 mg, tablets once daily according to the randomization list.

7.1 Lifestyle Recommendations

In agreement with current guidelines [107], the institution of non-drug measures to control the blood pressure is recommended. These lifestyle measures include cessation of smoking, weight reduction in overweight patients, moderate salt restriction (120-160 mEq Na/day), dietary measures to control hypercholesterolemia (if applicable), moderation of alcohol intake and regular physical activity.

They should be equally reinforced in all patients during the evaluation of the General Practitioner/Physician and during all the Study Period.

However, major dietary changes (e.g. weight reduction or salt restriction) should not be made after starting the Treatment Period.

7.2 Study Treatments

ROSTAFUROXIN and Losartan 50 mg will be administered in this Study once daily for 9 weeks, under double-blind, double-dummy conditions. Each patient will be randomized to one of the following treatment schedule:

- 1 capsule of **ROSTAFUROXIN** (6 micrograms) plus one tablet of placebo of Losartan, once a day before breakfast (only CEU Italian patients);

- 1 capsule of **ROSTAFUROXIN** (50 micrograms) plus one tablet of placebo of Losartan, once a day before breakfast;
- 1 capsule of **ROSTAFUROXIN** (500 micrograms) plus one tablet of placebo of Losartan, once a day before breakfast;
- 1 tablet containing Losartan 50 mg plus one capsule of placebo of **ROSTAFUROXIN**, once a day before breakfast.

7.2.1 Dosage, Posology and Administration

The three different doses of **ROSTAFUROXIN** have been selected according to previous experimental data. The two highest doses (50 - 500 micrograms) should demonstrate capability to significantly reduce the systolic blood pressure, while the lower dose (6 micrograms) is investigated to collect information about the minimum effective dose. Losartan 50 mg has been selected as active comparator, according to its efficacy showed in previous controlled clinical trials. Patients will be asked to take both one capsule containing one of the dose of **ROSTAFUROXIN** or placebo of **ROSTAFUROXIN** and one tablet containing placebo of Losartan or Losartan 50 mg. The study medications should be preferably taken at around 08.00 in the morning before breakfast. On Study Visit days the Study Medication will be taken at 08.00 in the morning before breakfast and the Clinical Visits have to be performed within the following 6 hours. Each Clinic Centre must carefully standardise Visit time for each patient in order to reduce the difference of time from drug assumption till clinic (office) blood pressure measurements, within different Visits.

7.2.2 Composition of Used Treatments

<u>Composition of ROSTAFUROXIN and Placebo hard gelatine capsules:</u>

**Ingredients**	**Unit Formula (Active)**		
**Active**<br>PST2238	**6 µg**<br>0.006 mg	**50 µg**<br>0.050 mg	**500 µg**<br>0,500 mg
**Excipients**			
Mannitol	30.244 mg	80.20 mg	79,75 mg
Microcrystalline cellulose	10 mg	10 mg	10 mg
Hydroxypropylcellulose	5.0 mg	5.0 mg	5.0 mg
Crospovidone	3 mg	3 mg	3 mg
Sodium lauryl sulphate	1.0 mg	1.0 mg	1.0 mg
Magnesium stearate	0.75 mg	0.75 mg	0.75 mg
Opaque white capsule shell size 4	1	1	1
Opadry clear	30.58 mg	30.58 mg	30.58 mg
Acryleze clear	25.44 mg	25.44 mg	25.44 mg

**Ingredients**	**Unit formula (Placebo)**
Mannitol	80.25 mg
Microcristalline cellulose	10.00 mg
Sodium lauryl sulphate	1.00mg
Hydroxypropylcellulose	5.00 mg
Crospovidone	3.00 mg
Magnesium stearate	0.75 mg
Opaque white capsule shell size 4	1
Opadry clear	30.58mg
Acryleze clear	25.44mg

Composition of Losartan and Placebo tablets:

Ingredients*	Unit Formula (Active)
Losartan Potassium	50.0 mg
Microcrystalline Cellulose	52.5 mg
Lactose Monohydrate	25.5 mg
Progelatinized Corn Starch	20.95 mg
Magnesium Stearate	1.05 mg
Hydroxypropyl Cellulose LF (with <0.3% silica)	1.80 mg
Hypromellose	1.80 mg
Titanium Dioxide E171	0.90 mg
Carnauba Wax	0.05 mg

*no other ingredient has been added to the authorized product

Ingredients	Unit formula (Placebo)
Microcrystalline Cellulose	77.5 mg
Lactose Monohydrate	50.5 mg
Progelatinized Corn Starch	20.95 mg
Magnesium Stearate	1.05 mg
Hydroxypropyl Cellulose LF (with <0.3% silica)	1.80 mg
Hypromellose	1.80 mg
Titanium Dioxide E171	0.90 mg
Carnauba Wax	0.05 mg

7.2.3 Packaging and Labelling

All IMPs (Rostafuroxim capsules at all dosages), IMP comparator (Losartan tablets) , placebo matching Rostafuroxin capsules and placebo matching Losartan (tablets) are manufactured, packaged, labelled , tested and certified for clinical use according to the principle of GMP and Local Legislation for each Country involved.

Complete details and requirements for study drug packaging, labeling, storage, preparation, administration and accountability are written in a separate document that will be available in a local language for each Country.

Labeling details will be provided to the Ethical Committee for evaluation, if required by the Local Legislation.

The Pharmacy of the Clinic Centres involved in the Study and the Investigator will be responsible for receipt and proper storage of the Study medication, which will be kept in a secure location, for the whole Study duration and maintained at temperature between 2 and 8°C in a secure location, with a maximum out of range time period of seven days at under 25°C as indicated in the Investigator's Brochure, and according to instructions for use provided by CVie Therapeutics Company Limited.

Each patient box will contain the two boxes relevant to the First and the Second Period of Treatment, containing the quantity of Study medications necessary to cover respectively the 5 or

4 week Treatment Periods plus extra-days in the event that a patient is not able to return on foreseen days.
In each Treatment Period box there will be one bottle containing 45 capsules of **ROSTAFUROXIN** or Placebo and one case containing 4 blisters containing 14 tablets/blister of Losartan or placebo, labeled as Treatment Period 1 or 2, Weeks 1-5 and Weeks 6-9, respectively and with all the information required by GMP Guidelines.
All the labels will be printed in local language.

7.2.4 Drug Accountability

Patients will be instructed by the Investigator to return unused Study Medication at specific Visits. All returned capsules and tablets will be counted and recorded on the appropriate CRF page. The Investigator will be responsible for receipt, proper storage and security of the returned Study Medication. All unused and partially used bottle or blisters packs of Study Medication will be returned to the Sponsor at the end of the Study.

7.3 Patients' numbering

An Enrolment Number (two digits) assigned in chronological order will identify each single patient starting from Screening Visit (Visit 1) in each centre. A Randomization Number (four digits) to each patient will be assigned at the end of the Run-In Period (Visit 3), if all the inclusion/exclusion criteria are satisfied.
Finally, another number of three digits will identify the Clinic Centre that is following the patient: the first number will identify the Country, the other two the Clinic Centre.

7.4 Randomisation

A computerized procedure generating pseudo-random numbers will be used. Assignment to treatment groups will be determined by a computer-generated random sequence using an interactive web-based response system (IWRS).
The randomization will be stratified by Study Country (i.e. Taiwan and Italy) in order to balance the different treatments (four arms in Italy and three arms in Taiwan) within different geographical areas and, within each Country, by genetic subgroup (one stratum will be constituted by the patients bearing a mutation included in the Genetic Profile 2, the other stratum will comprise all remaining patients). Blocks will be used for assuring the balance of the treatment allocations.
The randomization list will be generated by the same Contract Research Organization in charge to implement the centralized web system for Randomization of the patient and Treatment Number allocation (OPIS s.r.l.).

To preserve study blinding, access to the randomization table and treatment assignments will be restricted to a minimum number of people: to the Responsible of Pharmacovigliance for each Country, and to the Manufacturers Responsible for drug packaging prior to database lock.
If a subject is unblinded, he must be discontinued from the study.
The Treatment assignment will be carried-out under blind conditions throughout a secured and dedicated web site.
When a patient has qualified as eligible (i.e. he/she fulfils all the inclusion criteria and does not carry any exclusion criteria of the Study Protocol), the Investigator or his/her designee will

request the patient's randomization code. Each Study centre must request these randomization codes for all their patients in the order to qualify them for the Study.

The procedure for generating the list, maintaining the blinding, managing the random allocation and unblinding the individual randomisation codes, if needed, will be detailed in a specific document.

The Investigators will be allowed to break individual codes exclusively when a Serious Adverse Event occurs and knowledge of the treatment by the Investigator is deemed useful as to the patient's safety. Individual code breaking must be clearly indicated in the patient related SAE form.
Breaking of individual randomization codes is also allowed:

- to the Drug Safety Unit of CVie Therapeutics Company Limited, or its Pharmacovigilance Responsible delegate for Italy during the course of the Study, in order to comply with the regulatory requirements;
- to the Data and Safety Monitoring Board (DSMB), in order to give advice to the Sponsor in case of safety concerns. In this case, the DSMB will ask to the Drug Safety Unit of CVie Therapeutics Company Limited, to open the code and give them notice of the treatment.

7.5 Prior and Concomitant Therapies

All the medications given during the last three months prior to Study initiation and the concomitant medications given during the course of the Study will be recorded on the CRF, indicating name of the drug, route of administration, dosage, therapy duration and reason for administration.
Any medication stopped before starting the trial will be considered as 'prior', while drug therapies to be continued by the patient, or to be started during the trial will be recorded as 'concomitant' medications.

Prohibited drugs
The following treatments will not be allowed during the Run –in period of the Study:

- Concomitant antihypertensive or anti-anginal treatments (such as ACE inhibitors, angiotensin II antagonists, Ca-channel blockers, beta-blocking agents, alpha-methyldopa, prazosin, reserpine, and other centrally active antihypertensive drugs), diuretics;
- medications causing systemic vasodilation or vasoconstriction such as theophylline, papaverine, trycyclic antidepressants, neuroleptics, long-acting nitrates, sympathicomimetic nasal agents;
- anti-arrhythmic agents and digitalis;
- non-steroid anti-inflammatory agents (with the exception of aspirin at low doses) for a period longer than 1 week;
- chronic use of steroids;
- immunosuppressive or cytotoxic agents;
- statines.

A short treatment period of a maximum of 7 days of the above listed treatments not limit the enrolment of a patient, but it is mandatory a wash –out period of at least 28 days before randomisation.

The occasional use of short-acting nitrates will be allowed (on demand).

7.6 Compliance

Patients' treatment compliance will be evaluated by counting the number of returned capsules and tablets and comparing this count with the number of capsules and tablets dispensed at the previous Visit.
Patients' treatment compliance will be assessed at the end of the Study.
Acceptable compliance is defined as taking at least 80% of the prescribed medication and not having discontinued the Study Drug Treatment during the day before or the day of each scheduled Visit. Patients not fulfilling these criteria will be considered not-compliant.

7.7 Treatment Suspension/Discontinuation

The Investigator can suspend the Study Medication at any time on the basis of his/her judgement. Additionally, the patient can refuse to continue the treatment. Reasons for removing patients from the Study are detailed in the section 6.4.

Any treatment suspension or discontinuation must be recorded on the CRF by the Investigator, who will indicate date, duration (in case of treatment suspension) and reasons of treatment interruption. Specific question will be addressed to the patient on each Treatment Visit, relevant to the drug assumption in the morning of the Visit day and the morning before.

8. ASSESSMENT OF EFFICACY

8.1 Primary Efficacy Parameter

8.1.1 Clinic (Office) Blood Pressure Measurement (only systolic blood pressure results are indicated as primary efficacy parameter)

Calibration of the device: Automated oscillometric device ***(Omron 705IT or superior models)*** will be utilised to evaluate blood pressure in clinic. Any device before using, and then annually, must be calibrated according to the Manufacturers recommendations and Hospital procedure.

At the Screening Visit (Visit 1), arm circumference will be measured and the appropriate cuff size determined. A cuff with 12 x 22 cm bladder is acceptable if arm circumference does not exceed 32 cm. In contrast, if arm circumference does exceed 32 cm, cuffs with a larger bladder (at least 15 cm x 31 cm) are required. Large cuffs can be used on thin arms, but the opposite is not acceptable.
At the Screening Visit (Visit 1), the Investigator must also ascertain that there is no clinically significant difference in the blood pressure readings obtained at the left and right arm. After 5 minutes rest in the sitting position, the blood pressure will be measured with the automated oscillometric device ***(Omron 705IT or superior models)*** on both arms, always starting with the non-dominant arm. The interval between these two readings must be less than one minute. If

systolic and diastolic blood pressure are within 10 mmHg at either side, all blood pressure readings must be obtained using the non-dominant arm. In all other patients, the arm giving the highest blood pressure readings must be chosen.
Once that the cuff and the arm have been selected, all types of blood pressure measurements throughout the Study (conventional and ambulatory blood pressure measurement), must be performed with the selected cuff and chosen arm.
Main outcome measures will be based on automated blood pressure measurements in the clinic after the patient has been seated for 10 minutes in a quiet room. The patient's chair should provide comfortable back support.

Automated sitting and standing SBP and DBP will be recorded at each Visit in order to select the patients and evaluate the antihypertensive effect of the different oral doses of **ROSTAFUROXIN** and Losartan:

- sitting: after the patient has rested for at least 10 minutes in a quiet room. There are five consecutive sitting BP readings with a 30 to 60 seconds interval between the readings; the mean of the last three sitting BP will be used for decision making, with respect to inclusion or exclusion from the Study and to evaluate treatment response;
- after the sitting readings have been completed, the standing BP is measured twice immediately after the patient assumed the standing position.

Measurements are made for one patient throughout the Study:

- in the same room, possibly in the same time of the day;
- by the same Investigator or member of the Investigator's staff;
- on the same arm;
- using a validated automatic oscillometric system with printing capacity;
- using a proper sized cuff.

8.2 Secondary Efficacy Parameters

8.2.1 Clinic (Office) Sitting Diastolic Arterial Blood Pressure (DBP)

Diastolic blood pressure measurements will be performed at the same times of the systolic blood pressure measurements, as described above.

It will be investigated the effect of the variation of outside temperature (°C) on SBP and DBP. The minimum and maximum daily temperatures will be measured at WMO station (World Meteorological Organization) or by Datameteo and collected into an independent database.

8.2.2 24-Hours Systolic and Diastolic Blood Pressure Monitoring

A validated device must be utilised to collect data. The recorder will be programmed to acquire measurements every 15 minutes from 8 AM to 10 PM and every 30 minutes for the remaining time of the day and night. The total time of recording will be 25 hours to be sure to obtain 24 hours of valid recording. A tolerance of ± 2 hours will be admitted for holter recording. The same cuff-size and arm selected in occasion of the clinic (office) blood pressure recording must be used. Ambulatory recordings will be started in occasion of the second Run-In Visit (Visit 2) and the Visit performed at the end of the Treatment Period (Visit 6). Ambulatory recordings will be scheduled on days which are normal working days for the patients.
Before applying the device, the patient will be seated for 5 minutes, then the cuff of the blood pressure monitor will be applied. Two test readings will be obtained, then the monitor cuff will

be replaced with that of the automated oscillometric device and two more readings will be obtained by the Investigator and recorded on the CRF. Thereafter, the cuff of the monitor will be applied again and two test readings obtained again. Finally, ambulatory recording will start. Patient will receive the Patient's Holter Diary Card and a phone number to be called, should technical problems arise during the recording.
Ambulatory blood pressure recording will not be considered trustable if one of the following conditions are applicable:

- less than 10 readings between 10.00 and 20.00 hours;
- less than 5 readings between midnight and 06.00 in the morning;
- the recording covers less than 20 hours;
- no valid (i.e. without error code) readings are available for 3 or more consecutive hours.

For each patient's examination, the Investigator must send to the Centralized Reading Centre of the Study specific for Country, using a dedicated web portal. Data collected on the Patient's Holter Diary Card and the Ambulatory Blood Pressure Recording will be input on the appropriate page of CRF. The local Investigator must retain a back-up copy of the file he/she has sent to the Centralized Reading Centre. The Centralized Reading Centre will be in charge for data interpretation

9. BLOOD SAMPLING

All samples for chemistry and haematology (Na, Crea, etc.) will be processed by each centre.

9.1 DNA Management

At the Screening Visit (Visit 1), a fasting blood sample will be collected in order to perform the genotype analysis.

- 10 ml of whole blood will be collected in 2 EDTA-tubes, or one 10ml EDTA-tube, gently mixed and centrifuged at room temperature within 2 hrs (10 min., 3000 rpm).
- Discard the supernatant and transfer the pellet in a Nalgene tube (5 ml); this material will be utilised to identify genetic polymorphisms associated with blood pressure.
- Each sample must be properly marked with patients' enrolment number and date of sample taken.
- All samples must immediately be frozen at or below -20°C and stored in the shortest possible time.

As soon as possible the collected pellet will be sent to the Central Laboratory (see paragraph 9.2 for shipping address).

Patients will be investigated first to verify if they belong to the **Genetic Profile 1** Then, it will be checked if they also belong to the subset of patients having **Genetic Profile 2**).
This information will be used as a basis for randomizing the patient to the Study Treatments.

Finally, it will be verified if the patients belong to the **Genetic Profile 3** . This additional genetic information will be simply recorded in the Case Report Form, i.e. it will have no effect on randomization.

Details of the single genes detected will not be reported in the CRF but only the information if the patient belongs to the Genetic Profile 1 and 2 or 3.
Only patients belonging to the Genetic Profile 1 (which includes Genetic Profiles 2 and 3) will be randomized in the Study.

All the samples for the DNA analysis will be kept by the Division of Nephrology and Hypertension - San Raffaele Hospital - University of Milan for a maximum period of 2 years starting from the end of the study and then will be destroyed.

9.2 Endogenous Ouabain Management

At Visit 3 and Visit 6 a fasting blood sample will be collected in order to perform the EO measurement.

- Collect 10 ml of blood in 2 EDTA tubes (5 ml tubes) or one10ml EDTA-tube;
- Centrifuge at 3000 RPM to separate plasma (supernatant) from blood cells (pellet);
- Prepare a 5 ml cryovial with external cup: write, directly on it, patient's enrolment number and date of sampling;
- Transfer the supernatant of both tubes in the cryovial and fill it with 4.5 ml of plasma;
- Freeze it at or below -20°C until shipping;

Every 1-2 months the stored samples will be sent together with a request form and the sample list.

Shipping information for DNA (and EO for Italy):

Dr. Nunzia Casamassima
L32-L33 - 1° Q
Division of Nephrology and Hypertension
San Raffaele Hospital
University of Milan
Via Olgettina, 60
20132 Milan - ITALY

Shipping information for EO from Taiwan:

Dr. Wang Jiguang
Ruijin Hospital Affiliated to The Shanghai Jiao Tong University Medical School
No.197, Rui Jin Er Road,
Shanghai- CHINA

10. ASSESSMENT OF SAFETY AND TOLERABILITY

10.1 Safety and Tolerability Parameters

ROSTAFUROXIN and Losartan safety and tolerability will be evaluated by means of ECG, vital signs, laboratory investigations (haematology, chemistry, urinalysis), concomitant medications and monitoring of the adverse events occurrence.

10.1.1 Heart Rate (HR)

At each Visit, heart rate is recorded once at the end of the sitting BP determinations and once after the standing BP determination.

10.1.2 Electrocardiogram (ECG)

A 12-lead ECG (paper speed 25 mm/sec) will be obtained at the Screening Visit (Visit 1) and after 5 and 9 weeks of Treatment (Visits 5 and 7).
The ECG-room should have an ambient temperature, which is sufficiently elevated to avoid muscle tremor.
Each ECG should include a calibration signal and must be of sufficient quality to measure voltages reliability. The ECG must be recorded during a short (15 - 30 sec) end-expiratory apnoea to avoid variability of the amplitude of the QRS voltages and time intervals.
Each ECG should contain complete patient identification (initials, date of birth, enrolment number and date).
The following parameters will be recorded HR, PR, RR, QT and QTcB interval and the whole tracing will be interpreted. The Investigator must record the medical report on the CRF.

10.1.3 Laboratory Evaluation

Routine Urine, Haematological and Serum Chemistry

In this Clinical Trial, blood sample and urine will be collected to perform urinalysis and routine laboratory tests to monitor biohumoral conditions of patients before their exposition to the investigational drugs (Screening Visit (Visit 1)) and at the end of the Treatment Period (Visit 6).
Blood samples should be obtained in the morning with the patient in fasting condition and at last one hour for away from clinic (office) blood pressure measurements.
All laboratory examinations will be performed by the Local Laboratory of the Investigational centre.
Results will be reported in SI units or in conventional units.

a) Hematology and Blood Chemistry Tests

Haemoglobin, haematocrit, red blood cells, white blood cells, neutrophils, lymphocytes, monocytes, eosinophils, basophils, platelet count, sodium, potassium, calcium, inorganic phosphorus, total protein, albumin, SGOT/AST, SGPT/ALT, alkaline phosphatase, γ-GT, total bilirubin, glucose, total cholesterol, LDL cholesterol, triglycerides, creatinine, uric acid.

b) Urinalysis

In the mornings of the scheduled laboratory examinations, a fresh urine sample will be obtained. Specific gravity, pH, presence of glucose, proteins, hemoglobin, ketones and bilirubin will be evaluated. At visit 2 a 24 hours urine test will be performed to measure sodium levels.

c) Pregnancy Test

All the childbearing potential women will perform a rapid blood pregnancy test at Screening Visit (Visit 1) and at the end of the Study (Visit 6) to exclude that they are pregnant before starting the investigational treatment and did not get pregnant during the Study Drug administration.

Abnormal Laboratory Test Results

The Investigator should mark in the CRF the laboratory values out of normal ranges of clinical importance. The abnormal values occurred during the Treatment Period, will be considered as AEs and the proper AE reporting procedure will be followed up by the Investigator, unless the measured laboratory abnormality was pre-existing and related to a concomitant disease(s) already recorded on the CRF at Study entry.
The abnormal laboratory test should be immediately repeated and followed up until the abnormal value has returned to normality, stabilized or an adequate explanation should be given if the abnormal value becomes stable.

10.1.4 Adverse Events (AE) and Serious Adverse Events (SAE)

The Investigator is responsible for the detection, recording and documentation of events meeting the criteria and definition of an adverse event (AE) or a serious adverse event (SAE) as provided in this Protocol.

Adverse Event

Any untoward medical occurrence in a patient or clinical-trial patient administered a medicinal product and which does not necessarily has to have a causal relationship with this treatment.
An adverse event can therefore be any unfavourable and unintended sign (e.g. an abnormal laboratory finding), symptom, or disease temporally associated with the use of a medicinal product, whether or not considered related to the medicinal product.

Adverse Reaction

A response to a medicinal product which is noxious and unintended and which occurs at doses normally used in man for the prophylaxis, diagnosis or therapy of disease or for the restoration, correction or modification of physiological function

For the reporting of adverse reactions occurring in clinical trials all untoward and unintended responses to an investigational medicinal product related to any dose administered are considered adverse reactions

AEs have to be classified by the Investigator as "Serious" or "Non Serious", according to the following definitions:

Serious Adverse Event/ Reaction

It means an adverse event/reaction which results in death, is life-threatening, requires in-patient hospitalisation or prolongation of existing hospitalisation, results in persistent or significant disability or incapacity, or is a congenital anomaly/birth defect.

Life threatening in this context refers to a event/reaction in which the patient was at risk of death at the time of the event/reaction; it does not refer to a event/reaction that hypothetically might have caused death if more severe.
Medical and scientific judgement should be exercised in deciding whether other situations should be considered serious events/reactions, such as important medical events that might not be immediately life threatening or result in death or hospitalisation but might jeopardise the patient or might require intervention to prevent one of the other outcomes listed above. Examples of such events are intensive treatment in an emergency room or at home for allergic bronchospasm, blood

dyscrasias or convulsions that do not result in hospitalisation or development of dependency or abuse.

After the informed consent has been signed at the beginning of the Run-In Period, medical events that match one or more criteria for seriousness must be reported as SAEs also in those cases in which treatment administration was not started. In fact, there may be an interference of the Study procedures on the standard medical therapy. These events will be reported in the SAE form and in the "Medical/Surgical History" page of the CRF.

Adverse Events that do not meet any of the criteria defining SAEs.
Some specific requirements are provided in this Study concerning the type of events to be reported, e.g.:

- Hospitalization for routine treatment or monitoring of the studied indication, not associated with any deterioration in condition is not a SAE.
- A surgical intervention or hospitalization, planned before the Study entry but that should be performed during the trial, must be recorded on the CRF in the AE pages (an ad hoc flag must be ticked for indicating that hospitalization was pre-planned). It should not be considered as SAE, unless it must be performed earlier than planned, and the Investigator suspects that this is treatment related; in fact, it is not the hospitalization itself, but the event causing patient's hospitalization that should be evaluated and, if judged serious, reported as SAE.

Causality and Severity Assignment

For each event, the Investigator has to assign the Study Treatment causality according to the following categories:

- suspected: to be used for an AE which is suspected to be related to the medicinal drug;
- not suspected: to be used for an AE which is not suspected to be related to the medicinal drug.

AEs will be graded as follows:

Mild: the patient is aware of the event, but can easily tolerate it;

Moderate: the patient experiences enough discomfort to interfere with normal activities;

Severe: the event is incapacitating and causes inability to perform normal activities.

Unexpected Adverse Reaction
An unexpected adverse reaction is an adverse reaction, the nature or severity of which is not consistent with the applicable Product Information (eg. The Investigator's brochure for an unauthorised investigational product or the Summary of Product Characteristics for an authorised product).

Ref. Directive 2001/20/EC, Directive 2001/83/EC, "Rules governing Medicinal products in the European Union, Vol. 9A – Guidelines on Pharmacovigilance for medicinal products for human use" – September 2008, ref: *Rules governing Medicinal products in China"*

10.1.5 Clinical Laboratory Abnormalities and Other Abnormal Assessments as AEs and SAEs

Abnormal laboratory findings (e.g. clinical chemistry, haematology, urinalysis) or other abnormal assessments (e.g. ECG, vital signs) that are judged by the Investigator as clinically significant will be recorded as AEs or SAEs if they meet the definition of an AE or SAE as previously defined.

Clinically significant abnormal laboratory findings or other abnormal assessments that are detected during the Study or are present at Screening and significantly worsen following the start of the Study will be reported as AEs or SAEs. However, clinically significant abnormal laboratory findings or other abnormal assessments that are associated with a disease reported in the medical history, unless judged by the Investigator as more severe than expected for the patient's condition, or that are present or detected at the start of the Study and do not worsen, will not be reported as AEs or SAEs.

The Investigator will exercise his/her medical and scientific judgement in deciding whether an abnormal laboratory finding or other abnormal assessment is clinically significant.

10.1.6 Monitoring of AEs

Patients will be monitored throughout the Study for adverse events to the Study formulation and/or procedures. AEs will be documented and collected on an ongoing basis during the Treatment Period and the relevant follow up period. The Investigator will instruct the patient how to communicate any AEs occurred in this period. Medical events fulfilling one or more criteria of seriousness and occurring after the informed consent has been signed, must be immediately (within maximum 24 hours) reported to the Sponsor using a SAE form, even if the Study treatment administration has not been started yet. These events must also be recorded on the "Medical/Surgical History" pages of the CRF (but not in the AE page), if the event occurred before the initiation of the Study Drug Treatment, or in the CRF AE page, if the Study Drug intake was already started.

Any patient who experiences an adverse event (AE) (whether serious or non-serious) or has a clinically significant abnormal laboratory test value(s) will be evaluated by the Investigator or his/her team and will be treated and/or followed up, even after the Study end, until the symptoms or value(s) return to normal, acceptable levels or chronicity, as judged by the Investigator or his/her team. Treatment of serious adverse events (SAEs) will be performed by a Physician. Where appropriate, medical test(s) and/or examination(s) will be performed to document resolution of event(s). All serious adverse events must be followed up till an outcome different from "on-going" will be reached.

10.1.7 Reporting of AEs/SAEs

Serious Adverse Events (SAE): should be immediately reported by telefax or by e-mail to **CVie Therapeutics Company Limited** or its designee by completed SAE Forms, at the Country Specific references reported on SAE form.

The Investigator should notify the **CVie Therapeutics Company Limited**, by telefax or by e-mail as soon as he/she is informed about the SAE, and in any case within 24 hours since the event discovery, even though only limited information is available.

When filling in the SAE Form, the Investigator may provide an Initial information only (he/she will subsequently give a Follow-Up/Final information), or even the final information in case all data, including outcome, are immediately available. The Investigator will promptly provide the **CVie Therapeutics Company Limited** , with the SAE Form appropriately filled in.
In accordance with local IEC (independent ethics committee) requirements, the Investigator must also notify IEC of any SAEs according the guidelines of the Ethics Committee.

In case of a patient's death, the Investigator should also supply the Sponsor and the Independent Ethics Committee with any additional requested information (e.g., autopsy reports and terminal medical reports).

Adverse Events: immediate reporting to the **CVie Therapeutics Company Limited** is not required and the Investigator when filling in the AE Form will describe the complete AE evolution till its outcome. The completed AE Form will be collected by the Study Monitor during the first Visit following the end of the event.
Should a AE become Serious, the Investigator will then follow the same reporting procedures as for SAEs.

10.1.8 Expeditable Events

Expeditable events are those adverse events that are causally related to the Study product and that are both serious and unexpected. Such events are subject to expedited reporting to regulatory authorities and will be reported within the stipulated timelines by the Sponsor or a suitably qualified designee. The Sponsor is responsible for reporting adverse events to the relevant governing authorities in the time frames and methodology applicable according to local law.

10.1.9 Overdose

Cases of overdose (accidental or intentional) which result in serious adverse reactions have to be handled following emergency procedures. These include reports related to drug intake with suicidal intentions and consequent drug overdose.

Should no adverse reaction occur, the information that overdosing occurred should be recorded in the “Comments” section of the CRF (placed at the end of the CRF), for its important safety and tolerability implications.

11. DIRECT ACCESS TO ORIGINAL DOCUMENTS

The Investigator/Institution shall allow National and foreign Regulatory Authorities, individuals delegated by the Independent Ethics Committee or the Sponsor to have direct access and related verification, to all Study original documentation, including forms of informed consents signed by the patients enrolled into the Study and clinic and/or outpatient records. Those having direct access to such documentation shall take the necessary reasonable precautions in order to keep identity of patients and information owned by the Sponsor confidential, in accordance with the relevant applicable legislation.

12. QUALITY CONTROL AND QUALITY ASSURANCE PROCEDURES

Organisation, monitoring and quality assurance of the present Clinical Trial is responsibility of **CVie Therapeutics Company Limited** that held the right to outsource some of the organisation and monitoring activities.

12.1. Steering Committee

A specific Steering Committee has been constituted in order to finalize the Study Protocol and address policy and operational issues related to the Protocol. The Steering Committee has the responsibility for protecting the scientific conduct and integrity of the trial. This Committee will meet periodically (even by teleconference) during the planning, implementation and conduct of the Study and will be under the direction of the two Committee Responsibles, one for each Country:

Prof. Jan A. Staessen- Chairman and Responsible for Italian patients
Studies Coordinating Centre
Laboratory of Hypertension
University of Leuven
Campus Sint Rafael,
Leuven – BELGIUM

Prof. Chern-En Chiang - co Chairman and Responsible for Taiwanese patients
Taipei Veterans General Hospital
201, Sec. 2, Shipai Rd., Beitou Dist.,
Taipei City 112, Taiwan

The Steering Committee will be constituted as follows:

Prof. Paolo Manunta
Div. di Nefrologia, Dialisi e Ipertensione
Ospedale San Raffaele
Via Olgettina, 60
20132 Milan - ITALY

Prof. Nicola Glorioso
Medicina d'Urgenza, Università di Sassari
Viale San Pietro, 8
07100 Sassari - ITALY

Prof. Daniele Maria Cusi
U.O. Nefrologia e Dialisi
Università degli Studi di Milano
Azienda Ospedaliera San Paolo
Via A. Di Rudinì, 8
20142 Milan - ITALY

Professor Jiguang Wang
Ruijin Hospital Affiliated to The
Shanghai Jiao Tong University
Medical School
No.197, Rui Jin Er Road,
Shanghai China

Professor Tzung-Dau Wang
National Taiwan University Hospital
No. 7, Zhong-Shan South Road,
Taipei City 10002, Taiwan

The main functions of this Committee include:

- Assistance of the Sponsor with the finalization of the Study Protocol and with the issuance of any amendment to the Study Protocol (this is considered necessary to enhance the scientific integrity and purpose of this Study).
- Development and submission of any and all manuscripts that are forthcoming from this Study and approval of all such formal publications. (All Investigators acknowledge that any publication of these trial results will be approved by the Steering Committee in advance of submission of any proposed publication.)
- Exclusion of patients from the per Protocol analysis, to be performed under blind condition.

12.2. Data and Safety Monitoring Board (DSMB)

A specific scientific and independent committee will be constituted before the starting of the Study in order to supervise the ethical performance of the Study and review safety data arising during the Study. The DSMB will be updated by the Sponsor through a formal report at regular intervals. Any adverse event which causes the termination of treatment or Serious Adverse Events experienced by the patients during the Study will be promptly reported to all members of the committee within 24 hours following time of their acknowledgement by the Sponsor. The committee has the authority to ask to open the code of a single patient in case of a Serious Adverse Event occurrence. The Committee determines whether the continuation of the Study is ethically justified for safety reason.

12.3 Case Report Form (CRF)

Recording of data will be based on an electronic CRF (eCRF). A Run-In and Treatment Period CRF will be prepared for the Study. The CRF will contain ad hoc forms for recording all variables mentioned in this Protocol and will be structured by Visit. The forms for registration of possible adverse events, concomitant medications and any suspension of the Study will be placed at the end of the CRF.

12.4 Clinical Monitoring

The task of the Monitor is to verify, according to the Sponsor's requirements, that the Study is conducted and documented properly in accordance with the Good Clinical Practice by carrying out contacts by phone and in person with the Principal Investigator and the clinical staff. These

Visits will enable the Monitor to maintain current, personal knowledge of the Study through review of the records, comparison with source documents, observation and discussion of the conduct of the Study with the Investigator. Indeed, the Monitor is responsible for furnishing the Investigator with Study materials, for verifying adherence to the Protocol, for completeness of the CRF, for verifying the respect of experimental requirements and for the relationship between the Investigator and the Sponsor. **CVie Therapeutics Company Limited** designed a C.R.O to perform all the monitoring activities foreseen for the Study. The Investigator agrees to receive regular Monitoring Visits if the enrolment rate is adequate, according to the Monitoring plan.

12.5 Audits

The Investigator/Institution shall allow the Sponsor to carry out audits, as an integral part of the quality assuring system. Audits are controls, independent and separate from monitoring, of Study activities and documents, aiming at verifying whether Study-related activities have been performed and data recorded, analysed and forwarded in compliance with Protocol, GCP, SOPs and applicable regulatory requirements. The Auditor has been appointed by the Sponsor to verify the conduct of the Study, having the possibility to perform audits at the investigational site with direct access to the Trial Master File, Case Report Forms, source documents, informed consent and patients' hospital records.

12.6 Inspections

The Investigator/Institution shall allow Regulatory Authorities, national and foreign, to carry out inspections.
Inspections, on part of one or more Regulatory Authorities, consist in an official revising of documents, structures, recordings and any other source said authorities consider as connected to the Clinical Study.

13. DATA MANAGEMENT

An electronic CRF (eCRF) to be filled in by the Investigator and/or his/her designee will be used. Clinical data management will be performed in accordance with applicable standards and data cleaning procedures with the objective of removing errors and inconsistencies in the data which would otherwise impact on the analysis and reporting objectives.
Medical History and Adverse Events will be coded using the MedDRA dictionary. Prior and concomitant medications will be coded using the WHO Drug dictionary.
After validation of the data (screening for internal coherence of recorded values, detection of abnormalities and discrepancies according to the plan of controls previously prepared), the database will be locked, the randomisation codes will be opened and the planned statistical analysis will be performed.
Any further modification of recorded data will be documented in a database log form.

14. STATISTICAL METHODS

All statistical analyses will be performed with SAS software (SAS Institute Inc.) version 9.2. The statistical analyses will be performed by the Biostatistics and Data Management Unit of CROS NT and detailed in the statistical analysis plan (SAP) which will be prepared by the same unit.

14.1 Sample Size Calculation

Sample size has been computed by assuming a standard deviation of 10 mmHg either at the overall sample level (**Genetic Profile 1**) and in the pre-defined subgroup bearing the **Genetic Profile 2**. This assumption is based on an evaluation coming from the previous Study on PST 2238.

The other assumption is that the **Genetic Profile 2** will comprise at least 50% of the total randomized patients.

Using these assumptions, a total sample size of 240 patients, i.e. 80 patients in the following groups (rostafuroxin 50, 500 micrograms and Losartan 50 mg), is suitable for discriminating a SBP mean difference of 4.5 mmHg between the group of the two highest doses of **ROSTAFUROXIN** and Losartan, with a standard deviation of 10mmHg, with a power of at least 80% and an alpha level of 0.05, two tailed test, in the **PP** population and in the ITT population assuming a 10% drop-out rate.

A total sample size of 120 patients (50% of the total sample), i.e. 40 patients in each group, is suitable for discriminating a SBP mean difference of 6.5 mmHg between the group of the two highest doses of **ROSTAFUROXIN** and Losartan, with a standard deviation of 10 mmHg a power of 80%, an alpha level of 0.05, two tailed test, in the **PP and ITT Genetic Profile 2** populations and assuming a 10% drop-out rate.

An interim blinded sample size re-assessment is planned and will be performed when approximately half of the patients (i.e. 120) will be randomized and will reach the final evaluation. All details of this interim analysis will be given in the statistical analysis plan.

<u>Interim blinded sample size re-assessment results</u>

A sample size re-assessment was performed to confirm the sample size requirement for this trial.

For this task the interim analysis was performed on 221 randomized patients having the final evaluation (i.e. if the patient answered "Yes" to the question "Did the patient complete the study treatment period?" reported in the "End study" CRF form).

The endpoint considered for the standard deviation estimation was the change in the office measurement of sitting blood pressure from baseline (Visit 3) to Visit 6. Missing data was not replaced.

The sample size re-assessment showed a higher variability compared to the assumptions of the Study Protocol. In addition to this, as discussed in detail in the attached rationale, the very fast development of personalized medicine requires the most appropriate translation of the CRT data to the individualized day to day clinical practice. This has prompted the Sponsor to add as a co-primary end point an individual assessment of efficacy which identifies the responders and non-responders in each treatment arm.

Responders are defined as "Patients having the mean SBP (mean of last three measurements) <= 135 mmHg or having a reduction in mean SBP (mean of the three last measurements) > or = 10% with respect to the baseline measurement" at visit 6 (after two months of therapy).

Considering the sample size foreseen in the Study Protocol and the percentages of responders seen in previous trials, the table below shows the effect of these assumptions on the power:

Genetic Profile	Total No. of evaluable subjects	Total number of randomised subjects	Alpha Level	Reference proportion	Proportions difference scenarios	Power
1	216	240 (80 per group)	0.025	35%	20%	0.708
					30%	0.978
					40%	>.999
2	105	120 (40 per group)	0.025	30%	20%	0.380
					30%	0.758
					40%	0.963

Considering the actual number of patients who completed the study in Italy, at least 102 patients should be reached in Taiwan to guarantee the levels of power included in the table above.

14.2 Patient Population to be Analysed

The following populations will be considered in the statistical analysis of efficacy:

- Safety population: all randomized patients that received at least one dose of the Study treatment.
- Intention-to-treat (ITT) population: total number of randomised patients having assumed at least one dose of Study Medication and having at least one evaluation in the Treatment Period. The LOCF (Last Observation Carried Forward) method will be used for replacing the missing data.
  For patients taking prohibited drugs (see sections 7.4), the assessments carried-out after prohibited drugs intake will be disregarded. In their place, the last available assessments before the administration of prohibited drugs will be taken into account for the statistical analysis and treated with the LOCF method.
  - ITT Genetic Profile 2 = patients of the previous population with Genetic Profile 2
  - Per Protocol (PP) population: all patients having fulfilled the eligibility criteria of the Protocol, performed the Visit at the end of the Treatment Period, completed the treatment with a compliance of at least 80% (see section 7.5), not having taken prohibited drugs during the course of the Study (see section 7.4) and not having discontinued the Study Drug treatment during the day before or the day of each scheduled Visit.

A patient interrupting the Study because of uncontrolled hypertension (i.e. office SBP ≥ 179 mmHg or DBP ≥ 110 mmHg) will contribute to the statistical analysis with his/her last blood pressure evaluation. Patients interrupting the Study for other reasons will not be included in the PP-analysis, but will be included in the ITT-analysis. Sample size of study population was

calculated considering a drop-out rate of 10%. In order to have enough patients included in PP- population, if during the study, when approximately 70% of patients reached the final evaluation, more than 10% have to be excluded from the PP-analysis, the number of patients to be randomized will be increased to reach the sample size.

14.3 Statistical Analysis

As for office SBP and DBP, the average of the last three readings will be considered in the statistical analysis. All the blood pressure variables are assumed to be normally distributed. All the variables will be descriptively analysed by Treatment and Visit (mean, standard deviation, minimum and maximum for continuous variables that are normally distributed; median and 25th and 75th percentiles for continuous variables that are asymmetrically distributed; frequency distribution for categorical variables). All the efficacy analyses will be applied in both the ITT and the PP populations, while the safety analyses will be carried-out in the Safety population. Results from the PP population will be considered the primary ones.

14.3.1 Efficacy Primary End-Points

Two co-primary endpoints have been defined for this study following the blinded interim sample size re-assessment (see Section 14.1). The two co-primary endpoints will be analysed as described below.

<u>The change from baseline to Visit 6 in office sitting SBP</u>
An analysis of covariance (ANCOVA) model will be used for analysing these data, with the changes from Baseline to Visit 6 as the outcome variable and terms for Treatment, country and interaction between these two as the explicative factors, and baseline as covariate.
In order to demonstrate that the two highest doses of Rostafuroxin are able to show a statistically significant difference on reduction of office sitting systolic blood pressure in comparison to the group of patients treated with Losartan 50 mg, the appropriate contrast will be carried-out within this ANCOVA model. This analysis will be performed in the total population bearing a mutation included in the **Genetic Profile 1** or the subgroup of patients showing the **Genetic Profile 2**.

<u>The proportions of responders at Visit 6</u>
The proportion of responders (as defined in Section 5) in each treatment group will be presented. A comparison between treatment groups on the proportions of responders will be performed using the Mantel-Haenszel test stratified by country. This analysis will be performed in the total population bearing a mutation included in the **Genetic Profile 1** or the subgroup of patients showing the **Genetic Profile 2**.

<u>Multiple testing issues</u>
Hypotheses concerning the two Genetic profiles will be tested in a hierarchical order for the primary endpoints. The hypotheses (H_1 and H_2) related to the **Genetic profile 2** will be tested first. The alpha level will be adjusted to take into account the two co-primary endpoints, linked by the “OR” condition. Therefore, the alpha level will be set to 0.025.
The same hypotheses (H_3 and H_4) will be tested in the **Genetic profile 1** only if at least one of H_1 or H_2 are rejected.

Further details of multiplicity adjustment procedure and alpha propagation method will be provided in the SAP.

14.3.2 Efficacy Secondary End-Points

An additional analysis will be performed for the two primary objectives by including the level of sodium at Screening as an extra explicative factor in the ANCOVA model above described. This analysis will be considered secondary.

No correction factor will be considered for the number of tests performed as secondary analyses. The p-values will have a descriptive nature and will be judged against the standard threshold of 0.05.

The comparison described in the previous section will also be performed with reference to the subgroup population bearing a mutation included in the **Genetic Profile 3.** Additional comparisons will be carried-out, as described in the previous section (see paragraph 4, Secondary objectives) (i.e. within the same ANCOVA model), in order to compare the three doses of Rostafuroxin to one another and to compare each individual dose versus Losartan.

The OBP measurement of sitting DBP will be analysed as previously described for the sitting SBP.

As for the 24 hours measurements of both SBP and DBP, within subjects, the ambulatory measurements will be averaged with weights according to the time interval between successive readings. Day-time and night-time will be defined on the basis of short fixed-clocktime intervals, which range from 10 AM to 8 PM and from midnight to 6 AM, respectively.

14.3.3 Safety Analysis

The Safety population will be used to evaluate safety and tolerability data.

Physical examinations, ECGs, vital signs, laboratory tests, adverse events and concomitant medications will be considered for the safety and tolerability evaluation.

Categorical variables (ECG normality, physical examination normality, etc.) will be analysed with shift tables (Baseline vs. final Visit), while continuous variables (laboratory parameters, vital signs, etc.) by descriptive summaries.

Laboratory data will also be analysed with shift tables (Baseline vs. Final Visit), considering each value as being normal/abnormal with respect to the appropriate normal ranges. Normal-abnormal shift tables will also be presented for physical examination results.

Different units of measure will be converted into SI units. Afterwards, for each laboratory test, observed values will be normalised with respect to a unique reference range.

In order to facilitate identification of outliers and medical interpretation of results, normalised laboratory values will be graphically represented by means of plots of Baseline vs. Final Assessments.

Adverse events will be coded using the MedDRA dictionary. Adverse events and all related information will be listed by patient. Descriptive statistics will be performed stratifying the events by system organ class and preferred term; they will also be stratified by seriousness and relationship with the Study Treatment.

Prior and concomitant medications will be summarised using the WHO coding system. Prior and concurrent medical conditions will be coded using the MedDRA dictionary.

15 ETHICS

All the parts involved in the Study agree upon and shall verify that current Study is carried out in compliance with the ethical principles arising from Helsinki Declaration (Appendix I), guide-lines of the Good Clinical Practice (GCP) and applicable regulatory requirements.

15.1 Ethical and Regulatory Authorisations

A clinical trial may be initiated only after receiving written approval by the Independent Ethics Committee (IEC) to which the Investigator Site must make reference to. The Investigator must therefore receive the favourable opinion of the IEC of the facility where the clinical trial will be conducted before beginning to enrol any patients into the study. The Sponsor and the Investigator must submit all the necessary documents required for attainment of the approval to the IEC. A copy of the approval by the IEC must be available at the Sponsor's facility before the initiation of the study.

This study must be submitted to the authorization of the Regulatory and Local Health Authorities in accordance with existing laws and regulations. The study cannot be initiated until a copy of the document of authorization has been obtained from Regulatory and Local Health Authorities as required by the applicable legislation. This documentation must be available at the Sponsor's facility before the investigational medicinal product is shipped and before the enrolment of any patients.

15.2 Informed Consent

Prior to Study beginning, written information to give the patients and Informed Consent forms (one for the Clinical Study and another for the authorization for genotype analysis), shall be submitted to examination and approval on part of the local Independent Ethical Committee, concomitantly with the Protocol.
An Informed Consent shall be required, obtained and documented by the Investigator in compliance with the applicable regulatory requirements, GCP and ethical principles originating from Helsinki Declaration.
(For details related to procedures to follow when applying and obtaining the Informed Consent to describe in the Protocol, see ICH-GCP E6, paragraphs 4.3.3, 4.3.4, 4.8.2, 4.8.3, 4.8.4, 4.8.5, 4.8.6, 4.8.7, 4.8.11, 8.3.2, 8.3.11 and as to procedures related to requests for Informed Consent in particular situations, see also the following paragraphs 4.8.12, 4.8.15, 4.8.13, 4.8.14, 4.8.9).

16. ADMINISTRATIVE PROCEDURES

16.1 Changes in the Conduct of the Study or Planned Analyses

Once the final Clinical Protocol has been issued and signed by the Investigator and the authorised signatories, it must not be informally altered. Clinical Protocol amendments are alterations to a legal document (the Clinical Protocol) and have the same legal status and must pass through the appropriate steps before being implemented. In general, any change must be approved by the IEC prior to be effective. Administrative changes need only notification to the IEC without approval. Any subsequent amendments must be made on separate sheet and must pass through the approval process. It must be clear to the Investigator that he/she may not change the Clinical Protocol without prior discussion with Sponsor, which should give its approval.

Completed and signed Clinical Protocol amendments will be circulated to all those who were on the circulation list for the original Clinical Protocol.
The original signed copy will be kept in the Study Trial Master File with the original Clinical Protocol.
It should be noted that where an amendment to the Protocol substantially alters the Study design or the potential risks to the patients, each patient's consent to continue participation should again be obtained. In order to obtain this new informed consent, an appropriate information sheet and a new informed consent form will be prepared.

16.2 Suspension/Interruption of the Study

Should the trial be prematurely terminated or suspended for any reason, the Regulatory Authorities and the Independent Ethic Committee will be promptly informed in written by the Investigator or by the Sponsor, according to the Regulatory requirements and the procedures detailed in the Good Clinical Practice (ICH-E6).
The Investigator will promptly inform the trial patients, assuring appropriate therapy and follow-up for all of them.

16.3 Archiving

The Investigator/Institution must ensure the archiving of the essential documents of the study as specified by GCP and in compliance with *applicable legislation*. The Investigator/Institution must adopt all the necessary measures to avoid accidental or premature destruction.

The Investigator/Institution must store the specific essential documents for at least 2 years after the latest approval of an application for a marketing authorization and until there are no on-going or foreseen applications for marketing authorizations, or until at least 2 years have passed from the formal interruption of the clinical development of the investigational medicinal product, and in any case, until the Sponsor has made a written notification that it is no longer necessary to maintain these documents. Nonetheless, these documents must be maintained for longer periods of time if so requested by relevant *applicable legislation* or by a specific agreement with the Sponsor.

16.4 Use of the Information of the Publication of the Results

The Investigator recognizes that all the information provided by **CVie Therapeutics Company Limited**, which has not been made available to the public, in regard to the investigational medicinal product (indication, patents, chemical formula, synthesis and formulation processes, study data or other information) is the property of **CVie Therapeutics Company Limited** and are strictly confidential. The Investigator may utilize this information exclusively for the conduct of the research.
In regard to the data derived from this clinical research, the Investigator is obliged to provide all the results obtained to the Sponsor.
Should the Investigator intend to divulge any of the results of the study, except for the notification of the adverse events foreseen by current legislation in regard to Pharmacovigilance, he/she must communicate this intention beforehand to **CVie Therapeutics Company Limited,** which must answer the request of the Investigator within two months of the date of receipt of the request.

CVie Therapeutics Company Limited will use the data derived from the clinical study in connection with the development of the drug and therefore may transmit this information, if necessary, to other Investigators and/or Regulatory Authorities.

16.5 Insurance Covering Public Liability only for Taiwan

This Clinical Trial is covered by insurance for legal liability for third person, undersigned by the Sponsor, with which the insurance company is responsible for the amounts owed by **CVie Therapeutics Company Limited** being civil responsible as requested by the law, for claim damage (capital, interest and expenses) for every damage caused by speciality and medical products, registered or not, administered during clinical trials.
Such guarantee is intended during a clinical trial:

- for doctor's or monitor's responsibilities that may derive during the experiments made by request and/or in behalf of the insured party;
- for any responsibilities for which the insured party is obliged by the law, rules, inner rules, custom or uses.

Are excluded:

- Damages to the health or worsening of the health which would have arrived also without the clinical trial;
- Genetic damages;
- Damages due to voluntary unfulfilled of prescriptions or instructions;
- Corporal damages due to A.I.D.S.;
- Damages due to incompetence or severe negligence.

Insurance of liability, arising under the law the Sponsor, the Monitor, the Investigator and his/her collaborators (only for Italy)

The sponsor declares to have an appropriate insurance policy, signed with a main Insurance Company, dedicated to the present study, in compliance with current regulations, with particular reference to the minimum requirements of the insurance policies, set forth in the Ministerial Decree 14/07/2009, for the protection of subjects participating in clinical trials of medicinals.

16.6 Financial Aspects

The financial aspects of this Study will be described in a "Financial Agreement", to be separately subscribed, between **CVie Therapeutics Company Limited** or a delegated party and the Institution(s) involved in the Study.

17. INVESTIGATOR'S RESPONSIBILITY

The Investigator is aware of his/her responsibility towards the Sponsor for all the actions delegated by him/her to other members of his/her staff assigned to the conduct of the study. Except where specifically required, the wording "Investigator" used in this protocol and in the CRF refers to the Investigator or to the qualified person designated by him/her who may carry out activities relevant to the clinical trial and sign the study documents on his/her behalf.
The Investigator is obliged to conduct the study in compliance with the study protocol and in adherence to Good Clinical Practice (ICH-E6) and with the principles of the Declaration of

Helsinki (1964) and successive revisions (Appendix I) as well as in respect of applicable legislation.

18. FINAL STUDY REPORT

The Final Clinical Report will be written by the Investigator or by the Sponsor and then approved by the Investigator. It is structured as an Integrated Clinical Report containing clinical comments based on the data generated by the Statistical Report.

19. REFERENCES

1. Swales JD: Current clinical practice in hypertension: the EISBERG (Evaluation and Interventions for Systolic Blood Pressure Elevation – Regional and Global) project. Am Heart J 1999; 138: S232-S237.

2. Kearney PM, Whelton M, Reynolds K, Muntner P, Whelton PK, He J.: Global burden of hypertension: analysis of worldwide data. *Lancet* 2005; 365, 217-23.

3. European Society of hypertension – European society of cardiology guidelines. 2003 European society of cardiology guidelines for the management of arterial hypertension. J Hypertens 2003; 21: 1011-1053.

4. Chobanian AV, Bakris GL, Black HR et al.: Blood Institute Joint National Committee on Prevention, Detection, Evaluation, and Treatment of High Blood Pressure, National High Blood Pressure Education Program Coordinating Committee. The seventh report of the joint national committee on prevention, detection, evaluation, and treatment of high blood pressure. The JNC 7 Report. JAMA 2003; 289: 2560-2572.

5. Prise en charge des patients atteints d'hypertension artérielle essentielle. Actualization 2005: Haute Autorité de la Santé.

6. Blood Pressure Lowering Treatment Trialists' Collaboration. Effects of different blood-pressure-lowering regimens on major cardiovascular events: results of prospectively-designed overviews of randomised trials. Lancet 2003; 362:1527-1535.

7. Lawes CMM, Vander Hoorn S, Law MR, Elliot P, MacMahon S, Rodgers A. Blood pressure and the global burden of disease 2000. Part II: estimates of attributable burden. J Hypertens 2006; 24:423-430.

8. Degli Esposti L, Valpiani G. Pharmacoeconomic burden of under treating hypertension. Pharmacoeconomics 2004; 22:907-928.

9. Kearney PM, Whelton M, Reynolds K, Munter P, Whelton PK, He J. Global burden of hypertension: analysis of worldwide data. Lancet 2005; 365:217-223.

10. Gaziano TA, Bitton A, Anand S, Weinstein C. The global cost of nonoptimal blood pressure. J Hypertens 2009; 27:1472-1477.

11. Muller-Nordhorn J, WIllich SN. Angiotensin II antagonists in the treatment of hypertension – effective and efficient? Herz 2003; 28: 733-737.

12. Moore MA. Drugs that interrupt the renin-angiotensin system should be among the preferred initial drugs to treat hypertension. J Clin Hypertens 2003; 5: 137-144.

13. Sleight P, Yusuf S. New incidence on the importance of the rennin-angiotensin system in the treatment of higher-risk patients with hypertension. J Hypertens 2003; 21: 1599-1608.

14. McIntyre M, Caffe SE, Michalak RA, Reid JL. Losartan, an orally active angiotensin (AT1) receptor antagonist: a review of its efficacy and safety in essential hypertension. Pharmacol Ther 1997; 74: 181-194.

15. Lindenfeld J, Borer J, Armstrong PW. Losartan potassium (Cozaar). Circulation 2002; 105: e9100.

16. Podzolkow VI, Bulatov VA, Son EA, Os I. Central and peripheral haemodynamic effects of losartan and combination with hydrochlorothiazide in mild to moderate essential hypertension. Blood Press 2003; 12: 239-245.

17. Messerli FH. Effects of losartan in hypertension without vascular disease. Ann Intern Med 2004; 140: 169-177.

18. Verma A, Ventura HO. Cardiovascular outcomes and angiotensin converting enzyme inhibitors: beyond blood pressure control. Cardiovasc Drug Ther 2009; 23:109-111.

19. Shih PA, O'Connor. Hereditary determinants of human hypertension. Strategies in the setting of genetic complexity. Hypertension 2008; 51 (6): 1456-1564

20. Levy D, et al. Genome-wide association study of blood pressure and hypertension. Nat Genet 2009; 41 (6): 677-687.

21. Newton-Cheh C, et al. Genome-wide association study identifies eight loci associated with blood pressure. Nat Genet 2009; 41 (6): 666-676.

22. Zanchetti A, Mancia G. The dilemma of placebo controlled studies: scientific evidence, guidelines, ethics and regulatory recommendations. J Hypertens 2009; 27: 1-2.

23. Boissel JP, Collet JP, Lion L, Ducruet T, Moleur P, Luciani J et al. A randomized comparison of the effect of four antihypertensive monotherapies on the subjective quality of life in previously untreated asymptomatic patients: field trial in general practice. J Hypertens 1995; 13:1059-1067.

24. Sciarrone MT, Stella P, Barlassina C, Manunta P, Lanzani C, Bianchi G, Cusi D. ACE and α-adducin polymorphism as markers of individual response to diuretic therapy. Hypertension 2003; 41:398-403.

25. Bianchi G, Tenconi LT, Lucca R. Effect in the consciuous dog of constriction of the renal artery to a sole remaining kidney on haemodynamics, sodium balance, body fluid volumes, plasma renin concentration and pressor responsivness to angiotensin. Clin Sci 1970; 38:741-766.

26. Guyton AC, Hall JE, Coleman TG, Manning RD Jr, Norman RA Jr. The dominant role of the kidneys in long-term arterial pressure regulation in normal and antihypertensive states. Hypertension Pathophysiology, Diagnosis and Management (Vol.1). Laragh JH, Brenner BM (Eds), Raven Press Publishers, New York, USA 1995: 1311-1326.

27. Guyton AC. Dominant role of the kidneys and accessory role of whole-body autoregulation in the pathogenesis of hypertension. Am J Hypertens 1989; 2:575-585.

28. Watkins BE, Davis JM, Freeman RH, De Forrest JM, Sthephens GA. Continuous angiotensin II blockade throughout the acute phase of one-kidney hypertension in dog. Circ Res 1978; 45:813-821.

29. Bing RF, Russell GI, Swales JD, Thurston H. Effects of 12-hour infusions of saralasin or captopril on blood pressure in hypertensive conscious rats. Relationship to plasma rennin, duration of hypertension, and effect of unclipping. J Lab Clin Med 1981; 98:302-310.

30. Freeman RH, Davis JO, Watkins BE, Stephens GA, De Forrest JM. Effects of continuous converting enzyme blockade on renovascular hypertension in the rat. Am J Physiol 1979; 236:F21-F24.

31. Edmunds ME, Russel GI, Bing RF. Reversal of experimental renovascular hypertension. J Hypertens 1991; 9:289-301.

32. Oelkers W, Diederich S. Primary hyperaldosteronism without suppressed rennin due to secondary hypertensive kidney damage. J Clin Endocrinol Metab 2000; 85:3266-3270.

33. Mulvany MJ. Structural changes in the resistance vessels in human hypertension. In: Hypertension Pathophysiology, Diagnosis and Management (Vol.1). Laragh JH, Brenner BM (Eds), Raven Press Publishers, New York, USA 1995: 503-513.

34. Stowasser M. Hyperaldosteronism: primary versus tertiary. J Hypertens 2002; 20:17-19.

35. Scriver CR, Waters PJ. Monogenic traits are not simple lessons from phenylketonuria. Trends Genet 1999; 15:267-272.

36. Wheatherall DJ. Phenotype-genotype relationship in monogenic disease: lessons from the thalassaemias. Nat Rev Genet 2001; 38:198-203.

37. “OASIS” trial report PST2238 DM 03 010 Study: a double blind, dose-range, placebo controlled study of the effects of PST2238 vs placebo in patients with stable, uncomplicated, essential hypertension. Sigma Tau data on file.

38. Lanzani C, Citterio L, Glorioso N, et al. Adducin- and ouabain-related gene variants predict the antihypertensive activity of rostafuroxin, part 2: clinical studies. SciTransl Med 2010; 2: 59ra87

39. Ferrandi M, Molinari I, Torielli L, Padoani G, Salardi S, Rastaldi MP, Ferrari P, Bianchi G, Adducin- and ouabain-related gene variants predict the antihypertensive activity of rostafuroxin. Part 1: Experimental studies. Sci. Transl. Med. 2, 59ra86 (2010)

40. Bianchi G, Manunta P, Glorioso N. Clinical impact of adducin polymorphism. J Hypertens 2009; 27:1325-1327.

41. Staessen JA, Kuznetsova T, Acceto R, Bacchieri A, Brand E, Burnier M, et al. Ouabain and adducin for specific intervention on sodium in hypertension (OASIS-HT): design of a pharmacogenomic dose-finding study. Pharmacogenomics 2005; 6:755-775.

42. Bianchi G, Tripodi G, Casari G, Salardi S, Barber BR, Garcia R, Leoni P, Torielli L, Cusi D, Ferrandi M, et al. Two point mutations within the adducin genes are involved in blood pressure variation. *Proc Natl Acad Sci* 1994, 91, 3999-4003.

43. Tripodi G, Valtorta F, Torielli L, Chieregatti E, Salardi S, Trusolino L, Menegon A, Ferrari P, Marchisio PC, Bianchi G. Hypertension-associated point mutations in the adducin alpha and beta subunits affect actin cytoskeleton and ion transport. *J Clin Invest* 1996, 97, 2815-2822.

44. Cusi D, Barlassina C, Azzani T, Casari G, Citterio L, Devoto M, Glorioso N, Lanzani C, Manunta P, Righetti M, Rivera R, Stella P, Troffa C, Zagato L, Bianchi G. Polymorphisms of alpha-adducin and salt sensitivity in patients with essential hypertension. *Lancet* 1997, 349, 1353-1357.

45. Bianchi G. Genetic variations of tubular sodium reabsorption leading to “primary” hypertension: from gene polymorphism to clinical symptoms. *Am J Physiol Regul Integr Comp Physiol* 2005, 289, R1536-1549.

46. Ferrandi M, Molinari I, Barassi P, Minotti E, Bianchi G, Ferrari P. Organ hypertrophic signalling within caveolae membrane subdomains triggered by Ouabain and antagonized by PST 2238. *J Biol Chem*. 2004, 279, 33306-33314.

47. Manunta P, Ferrandi M, Bianchi G, Hamlyn JM. Endogenous Ouabain in cardiovascular function and disease. *J Hypertens.* 2009, 27, 9-18.

48. Quadri L., Bianchi G., Cerri A., Fedrizzi G., Ferrari P., Gobbini M., Melloni P., Sputore S., Torri M. 17b-(3-Furyl) 5b- androstane-3b,14b,17a-triol (PST2238). A very potent antihypertensive agent with a novel mechanism of action. J. Med. Chem. 1997, 40: 1561-1564.

49. Ferrari P., Ferrandi M., Tripodi G., Torielli L., Padoani G., Minotti E., Melloni P., Bianchi G. PST 2238: a new antihypertensive compound that modulates Na-KATPase in genetic hypertension. J. Pharm. Exp. Ther. 1999; 288: 1074-1083.

50. Ferrari P., Torielli L., Ferrandi M., Padoani G., Duzzi L., Florio M., Conti F., Melloni P., Vesci L., Corsico N., Bianchi G. PST 2238, a new antihypertensive compound which

antagonises the long-term pressor effect of Ouabain. J. Pharm. Exp. Ther. 1998, 285:83-94.

51. Ferrandi M., Barassi P., Minotti E., Duzzi L., Molinari I., Bianchi G., Ferrari P. PST 2238: a new antihypertesive compound that modulates renal Na-K pump function without diuretic activity in Milan hypertensive rats. J. Cardiovasc. Pharmacol. 2002; 40: 881-889.

52. Losse H, Zumkley H, and Quante T Side effects of diuretics. Clin Exp Hypertens Theory Pract 1983; A5(2):309-320.

53. Ames RP The effects of antihypertensive drugs on serum lipids and lipoproteins I Diuretics. Drugs 1986; 32: 260-278.

54. Murphy MB, Lewis PJ, Kohener E et al. Glucose intolerance in hypertensive patients treated with diuretics; a fourteen-year follow-up. Lancet 1982; 11: 1293-1295.

55. Darracott Vaughan E, Carey RM, Peach MJ et al. The renin response to diuretic therapy. A limitation of antihypertensive potential. Circ Res 1978; 42(3): 376-381.

56. Wenceslau C.F., Rossoni L.V. Rostafuroxin ameliorates endothelial dysfunction and oxidative stress in resistance arteries from deoxycorticosterone acetate-salt hypertensive rats: the role of Na-KATpase (cSRC pathway). J.Hypertens. 2014, 32(3): 24-29.

57. Ferrandi M, Molinari I, Rastaldi MP, Ferrari P, Bianchi G, Manunta P. Rostafuroxin Protects from Podocyte Injury and Proteinuria Induced by Adducin Genetic Variants and Ouabains. JPET 2014; 351: 278-287.

58. Prassis Research Institute Sigma Tau. Research Report N°: PST2238-CV/20: Effect of a long-term treatment with PST2238 on blood pressure and vascular contractility in ouabain hypertensive rats (OS). 3rd March 2003

59. Prassis Research Institute Sigma Tau. Research Report N°: PST2238-CV/22. Effect of a long term treatment with PST2238 on vascular contractility in Milan Hypertensive rats (MHS). March 2003

60. R.B.M. Istituto di Ricerche Biomediche "Antoine Marxer". Evaluation of haemodynamic effects following intravenous administration in the anaesthetised dog. Exp. No. 970186, 1997.

61. Sigma-Tau Research and Development - Toxicology Department Study of the evaluation of possible androgenic or estrogenic effect of the test article PST2238 administered by oral route to immature rat. Exp. No. 0697T, 1997

62. Navarra P. Relazione sull'attività scientifica svolta nell'ambito del programma di ricerche su "Valutazione del profilo endocrino della molecola PST2238". Università Cattolica del SacroCuore - Facoltà di Medicina e Chirurgia "Agostino Gemelli" - Istituto di Farmacologia, April 1997.

63. Sigma Tau Research and Development - Pharmacology Department - "In vitro" study of the effect of PST2238 on some hormonal steroid receptors. DOC DLF No. 123, 8 February 1993

64. R.B.M. Istituto di Ricerche Biomediche "Antoine Marxer". Evaluation of effect on the gastric secretion (Shay's method) in the rat following single oral administration. Exp. No. 970192, 1997

65. Prassis Research Institute Sigma Tau. Research Report N°: PST2238/10: Study on the inotropic effect of PST2238 administered by slow intravenous infusion, in the anaesthetised Guinea pig. 1 September 1997

66. Sigma Tau Research Direction - Toxicology Department. Acute toxicity study (limit test) in the rat of test article PST2238 administered by oral route at a single dose-Exp. no. 1196T. 1996.

67. Sigma Tau Research Direction - Toxicology Department. Acute toxicity study (limit test) in the mouse of test article PST2238 administered by oral route at a single dose-Exp. no. 1296T. 1996

68. RBM - Istituto di Ricerche Biomediche “Antoine Marxer”. 4-week repeated dose toxicity study in Sprague Dawley Crl:CD (SD) BR rats treated with the test article PST2238 administered by oral route at the doses of 0, 45, 180 and 720 mg/kg/day followed by 4 weeks of recovery - RBM Exp. No. 960066. 1997

69. RBM - Istituto di Ricerche Biomediche “Antoine Marxer”. 13-week repeated dose toxicity study in Sprague Dawley Crl:CD (SD) BR rats treated with the test article PST2238 administered by oral route at the doses of 0, 4, 20 and 100 mg/kg/day followed by 6 weeks of recovery. RBM exp. No. 960441. 1997

70. RBM - Istituto di Ricerche Biomediche “Antoine Marxer”. 4-week repeated dose toxicity study in Cynomolgus monkeys treated with the test article PST2238 administered by oral route at the doses of 0, 45, 180 and 720 mg/kg/day followed by 4 weeks of recovery - RBM Exp. No. 960065. 1996.

71. RBM - Istituto di Ricerche Biomediche “Antoine Marxer”. 13-week repeated dose toxicity study in Cynomolgus monkeys treated with the test article PST2238 administered by oral route at the doses of 0, 5, 30 and 180 mg/kg/day followed by 6 weeks of recovery - RBM Exp. No. 960520. 1997

72. T. Duvachelle, ASTER, Paris. Double blind, placebo-controlled tolerability and pharmacokinetics study of PST2238 after a one week repeated oral administration period in three parallel groups of 12 healthy male volunteers (Protocol PST2238/HS/98/1). Sigma-Tau file.

73. Citterio L., Bianchi G. and Manunta P., sironi M., Li Y., Wang J.G. CVie Therapeutics Limited Internal Report: Comparison between Caucasian and Chinese genetic profiles predicting the response to rostafuroxin in Chinese. 14 Oct. 2014

74. Hakes L, Pinney JW, Robertson DL, Lovell SC. Protein-protein interaction networks and biology what's the connection? Nat Biotechnol 2008; 26:69-72.76.

75. Sauer U, Zamboni N. From biomarkers to integrated network responses. Nat Biotech 2008; 26:1090-1092.

76. Gibson G. Decanalization and the origin of complex disease. Nat Rev Genet 2009; 10:134-140.

77. Erwin DH, Davidson EH. The evolution of hierarchical gene regulatory networks. Nat Rev Genet 2009; 10:141-148.

78. Liu ET. Integrative biology: a strategy for systems biomedicine. Nat Rev Genet 2009; 10:64-68.

79. Ryan C.L., CimermancˇicˇP. Szpiech Z.A., Sali A., Hernandez R.D., Krogan N.J. High resolution network biology: connecting sequence with function. Nat. Rev. Genet. 2013; 14: 865-879.

80. Le Novere N. Quantitative and logic modelling of molecular and gene networks. Nat. Rev. Genet. 2015; 16: 146-158.

81. Rechie M.D., Holzinger E.R., Li R., Pendergrass S.A., Kim D. Methods of integrating data to uncover genotype–phenotype interactions. Nat. Rev. Genet. 2015; 16: 85-97.

82. Pheasant M, Mattick JS. Raising the estimate of functional human sequences. Genome Res. 2007 Sep;17(9):1245-53.

83. Neilson JR, Sharp PA. Small RNA regulators of gene expression. Cell 2008; 134:899-902.

84. Ruvkun G. The perfect storm of tiny RNAs. Nat Med 2008; 14:1041-1045.

85. Fabian MR, Duchaine TF. Micro RNAs: the bench and beyond. Cell 2008; 135:587-588.

86. Mercer TR, Dinger ME, Mattick JS. Long non-coding RNAs: insights into functions. Nat Rev Genet 2009; 10:155-159.

87. Sethupathy P, Borel C, Gagnebin M, Grant GR, Deutsch S, Elton TS et al. Human microRNA-155 on chromosome 21 differentially interacts with its polymorphic target in the AGTR1 3' untranslated region: a mechanism for functional single-nucleotide polymorphisms related to phenotypes. Am J Hum Genet 2007; 81:405-413.

88. Martin MM, Buckenberger JA, Jiang J, Malama GE, Nuovo GJ Chotani M et al. The human angiotensin II type 1 reptor +1166 A/C polymorphism attenuates microma-155 binding. J Biol Chem 2007; 282:24262-24269.

89. Shao H, Burrage LC, Sinasac DS, Hill AE, Ernest SR, O'Brien W et al. Genetic architecture of complex traits: large phenotypic effects and pervasive epistasis. PNAS 2008; 105:19910-19914.

90. Wei WH, Hemani G., Haley C.S. Detecting epistasis in human complex traits. Nat- Rev. Genet. 2014; 15: 722-733.

91. Phillips PC. Epistasis the essential role of gene interactions in the structure and evolution of genetic systems. Nar Rev Genet 2008; 9:855-867.

92. Chakavarti A., Clark A.G., Mootha V.K. Distilling pathophysiology from complex disease genetics. Cell 2013; 155: 21- 26.

93. Marian J. Causality in genetics.The gradient of genetic effects and Back to Koch's postulates of causality. Circ. Res. 2014; 114:e18-e21.

94. Vallance P, Levick M. Drug discovery and development in the age of molecular medicine. Clin Pharmacol Ther 2007; 82:363-366.

95. Araujo RP, Liotta LA, Petricoin EF. Proteins, drug targets and the mechanisms they control: the simple truth about complex networks. Nat Rev Drug Discov 2007; 6:871-880.

96. Lehár J, Stockwell BR, Giaever G, Nislow C. Combination chemical genetics. Nat Chem Biol 2008; 4:674-681.

97. Hopkins AL. Network pharmacology: the next paradigm in drug discovery. Nat Chem Biol 2008; 4:682-690.

98. Russel RB, Aloy P. Targeting and tinkering with interaction networks. Nat Chem Biol 2008; 4:666-673.

99. Harrison C. Identifying drug-genotype interactions. Nat Rev Drug Discov 2009; 8:109.

100. Roses AD. Pharmacogenetics in drug discovery and development: a translational perspective. Nat Rev Drug Discov 2008; 7:807-817.

101. Citterio L., Salvi E., Lanzani C., Macciardi F., Bianchi G. CVie Therapeutics Company Internal Report: OASIS: Pharmacogenomic investigation of hypertensive patients using a Genome Wide Association Study strategy. 23 April, 2009.

102. Quing Chen et Al. Association between Ambient Temperature and Blood Pressure and Blood Pressure Regulators: 1831 Hypertensive Patients Followed Up for Three Years, Plos One-open access.Published: December 31, 2013

103. Modesti PA et Al Seasonal blood pressure changes: an independent relationship with temperature and daylight hours Hypertension. 2013 Apr;61(4):908-14.

104. Modesti PA, Season temperature and blood pressure: a complex interaction. European J.of Internal Med. 2013,24:604-607

105. Modesti PA. Seasonal blood pressure changes: which ambient temperature should we consider; J. of Hypertension. 2014,32:1577-1579

106. Yu-Kang Tu et Al. Seasonal variation in blood pressure is modulated by gender and age but not by BMI in a large Taiwanese population 1996-2006. JASH http://dx.doi.org/10.1016/j.jash.2013.01.008

107. Weber M.A.a, Schiffrin E.L.,White W.B., Mann S., Lindholm L.H., Kenerson J.G., Flack J.M., Carter B.L., Materson B.J., Ram C.V.S, Cohen D.L., Cadet J.C, Jean-Charles R.R., Taler S., Kountz D., Townsend R, Chalmersq J, Ramirezr A.J., Bakriss G.L., Wangt J., Schutteu A.E., Bisognanov J.D., Touyzw R.M., Sicax D., Stephen B. Harrap S.B. Clinical Practice Guidelines for the Management of Hypertension in the Community. A Statement by the American Society of Hypertension and the International Society of Hypertension Society of Cardiology (ESC). J. Hypert. 2013, 31:

1. APPENDIX I - WORLD MEDICAL ASSOCIATION DECLARATION OF HELSINKI

http://www.wma.net/en/30publications/10policies/b3/17c.pdf
