# Supplementary material for: Antihypertensive treatment guided by genetics: PEARL-HT, the randomized proof-of-concept trial comparing rostafuroxin with losartan
Source: Pharmacogenomics J. 2021 Mar 1;21(3):346–58. doi: 10.1038/s41397-021-00214-y (PMC8159753; doi:10.1038/s41397-021-00214-y)
Supplement: Supplementary file 4 — Text S2 [file 41397_2021_214_MOESM4_ESM.pdf]

STATISTICAL ANALYSIS PLAN

PROTOCOL: PST 2238-DM-10-001 (Italy) / CVT-CV-001 (Taiwan)

ANTIHYPERTENSIVE EFFECT OF DIFFERENT DOSES OF ROSTAFUROXIN IN COMPARISON WITH LOSARTAN, ASSESSED BY OFFICE AND AMBULATORY BLOOD PRESSURE MONITORING IN A HYPERTENSIVE POPULATION SELECTED ACCORDING TO A SPECIFIC GENETIC PROFILE

STATISTICAL ANALYSIS PLAN

APPROVAL PAGE

Document Information	
Protocol Number:	PST 2238-DM-10-001 (Italy) / CVT-CV-001 (Taiwan)
Version:	Final 2.0
Document Date:	20 April 2018
Prepared for:	CVie Therapeutic Company Limited
Prepared by:	Valeria Bandiera, CROS NT

The Statistical Analysis Plan has been completed and reviewed and the contents are approved for use for the analysis.

Lead Statistician details:	
Name:	Valeria Bandiera
Job Role:	Senior Biostatistician
Company:	CROS NT
Signature:	Valeria Bandiera
Date of signature:	20-APR-2018 *(DD Mmm YYYY)*

Sponsor Approver details:	
Name:	Giuseppe Bianchi
Job Role:	Appointed Medical Expert
Company:	CVie Therapeutics Company Limited
Signature:	
Date of signature:	*(DD Mmm YYYY)*

STATISTICAL ANALYSIS PLAN

APPROVAL PAGE

Document Information	
Protocol Number:	PST 2238-DM-10-001 (Italy) / CVT-CV-001 (Taiwan)
Version:	Final 2.0
Document Date:	20 April 2018
Prepared for:	CVie Therapeutic Company Limited
Prepared by:	Valeria Bandiera, CROS NT

The Statistical Analysis Plan has been completed and reviewed and the contents are approved for use for the analysis.

Lead Statistician details:	
Name:	Valeria Bandiera
Job Role:	Senior Biostatistician
Company:	CROS NT
Signature:	
Date of signature:	*(DD Mmm YYYY)*

Sponsor Approver details:	
Name:	Giuseppe Bianchi
Job Role:	Appointed Medical Expert
Company:	CVie Therapeutics Company Limited
Signature:	[signature]
Date of signature:	20 April 2018 *(DD Mmm YYYY)*

Contents

Abbreviations 5
Revision History 6
1. Introduction 6
2. Study Objectives 6
  2.1 Primary objectives 7
  2.2 Secondary objectives 7
3. Study Design 8
  3.1 General design and plan 8
  3.2 Visit Schedule and Visit Windows 8
  3.3 Sample size justification 9
  3.4 Randomization and blinding 11
  3.5 Efficacy endpoints 11
    3.5.1 Primary endpoints 11
    3.5.2 Secondary efficacy endpoints 12
    3.5.3 Exploratory efficacy endpoints 12
  3.6 Safety endpoints 12
4. Statistical Analysis 12
  4.1 General 12
  4.2 Analysis sets 13
    4.2.1 Full analysis set (FAS) 13
    4.2.2 Per Protocol set (PPS) 13
    4.2.3 Safety set 13
    4.2.4 Screened set 14
    4.2.5 Randomized set 14
    4.2.6 Incorrect treatment allocations 14
  4.3 Sub-group analyses 14
  4.4 Covariates 14
  4.5 Pooling of sites 15
  4.6 Interim analyses 15
  4.7 Handling of missing and incomplete data 16
  4.8 Changes in the planned analysis 17
  4.9 Data Review Meeting 17
  4.10 Software 17
5. Evaluation of Demographic and Baseline Characteristics 17
  5.1 Subject enrolment and disposition 18
  5.2 Protocol violations 18
  5.3 Study discontinuations 18

5.4 Demographics and baseline characteristics 18
5.5 Medical and surgical history 19
5.6 Prior and concomitant medications 19
6. Evaluation of Treatment Compliance and Exposure 20
6.1 Compliance to study drug and treatment 20
6.2 Exposure to study drug 22
7. Evaluation of Efficacy 22
7.1 Analysis of primary endpoint 22
7.2 Analysis of secondary efficacy endpoints 26
8. Evaluation of Safety 28
8.1 Adverse events 29
8.2 Clinical laboratory evaluation 30
8.3 Vital signs 30
8.4 ECGs 30
8.5 Physical examination 30
Significant physical abnormality will be presented in the listings only. 30
9. Tables, Figures and Listings 30
9.1 Programs and tables quality control 30
9.2 Programming conventions 31
9.3 Tables 32
9.4 Figures 39
9.5 Listings 39
10. Literature 40
11. Appendices 41
11.1 Table shells 41
11.2 Figure shells 41
11.3 Listing shells 41
Figure shells not applicable for this study. 41

Abbreviations

ADR	Adverse Drug Reaction
AE	Adverse Event
ANCOVA	Analysis of Covariance
ATC	Anatomical Therapeutic Chemical
BMI	Body Mass Index
CEU	Caucasian
CH	Chinese
CI	Confidence Interval
CRF	Case Report Form
DBP	Diastolic Blood Pressure
DMP	Data Management Plan
ECG	Electrocardiogram
FAS	Full Analysis Set
ITT	Intent to Treat
LOCF	Last Observation Carried Forward
LSS	Lanosterol Syntase
EO	Endogenous Ouabain
EoT	End of Treatment
mcg	Micrograms
MedDRA	Medical Dictionary for Regulatory Activities
OBP	Office Blood Pressure
PP	Per Protocol
PT	Preferred Term
SAE	Serious Adverse Event
SAP	Statistical Analysis Plan
SAS	Statistical Analysis System
SBP	Systolic Blood Pressure
SD	Standard Deviation
SOC	System Organ Class
SOP	Standard Operating Procedure
TEAE	Treatment-Emergent Adverse Event
WHO-DD	WHO Drug Dictionary

Revision History

Document Version	Changes Made	Document Date
Draft 1.0	Draft specification based on the following documents:<br>• Study Protocol (Final Version, 25 June 2015);<br>• CRF (Final Version, 23 November 2012);<br>• Specification for Holter's data (Final version, 17 January 2014);	17 June 2015
Draft 2.0	Draft specification based on new version of Study Protocol (Final Version, 11July 2017)	8 November 2017
Draft 3.0	Final version based on Sponsor's review	12 December 2017
Final 1.0	Final version based on Sponsor's review	15 January 2018
Draft 1.1	Draft specification based on the following documents:<br>• Data Review Report (Version 1.0, 20 April 2018)	19 April 2018
Draft 1.2	Draft specification with revised number of optional tables as per Sponsor's request	19 April 2018
Final 2.0	Final version confirmed after the Sponsor review	20 April 2018

1. Introduction

This document outlines the statistical methods to be implemented in the analysis of the data of PST 2238-DM-10-001 (Italy) / CVT-CV-001 (Taiwan) Clinical Trial. The purpose of this plan is to provide general guidelines from which the analysis will proceed, containing a more technical and detailed elaboration of the principal features of the analysis described in the protocol. Any changes to the protocol or Case Report Form (CRF) may necessitate updates to the Statistical Analysis Plan (SAP). In case of deviations from this updated SAP, explanations will be provided in the clinical study report.
All final study data will be considered for the analysis regulated by this SAP.
The data to be included in the interim analysis are specified in the paragraph no. 4.6.

2. Study Objectives

The aim of the present study is to demonstrate, in a prospective way, that Rostafuroxin activity in reducing blood pressure levels in never treated patients, bearing mutations in genes encoding for adducin proteins (ADD1, ADD2, ADD3) and the enzymes involved in Endogenous Ouabain (EO)

synthesis, is higher as compared to the activity of the standard treatment with a recognized effective drug like Losartan.

2.1 Primary objectives

The two primary objectives are:
to demonstrate that the highest two doses of Rostafuroxin are able to show a statistically significant difference on reduction of office sitting systolic blood pressure at Visit 6 (measured either as change from baseline or proportions of responders at Visit 6) in comparison to the group of patients treated with Losartan 50 mg in either:

- the total population bearing a mutation included in the Genetic Profile 2, or
- the subset of patients of the total population carrying only the Genetic Profile 1.

2.2 Secondary objectives

The secondary objectives of this study are:

- According to the clinic blood pressure evaluations:
    - to demonstrate that the highest two doses of Rostafuroxin are able to show a statistically significant difference on reduction of office sitting systolic blood pressure in comparison to the group of patients treated with Losartan 50 mg in the subgroup of patients of the total population carrying only the Genetic Profile 3.
    - to demonstrate that the two highest doses of Rostafuroxin are able to show a statistically significant difference on office sitting diastolic blood pressure in comparison to Losartan in the general population and/or in the two pre-defined subgroups of patients;
    - to compare one another the three doses of Rostafuroxin and each one versus Losartan;
    - to determine a dose/responder profile, if any;
    - to evaluate the effect of variation of outside temperature on Blood Pressure on the different type of BP levels and measurements
- According to the 24h-Ambulatory Blood Pressure Monitoring:
    - to identify the oral doses of Rostafuroxin which lead to statistically significant differences in daytime, night-time and overall 24-hour ambulatory systolic blood pressure and/or diastolic blood pressure, in comparison with Losartan in the general population and/or in the subgroup populations, this analysis will be first run with the two highest doses of Rostafuroxin pulled together versus Losartan and then each single dose of Rostafuroxin will be compared vs. Losartan.
- According to the overall clinic blood pressure evaluations (sitting SBP and DBP and the 24h-Ambulatory Blood Pressure Monitoring):
    - To identify the oral doses of Rostafuroxin which lead to statistically significant differences, in comparison with Losartan, in either the total Caucasian (CEU) and Chinese (CH) patients or in the two separate sub-populations of patients carrying at least one gene included in the Genetic Profile 2 or a combination of the same genes either with the SNPs indicated in the Genetic Profile 2 or with other SNPs harboured on the genes of the Profile 2.

- Referring to the safety and tolerability:
  - to determine the safety profile of the three doses of Rostafuroxin.

3. Study Design

3.1 General design and plan

This is a Phase II multicenter, double-blind, double-dummy, four-arms, parallel group, active comparator controlled Study. Three oral doses of Rostafuroxin (6 - 50 - 500 micrograms) will be studied versus Losartan 50 mg. The Treatment Period will last 9 weeks.
The Study will last about 15 weeks and will be structured as follows:

A Screening Visit, a Run-In Period of 15 - 42 days (and a Treatment Period of 9 weeks (additional 7 days for Visits 4, 5 and 6 are allowed in case of late Visit).

Two hundred and eighty outpatients, approximately 120 CH Taiwanese (40 patients for 50, 500, micrograms of Rostafuroxin and Losartan arms) and 160 CEU Italian (40 patients for 6, 50, 500, micrograms of Rostafuroxin and Losartan arms,) who are just diagnosed with arterial hypertension and bearing a specific genetic profile (Genetic Profile 1), not assuming diuretics, ß-blocker agents, Caantagonist, ACE inhibitors and AT1-receptor blockers for other reason and finally not assuming statines, will be randomized in this Study and followed according to the standards of Good Clinical Practice. In order to be randomized, patients will have to meet all of the inclusion and none of the exclusion criteria, as specified in section 6.3.1. Diagnosis of arterial hypertension and genetic profile will be confirmed at the end of the Run-In Period (Visit 3).
Eligible patients will be randomized into one of the four Treatment arms, according to a randomization list stratified with respect of Study countries (Taiwan and Italy) and within each of them with respect of genetic subgroup (i.e. patients bearing and not bearing a mutation included in the Genetic Profile 2).
The Study will involve about 32 centres.

3.2 Visit Schedule and Visit Windows

Assessments and study visits will be performed as listed in the table below:

5.3 Study Diagram

	Screening Visit	Run-In Period		Treatment Period			
	Visit 1 (Screening)	Visit 2 (between Day 14 and 42)	Visit 3 V2+1 The day after Visit 2	Visit 4 (Day 14) Time window allowed: +7 days	Visit 5 (Day 35) Time window allowed: +7 days	Visit 6 (Day 63) Time window allowed: +7 days	Visit 7 (Day 64) The day after Visit 6
Patient's Informed Consent	X						
Past/Present Medical History	X						
Prior Medications	X						
Concomitant Medications	X	X	X	X	X	X	X
Physical Examination Including Body Weight and Height Measurement (only at Visit 1)	X	X		X	X	X	
Office Blood Pressure (OBP)	X	X	X	X	X	X	X
Heart Rate Measurement (HR)	X	X	X	X	X	X	X
ECG at Rest	X				X		X
Blood Sampling for Chemistry, Haematology and Pregnancy Test	X					X	
Urinalysis	X					X	
Blood Sampling for Genotype Determination	X						
Check of Inclusion/Exclusion Criteria	X		X				
Check of Concomitant Diseases Recovery		X	X	X	X	X	X
24 Hours Ambulatory Blood Pressure (with patient diary)		X........	X			X........	X
24 Hours Urine Collection [1]		........X[1]					
Blood Sample for Endogenous Ouabain (EO)			X			X	
Randomization			X				
Delivery to the Patient of the Patient's Diary			X				
Delivery to the Patient of a Letter for General Practitioner			X				
Study Drug Dispensing			X		X		
AEs Recording (if any)				X	X	X	X
Study Drug Accountability					X		X
Delivering by the Patient of the Filled-In Patient's Diary							X
Prescription of the Standard Antihypertensive Treatment							X

[1] - 24 Hours Urine Collection will start the 24 hours preceding the Visit 2.

To see the paragraph no. 5.2 "Study Plan" of Study Protocol and the DMP for details about of scheduled visits, time windows and the handling of data that fall outside the windows.

3.3 Sample size justification

Sample size has been computed by assuming a standard deviation of 10 mmHg either at the overall sample level (Genetic Profile 1) and in the pre-defined subgroup bearing the Genetic Profile 2. This assumption is based on an evaluation coming from the previous Study on PST 2238.
The other assumption is that the Genetic Profile 2 will comprise at least 50% of the total randomized patients.
Using these assumptions, a total sample size of 240 patients, i.e. 80 patients in the following groups (Rostafuroxin 50, 500 micrograms and Losartan 50 mg), is suitable for discriminating a SBP mean difference of 4.5 mmHg between the group of the two highest doses of Rostafuroxin and Losartan, with a standard deviation of 10mmHg, with a power of at least 80% and an alpha level of 0.05, two tailed test, in the PP population and in the ITT population assuming a 10% drop-out rate.
A total sample size of 120 patients (50% of the total sample), i.e. 40 patients in each group, is suitable for discriminating a SBP mean difference of 6.5 mmHg between the group of the two highest doses of Rostafuroxin and Losartan, with a standard deviation of 10 mmHg a power of

80%, an alpha level of 0.05, two tailed test, in the PP and ITT Genetic Profile 2 populations and assuming a 10% drop-out rate.
An interim blinded sample size re-assessment is planned and will be performed when approximately 70% of the patients (i.e. 170) will be randomized and will reach the final evaluation. All details of this interim analysis will be given in the paragraph 4.6 .

Interim blinded sample size re-assessment results
A sample size re-assessment was performed to confirm the sample size requirement for this trial.
For this task the interim analysis was performed on 221 randomized patients having the final evaluation (i.e. if the patient answered “Yes” to the question “Did the patient complete the study treatment period?” reported in the “End study” CRF form).
The endpoint considered for the standard deviation estimation was the change in the office measurement of sitting blood pressure from baseline (Visit 3) to Visit 6. Missing data was not replaced.
The sample size re-assessment showed a higher variability compared to the assumptions of the Study Protocol. In addition to this, as discussed in detail in the attached rationale, the very fast development of personalized medicine requires the most appropriate translation of the CRT data to the individualized day to day clinical practice. This has prompted the Sponsor to add as a co-primary end point an individual assessment of efficacy which identifies the responders and non-responders in each treatment arm.
Responders are defined as “Patients having the mean SBP (mean of last three measurements) <= 135 mmHg or having a reduction in mean SBP (mean of the three last measurements) > or = 10% with respect to the baseline measurement” at visit 6 (after two months of therapy).

Considering the sample size foreseen in the Study Protocol and the percentages of responders seen in previous trials, the table below shows the effect of these assumptions on the power:

Genetic Profile	Total No. of evaluable subjects	Total number of randomised subjects	Alpha Level	Reference proportion	Proportions difference scenarios	Power
1	216	240 (80 per group)	0.025	35%	20%	0.708
					30%	0.978
					40%	>.999
2	105	120 (40 per group)	0.025	30%	20%	0.380
					30%	0.758
					40%	0.963

Considering the actual number of patients who completed the study in Italy, at least 102 patients should be reached in Taiwan to guarantee the levels of power included in the table above.

3.4 Randomization and blinding

A computerized procedure generating pseudo-random numbers will be used. Assignment to treatment groups will be determined by a computer-generated random sequence using an interactive web-based response system (IWRS).
The randomization will be stratified by Study Country (i.e. Taiwan and Italy) in order to balance the different treatments (four arms in Italy and three arms in Taiwan) within different geographical areas and, within each Country, by genetic subgroup (one stratum will be constituted by the patients bearing a mutation included in the Genetic Profile 2, the other stratum will comprise all remaining patients). Blocks will be used for assuring the balance of the treatment allocations.
The randomization list will be generated by the same Contract Research Organization in charge to implement the centralized web system for Randomization of the patient and Treatment Number allocation (OPIS s.r.l.).

To preserve study blinding, access to the randomization table and treatment assignments will be restricted to a minimum number of people: to the Responsible of Pharmacovigliance for each Country, and to the Manufacturers Responsible for drug packaging prior to database lock.
If a subject is unblinded, he must be discontinued from the study.
The Treatment assignment will be carried-out under blind conditions throughout a secured and dedicated web site.
When a patient has qualified as eligible (i.e. he/she fulfils all the inclusion criteria and does not carry any exclusion criteria of the Study Protocol), the Investigator or his/her designee will request the patient's randomization code. Each Study centre must request these randomization codes for all their patients in the order to qualify them for the Study.
The procedure for generating the list, maintaining the blinding, managing the random allocation and unblinding the individual randomisation codes, if needed, will be detailed in a specific document.
The Investigators will be allowed to break individual codes exclusively when a Serious Adverse Event occurs and knowledge of the treatment by the Investigator is deemed useful as to the patient's safety. Individual code breaking must be clearly indicated in the patient related SAE form.
Breaking of individual randomization codes is also allowed:

- to the Drug Safety Unit of CVie Therapeutics Company Limited, or its Pharmacovigilance Responsible delegate for Italy during the course of the Study, in order to comply with the regulatory requirements;
- to the Data and Safety Monitoring Board (DSMB), in order to give advice to the Sponsor in case of safety concerns. In this case, the DSMB will ask to the Drug Safety Unit of CVie Therapeutics Company Limited, to open the code and give them notice of the treatment.

3.5 Efficacy endpoints

3.5.1 Primary endpoints

- Change from baseline to Visit 6 in office sitting SBP.
- Proportions of responders at Visit 6, defined as “Patients having the mean office SBP (mean of last three measurements) <= 135 mmHg or having a reduction in mean office SBP (mean of the three last measurements) > or = 10% with respect to the baseline measurement” at Visit 6 (after two months of therapy).

3.5.2 Secondary efficacy endpoints

The secondary efficacy endpoints are the following:

- Change from baseline to Visit 6 in office sitting DBP.
- Office sitting DBP and office sitting SBP values and changes from baseline at each study visit.
- 24 hours SBP and DBP measurements. 24 hours, Day-time and night-time weighted mean value of both SBP and DBP.
- Office sitting SBP and DBP corrected as per seasonal variation of temperature (confounding factor).

3.5.3 Exploratory efficacy endpoints

Additional exploratory analyses may be performed to identify the oral doses of Rostafuroxin which lead to statistically significant differences, in comparison with Losartan, in either the total CEU and CH patients or in the two separate sub-populations of patients carrying at least one gene included in the Genetic Profile 2 or a combination of the same genes either with the SNPs indicated in the Genetic Profile 2 or with other SNPs harboured on the genes of the Profile 2.

3.6 Safety endpoints

The safety endpoints are the following:

- Physical examinations.
- ECG.
- Vital signs.
- Laboratory tests.
- Adverse events.
- Concomitant medications.

4. Statistical Analysis

4.1 General

Descriptive statistics will be provided for all variables in the summary tables by treatment group according to the type of variable summarised.
Quantitative variables will be summarised by using n, arithmetic mean, SD, median and range (minimum and maximum).

Categorical variables will be summarised by using frequency distributions and percentages.

Hypothesis testing related to the primary efficacy endpoints will be performed as described in "Primary efficacy analysis - Multiplicity adjustment procedure" section.
Hypothesis testing related to the secondary efficacy endpoints will be carried out at the alpha = 0.05 level (two-sided) when comparing treatments. For all inferential analyses, p-value will be rounded to three decimal places. Statistical significance will be declared if the rounded p-value will be less than or equal to 0.05.
All data collected in the CRF will be presented in the listings.

Some statistical analyses are considered as optional. The decision on performing these or not will be taken after checking the main efficacy statistical analysis results.

4.2 Analysis sets

4.2.1 Full analysis set (FAS)

The FAS includes all randomised patients who received at least one dose of the study drug and have at least one post-baseline efficacy assessment.
All efficacy endpoints will be analysed on the FAS.

4.2.2 Per Protocol set (PPS)

The PPS includes all randomised patients of the FAS who have at least one post-baseline efficacy assessment at visit 5 or visit 6 and who fulfil the eligibility criteria of the
Protocol, perform the Visit at the end of the Treatment Period, complete the treatment with a compliance of at least 80%, don't take prohibited drugs during the course of the Study and don't discontinue the Study Drug treatment during the day before or the day of each scheduled Visit.
During the Data Review Meeting will be evaluated if a patient interrupting the Study because of uncontrolled hypertension (i.e. office SBP $\geq$ 179 mmHg or DBP $\geq$ 110 mmHg) will or not be included in the statistical analysis with his/her last blood pressure evaluation (see Data Review Report, version 1.0, 20 April 2018).
If, within each treatment group, in the subpopulations of Italian or Chinese patients respectively, a patient belongs to genetic profile 2 because a gene pair alone (i.e. gene pair not included as part of gene pairs combinations) which is not represented by any other within the same group, then ALL the patients carrying such gene pair will be excluded from the PPS.

The primary efficacy endpoint will be analysed on the PPS.
The analyses of the secondary efficacy endpoints on the PPS are considered as optional.

4.2.3 Safety set

The Safety set includes all randomised patients who receive at least one dose of the study drug.
Analysis of safety variables and demographic and baseline characteristics will be performed on the safety set.

4.2.4 Screened set

The Screened set includes all patients with signed and dated written informed consent and undergoing to study evaluations/procedures of Visit 1.

4.2.5 Randomized set

The Randomised set consists of all patients randomized in the study.
Disposition of patients, primary reason for discontinuation and all listing will be based on Randomized set.

4.2.6 Incorrect treatment allocations

In case an error occurs in treatment allocation, the following rule will be followed: if a patient was randomized but received the incorrect treatment, he/she will be reported under his/her randomized treatment group for all analyses performed on the randomized and full analysis sets, but he/she will be reported under the treatment actually received for all analyses performed on the safety set. The patient will be excluded from the PPS.

4.3 Sub-group analyses

The following subgroups will be considered.

Genetic Profile

- Genetic Profile 2 sub-group: subset of Genetic Profile 1 patients (i.e. overall sample) with Genetic Profile 2.
- Genetic Profile 3 sub-group: subset of Genetic Profile 1 patients (i.e. overall sample) with Genetic Profile 3.

Ethnic origin

- Caucasian (CEU)
- Chinese (CH)

4.4 Covariates

All analysis of covariance (ANCOVA) models will include the baseline value of the dependent variable as a covariate.
The baseline used in the statistical analysis for each variable is reported in the table below:

Endpoint	Baseline
Office measurement of systolic and diastolic blood pressure (SBP/DBP)	Visit 3(if available)*

24-Hours Systolic and Diastolic Blood Pressure Monitoring: 24 hours, day-time and night-time weighted mean values	Visit 2-Visit 3
Laboratory parameters (Haematology, Blood Chemistry and Urinalysis)	Visit 3
Vital signs	Visit 3(if available)*
ECG	Visit 3

*Baseline measurement is Visit 3 if available, otherwise if the baseline measurement is missing but there is a prior measurement recorded at Visit 2, this value will be carried forward and used as the baseline measurement.

4.5 Pooling of sites

Pooling of sites will be evaluated during the Data Review meeting.

4.6 Interim analyses

An adaptive design will be used to determine the sample size requirement for this trial. For this task an interim analysis will be performed after approximately 70% of patients (i.e. 170) will be randomized and will have the final evaluation.
A patient will be considered having the final evaluation if the patient will answer "Yes" to the question "Did the patient complete the study treatment period?" reported in the "End study" CRF form.

The interim analysis will be performed as follows:

1. Blinded standard deviation estimation (within-group SD)
   The endpoint considered for the standard deviation estimation is the change in the office measurement of sitting blood pressure from baseline to visit 6.. All interim analyses will be performed on the randomized set.
   The A. Lawrence Gould method will be applied for the within-group SD estimation. This method does not require unblinding. Therefore, the following formula will be used to calculate the within-group SD:

$$\hat{\sigma} = \sqrt{\frac{n-1}{n-2}\left(\hat{s}^2 - \frac{1}{4}\hat{\delta}^2\right)}$$

   Where:
   - $\hat{\sigma}$ is the within-group SD to be used in the sample size re-estimation.
   - n is the interim analysis sample size.
   - $\hat{s}^2$ is the variance of the change in the office measurement of sitting blood pressure from baseline to visit 6, calculated by considering the interim analysis sample.

- $\hat{\delta}$ is the mean difference between the group of the two highest doses of Rostafuroxin and Losartan under the alternative hypothesis. It will be assumed to be equal to 4.5.

2. Drop-out rate estimation

The statistician will calculate the drop-out rate of the interim analysis sample.

3. Sample size re-estimation

Once the blinded standard deviation and the actual drop-out are calculated, the statistician will determine the final sample size using the same test (a two-tailed T-test) and the same assumptions reported in the paragraph 3.3:
- α=0.05
- β=0.20
- A mean overall difference between treatment of 4.5 (and of 6.5 for the Genetic Profile 2)
- The SD and the drop-out rate estimated as indicated in the previous section 1 and 2.

4. Patients with Genetic Profile 2.

The statistician will determine the proportion of randomized patients with the Genetic Profile 2.

5. Communication of the interim analysis results

The statistician will inform the Sponsor about the final sample size, the actual proportion of Genetic Profile 2, and the number and the percentage of patients discontinued after randomization with their respective reasons. The Sponsor will take into account this information to decide if further patients are to be recruited to reach the requirement sample size and if an adjustment of the Genetic Profile type is needed to have approximately half of patients with Genetic Profile 2. No other information will be conveyed.

4.7 Handling of missing and incomplete data

The number of patients with missing data will be presented under the “Missing” category, if present. Missing values will be included in the denominator count when computing percentages.
When continuous data are being summarized, only the non-missing values will be evaluated for computing summary statistics.
Missing or incomplete data will be treated as described in sections 5, 6, 7 and 8. Other critical missing data will be discussed prior to treatment unblinding, if any. Decisions will be taken during the data review and will be fully documented in the Data Review Report.

The LOCF method of imputation will be applied for missing values on the FAS in the statistical analyses of the following efficacy variables: office sitting SBP, office sitting DBP, Site temperature. The proportions of responders will be based on imputed office sitting SBP values with LOCF.
For patients taking prohibited drugs (see sections of the study protocol 7.5), the assessments carried-out after prohibited drugs intake (i.e., date of assessment≥ start date of prohibited drugs) will be disregarded. In their place, the last available assessments before the administration of prohibited drugs will be taken into account for the statistical analysis and treated with the LOCF method.

Only data of treatment visits (Visit 4, Visit 5) will be considered in the LOCF (i.e., baseline visit is excluded).
This method will not be applied toward descriptive statistics. The LOCF method will not be applied toward the other analysis sets.
LOCF values will be reported in a dedicated column in patient listings.

4.8 Changes in the planned analysis

The following changes have been implemented in the SAP:

- The ANCOVA models will be used instead of ANOVA models in the efficacy analysis.
- Normal-abnormal shift tables will not be presented for physical examination results but only data listings will be presented.
- As requested by the Sponsor, the PPS will include all randomised patients who received at least one dose of the study drug and have at least one post-baseline efficacy assessment at visit 5 or visit 6, instead of at least one post-baseline efficacy assessment at any timepoint. Also, the following a priori restriction to be applied to PPS was agreed during the blind review of the data (see also Data Review Report, version 1.0, 20 April 2018):
  If, within each treatment group, in the subpopulations of Italian or Chinese patients respectively, a patient belongs to genetic profile 2 because a gene pair alone (i.e. gene pair not included as part of gene pairs combinations) which is not represented by any other within the same group, then ALL the patients carrying such gene pair will be excluded from the PPS.

4.9 Data Review Meeting

A Data Review Meeting will be held after Database lock and before the Database freeze and the unblinding of the data. Any other details will be provided in the Data Review Report.

4.10 Software

All statistical analyses and data processing will be performed using Statistical Analysis Systems (SAS®) Software (release 9.4) on a Windows 7 operating system.

5. Evaluation of Demographic and Baseline Characteristics

Definitions and data conventions

<u>Date of first randomised study drug intake</u>
The date of first randomized study drug intake is assumed to be the "date of first drug intake" reported in the "Randomisation" CRF form.

Date of last randomised study drug intake
The date of last randomized study drug intake is assumed to be the "date of last treatment administration" reported in the "End study" CRF form.

Completer/discontinued Patient
A Patient will be considered as completer if the answer to the question "did the patient complete the study treatment period?" will be equal to "Yes".

A Patient will be considered as discontinued if the answer to the question "did the patient complete the study treatment period?" will be equal to "No".

5.1 Subject enrolment and disposition

Disposition of patients will be presented by treatment and overall.
The number of patients included in each of the screened, randomised, safety, FAS and PP sets will be summarized for each treatment and overall.

5.2 Protocol violations

Definitions and data conventions
All the protocol violations will be discussed case by case before unblinding of the treatment code with the clinical team during the blind review of the data and described in the Data Review Report.

Statistical analysis
Major and minor protocol violations will be presented in the listings only.

5.3 Study discontinuations

The number and the proportion of randomised patients who discontinue from the study prematurely will be presented with a breakdown of the reasons for discontinuation by treatment and overall for the randomized set.

5.4 Demographics and baseline characteristics

Definitions and data conventions
Age
If the date of birth is not missing, age will be calculated using the following rules:

- If month of screening visit date is greater than month of date of birth or (month of screening visit date is equal to month of date of birth and day of screening visit date is greater than or equal to day of date of birth), then:

Age (years) = year (date of screening visit) – year (date of birth)

- Otherwise:

Age (years) = year (date of screening visit) – year (date of birth) - 1

In order to determine the age of patient, the following rules will be applied for the partial date of birth:
- if only the day is missing, the 15th of the month will be assumed;
- if the day and the month are missing, 30th June will be assumed.

Ethnic origin
The Oriental ethnic origin will be presented as Chinese.

Statistical analysis

The baseline demographic characteristics will be summarized by treatment group and overall by means of descriptive statistics.

The following characteristics will be provided for the Safety set:
- Age (years)
- Sex (male/female)
- Ethnic origin (Chinese, Black, Caucasian, Hispanic, Other)
- Weight (kg) at screening
- Height (cm) at screening
- BMI (kg/m^2) at screening

5.5 Medical and surgical history

Definitions and data conventions
Past disease
A disease is considered as past disease if it is not ongoing at screening visit ("ongoing" box is not ticked).

Concomitant disease
A disease is considered as concomitant disease if it is ongoing at screening visit ("ongoing" box is ticked).

Statistical analysis
Past disease and concomitant diseases will be coded using Medical Dictionary for Regulatory Activities (MedDRA) (version 15.1) and frequency distributions and percentages will be summarized by treatment group for the safety set by System Organ Class (SOC) and Preferred Term (PT).
Counts will be given for both SOC and PT by subject. Subjects experiencing more than one past disease (or concomitant disease) event will be counted only once within each SOC, PT.

5.6 Prior and concomitant medications

Definitions and data conventions
The following categories of medications will be identified:
- previous medication (start date < date of first randomised study drug intake);
- concomitant medication (start date ≥ date of first randomised study drug intake);

In case of missing or incomplete dates/times not directly allowing allocation to any of the three categories of medications, a worst-case allocation will be done according to the available parts of the start and the end dates/times. The medication will be allocated to the first category allowed by the available data, according to the following order:
- concomitant medication;
- previous medication.

Statistical analysis
Previous and Concomitant medications
Medications will be coded using WHO Drug Dictionary (WHO-DD), 2012Q1, using the following Anatomical Therapeutic Chemical Classification (ATC) codes;
- Anatomical Main Group (ATC 1st level code);
- Chemical subgroup (ATC 4th level code);
- Preferred name.

Previous and concomitant medications will be summarized separately for safety set by anatomical main group, chemical subgroup and preferred name by treatment group.
Subjects experiencing more than one previous (or concomitant) medication within the same anatomical main group, chemical subgroup and preferred name will be counted only once.

No formal comparison between treatments on baseline characteristics will be done.

6. Evaluation of Treatment Compliance and Exposure

Definitions and data conventions
Date of first randomized study drug intake
The date of first randomized study drug intake is assumed to be the "date of first drug intake" reported in the "Randomisation" CRF form.

Date of last randomized study drug intake
The date of first randomized study drug intake is assumed to be the "date of last treatment administration" reported in the "End Study" CRF form.

6.1 Compliance to study drug and treatment

Definitions and data conventions
Compliance
Compliance to study drug will be evaluated on the basis of the information recorded on the CRF.
On a per patient basis, the evaluation of the compliance during the treatment period will be done using the following formula:

$$\text{Compliance (\%)} = \frac{\text{Total number of study drugs taken during the treatment period}}{\text{Total number of scheduled study drugs during the treatment period}} \times 100$$

The formula above will be used to calculate overall compliance, Rostafuroxin compliance and Losartan compliance, by considering the following indications:

Overall	Total number of	
	Study drugs taken	Scheduled study drugs
	Sum of the differences between "Number of capsules/tablets given to the patient" and "Number of capsules/tablets returned by the patient" by considering all the records reported in the "List of supplies administered to the patient" CRF form.	2 capsules/tablets x (Date of last last randomized study drug intake - Date of first randomized study medication intake +1).
Rostafuroxin	Total number of	
	Study drugs taken	Scheduled study drugs
	Sum of the differences between "Number of capsules given to the patient" and "Number of capsules returned by the patient" by considering only the Rostafuroxin / Placebo records reported in the "List of supplies administered to the patient" CRF form.	1 capsule x (Date of last last randomized study drug intake - Date of first randomized study drug intake +1).
Losartan	Total number of	
	Study drugs taken	Scheduled study drugs
	Sum of the differences between "Number of tablets given to the patient" and "Number of tablets returned by the patient" by considering only the Losartan / Placebo records reported in the "List of supplies administered to the patient" CRF form.	1 tablet x (Date of last last randomized study drug intake - Date of first randomized study drug intake +1).

Also, compliance calculation will be based on study treatment periods when effectively patients received the study medication only; such information, included in the study medication dispensing section (i.e. patients for which "Yes" has been reported in the study medication dispensing CRF section for question "Has study medication (for 1st Treatment Period Week 1-5) been dispensed to

the patient" for the related treatment period) will be merged with Drug Accountability and used for final analysis production.
In particular, compliance for patients with unknown (UK) for " No. of caps/tabs returned "variable will not be calculated.

Overall compliance, Rostafuroxin compliance and Losartan compliance will be classified according to the following criteria:

- ≤ 50% (if Compliance ≤ 50%)
- 50-80% (if 50 < Compliance ≤ 80%)
- 80-100% (if 80 < Compliance ≤ 100%)
- 100-125% (if 100 < Compliance ≤ 125%)
- 125% (if Compliance > 125%)
- ≥ 80% (if Compliance ≥ 80%)

Statistical analysis
Overall compliance, Rostafuroxin compliance and Losartan compliance to study treatment during the treatment period will be summarised using descriptive statistics by treatment group.
Overall compliance, Rostafuroxin compliance and Losartan compliance will be also categorised (≤ 50%, 50-80%, 80-100%, 100-125%,>125%, ≥ 80%) and the number and the percentage of patients in each category of compliance will be presented.

Compliance will be analysed in the safety set.

6.2 Exposure to study drug

Definitions and data conventions
The extent of exposure (days) will be calculated using the following formula:

Extent of exposure (days) = Date of last randomised study drug intake - Date of first randomised study drug intake +1.

Statistical analysis
Extent of exposure will be summarized by treatment group using descriptive statistics on the safety set.

7. Evaluation of Efficacy

7.1 Analysis of primary endpoint

Definitions and data conventions
<u>Change from baseline to Visit 6 in office sitting SBP</u>
For this endpoint, the average of the last three readings reported in the CRF will be considered in the statistical analysis.

Proportions of responders at Visit 6

A responder patient is defined as “Patient having the mean office SBP (mean of last three measurements) <= 135 mmHg or having a reduction in mean office SBP (mean of the three last measurements) > or = 10% with respect to the baseline measurement” at Visit 6 (after two months of therapy).

Gene pair information

Additional genetic information including specific gene pairs for patients belonging to genetic profile 2, which was not part of the planned study data collection, but available to the Sponsor from the Division of Nephrology and Hypertension (University of Milan - San Raffaele Hospital, Milan – ITALY), will be incorporated into the final study database for statistical analysis purpose (see also modified PPS definition, referencing to such additional gene pair information).

Site temperature (°C)

The data of the temperature will be provided by a dedicated provider.

These data will be provided for the main cities of Italy and Taiwan. The matching between the temperature external data and CRF data will be performed for all the visits from visit 2 to visit 6 by joining the study site with the nearest city with temperature data available (and the date and time of temperature with the visit date and timing of Office Blood Pressure (OBP) measurement start. If the visit date and/or the timing of OBP measurement start are missing, the temperature will not be associated to OBP and it will be set as missing.

Since the temperature data will be provided at each hour of the day, the temperature associated to the timing of OBP measurements start (hh:mm) will be calculated as follows

Temperature at time hh:mm (OBP start) = [temperature at hh:00 + temperature at hh+1:00]/2

Primary efficacy analysis - Genetic Profile 2

Change from baseline to Visit 6 in office sitting SBP

All analyses described here below will be performed on the Genetic Profile 2 sub-group.

SBP values will be summarized by treatment group and visit by means of descriptive statistics. At each post-baseline visit, mean and SD of the change from baseline will also be calculated with the relative 95% CI.

A comparison between treatment groups will be performed with an ANCOVA model with change from baseline to visit 6 as dependent variable, treatment, country and treatment x country as fixed effects, and baseline as covariate. If a significant effect of country (i.e. Ethnic origin) and/or treatment x country is observed, a sub-group analysis will be performed by country, as explained in “Secondary efficacy analyses” section.

The number of patients and the number of patients considered in the model will be provided by treatment. P-values of the fixed effects and covariates will also be presented.

The adjusted means for treatments, the relative 95% CIs and p-values will be presented.

The difference between the adjusted means for the two highest doses of Rostafuroxin and Losartan will be calculated with the relative 95% CI and p-value by using the appropriate linear contrast.

Additional differences between the adjusted means will be calculated (with the relative 95% CI and p-value) for the three doses of Rostafuroxin one another and each of the three doses versus Losartan.

<u>Proportions of responders at Visit 6</u>
The proportion of responders in each treatment group will be presented.
A comparison between the two highest doses of Rostafuroxin versus Losartan in terms of proportions of responders will be performed using the Mantel-Haenszel test stratified by country.

Additional comparisons will be will be performed using the Mantel-Haenszel test stratified by country for the three doses of Rostafuroxin one another and each of the three Rostafuroxin doses versus Losartan.

Primary efficacy analysis - Genetic Profile 1 (Overall sample)
The same analyses described previously will be performed on the Genetic Profile 1 (Overall sample).

Primary efficacy analysis - Multiplicity adjustment procedure
Hypotheses concerning the two Genetic profiles will be tested in a hierarchical order.

The hypotheses (H_1 and H_2) related to the genetic profile 2 will be tested first. The alpha level will be adjusted to take into account the two co-primary endpoints, linked by the “OR” condition. Therefore, the alpha level will be set to 0.025 (α/2).

The same hypotheses (H_3 and H_4) will be tested in the genetic profile 1 only if at least one of H_1 or H_2 are rejected.

The following parallel gatekeeping procedure will be applied for alpha propagation:

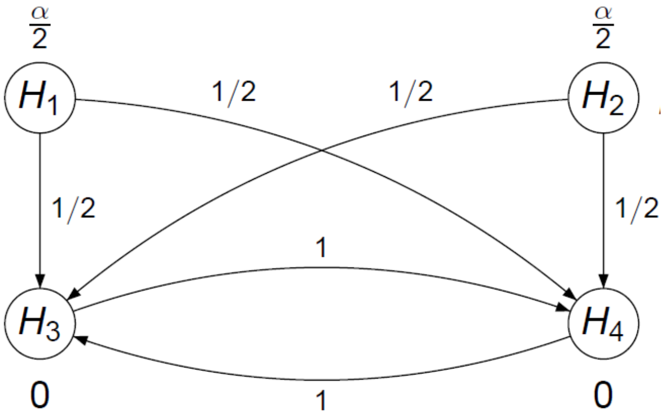

Where

- $\alpha = 0.05$
- H_1 = primary analysis performed on the change in the office measurement of sitting blood pressure from baseline (Visit 3) to Visit 6, tested in the genetic profile 2

- H_3 = primary analysis performed on the change in the office measurement of sitting blood pressure from baseline (Visit 3) to Visit 6, tested in the genetic profile 1
- H_2 = primary analysis performed on the % of responders, tested in the genetic profile 2
- H_4 = primary analysis performed on the % of responders, tested in the genetic profile 1

The two hypotheses H_1 and H_2 from the primary family of tests F_1 are initially assigned the local level $\alpha/2$ each, whereas H_3 and H_4 from the secondary family F_2 are assigned the local level 0. If H_1 and/or H_2 are rejected, the local level $\alpha/2$ is split into half and passed on to H_3 and H_4 as indicated by the directed edges with weights ½. If $H_3(H_4)$ is rejected in the subsequence at its local significance level (either $\alpha/2$ or $\alpha/4$), this level is passed to $H_4(H_3)$ as indicated by the directed edges with weights 1.

Secondary efficacy analyses
Genetic Profile 3
The same analysis described for the “Primary efficacy analysis - Genetic Profile 2” will be performed by considering the Genetic Profile 3 sub-group.

ANCOVA models – sensitivity analyses
All ANCOVA models described in the primary analyses will be replicated by adding the level of Sodium at visit 3 as covariate in the model.

All ANCOVA models described in the primary analyses will be replicated by adding the site actual temperature change from baseline to visit 6 as covariate in the model.

Ethnic origin
The same analysis described for the “Primary efficacy analysis - Genetic Profile 2” will be performed by considering Caucasian and Chinese sub-groups, separately.

SAS® code
The following SAS® code will be used to estimate the ANCOVA model for the primary efficacy analysis:

```
proc mixed data = dataset;
class tmt country;
model change = tmt country tmt*country baseline;
lsmeans tmt / cl;
estimate ‘6 mcg Rostafuroxin - 50 mcg Rostafuroxin' tmt 1 -1 0 0 / cl;
estimate ‘6 mcg Rostafuroxin - 500 mcg Rostafuroxin' tmt 1 0 -1 0 / cl;
estimate 6 mcg Rostafuroxin - Losartan ' tmt 1 0 0 -1 / cl;
estimate '50 mcg Rostafuroxin - 500 mcg Rostafuroxin' tmt 0 1 -1 0 / cl;
estimate 50 mcg Rostafuroxin - Losartan ' tmt  0 1 0 -1 / cl;
estimate '500 mcg Rostafuroxin – Losartan' tmt 0 0 1 -1 / cl;
estimate ‘50/500 mcg Rostafuroxin – Losartan ' tmt 0 0.5 0.5 -1 / cl; /*primary comparison*/
run;
```

where change represents the change from baseline to visit 6, tmt the treatment group, baseline the baseline value of the variable.
The variable tmt will be ordered in SAS in the following order:
1 = "6 mcg Rostafuroxin";
2 = "50 mcg Rostafuroxin";
3 = "500 mcg Rostafuroxin";
4 = "Losartan".

The following SAS® code will be used to estimate the Mantel-Haenszel test for primary comparison:

```
proc freq data = dataset;
tables country*tmt*responder / cmh;
where tmt in (1 or 2)
run;
```

The Mantel-Haenszel test corresponds to the Nonzero Correlation.
The variable tmt will be ordered in SAS in the following order:
1 = "6 mcg Rostafuroxin";
2 = "50 mcg Rostafuroxin" or "500 mcg Rostafuroxin";
3 = "Losartan".

The following SAS® code will be used to estimate the Mantel-Haenszel test for the other groups comparisons:

```
proc freq data = dataset;
tables country*tmt*responder / cmh;
where tmt in (1 or 4) ; /*example: 6 mcg Rostafuroxin vs Losartan*/
run;
```

The Mantel-Haenszel test corresponds to the Nonzero Correlation.
The variable tmt will be ordered in SAS in the following order:
1 = "6 mcg Rostafuroxin";
2 = "50 mcg Rostafuroxin";
3 = "500 mcg Rostafuroxin";
4 = "Losartan".

7.2 Analysis of secondary efficacy endpoints

Definitions and data conventions

Office measurement of sitting diastolic blood pressure (DBP).
For this endpoint, the average of the last three readings reported in the CRF will be considered in the statistical analysis.

24-Hours Systolic and Diastolic Blood Pressure Monitoring: 24 hours, day-time and night-time weighted mean values

The 24 hours weighted mean value will be calculated as the AUC of SBP or DBP values through two-day visit period by means of the trapezoidal rule, divided by the width:

$$AUC_{twodays} / width = \frac{\left[\sum_{i=1}^{n} \frac{(t_i - t_{i-1})(VAR_i + VAR_{i-1})}{2} \right]}{t_n - t_0}$$

where:

- t represents the recorded time defined as the number of minutes from midnight of the first of two days considered.
- t_0 represents the recorded time of the first measurement and t_n indicates the recorded time of the last measurement.
- VAR_i is the SBP or DBP value at the time t_i

The day-time and night-time weighted mean value will be calculated with the same formula above considering the following rules:

- day-time: only the measurements recorded from 10 AM to 8 PM will be considered in the calculation
- night-time: only the measurements recorded from 00 AM (midnight) to 6 AM will be considered in the calculation

In the calculation of AUC/width, missing SBP or DBP values will not be replaced. The 24 hours, day-time and night-time AUC/width will be based on available recorded measurements in the correspondent period.

In addition, the following rules will be applied:

- The first measurement recorded will be the first available measurement at visit 2 and the first available measurement at visit 6.
- The last measurement recorded considered have to be less than 25 hours after the first measurement;
- Only measurements from the first measurement recorded to the last measurement recorded will be considered.
- the 24 hours and day-time AUC/width will not be calculated and will be considered as missing if less than 10 readings are recorded between 10 AM and 8 PM hours;
- the 24 hours and night-time AUC/width will not be calculated and will be considered as missing if less than 5 reading are recorded between 00 AM and 6 AM in the morning;
- the 24 hours AUC/width will not be calculated and will be considered as missing if the recording (from the first measurement recorded to the last measurement recorded) covers less than 20 hours;
- the 24 hours, day-time or night-time AUC/width will not be calculated and will be considered as missing if no valid measurements are available for 3 or more consecutive hours within the period considered (24 hours, day-time or night-time, respectively).
- Any additional editing of extreme values will be evaluated during the Data Review Meeting (see Data Review Report, Version 1.0, 20 April 2018).

Statistical analysis
All the statistical analyses described in this section will be performed in the Genetic Profile 1 (Overall sample) and in the Genetic Profile 2 subgroup of patients.

<u>Office measurement of sitting diastolic blood pressure (DBP).</u>
The same analyses specified for the primary efficacy analysis will be performed for this secondary variable. The p-value of the difference between the adjusted means for the two highest doses of Rostafuroxin and Losartan will be presented in both Genetic profile 1 and Genetic profile 2 for exploratory purposes.

<u>24-Hours Systolic and Diastolic Blood Pressure Monitoring: 24 hours, day-time and night-time weighted mean values</u>
The weighted mean values of SBP and DBP (24 hours, day-time and night-time) will be summarized by treatment group and visit by means of descriptive statistics. At post-baseline visit, mean and SD of the change from baseline will also be calculated with the relative 95% CI.
A comparison between treatment groups will be performed with an ANCOVA model with change of each parameter from baseline to visit 6-visit 7 as dependent variable, treatment, country and treatment x country as fixed effects, and baseline (value at visit 2-visit3) as covariate.
The number of patients and the number of patients considered in the model will be provided by treatment. P-values of the fixed effects and covariates will also be presented.
The adjusted means for treatments, the relative 95% CIs and p-values will be presented.

The difference between the adjusted means for the two highest doses of Rostafuroxin and Losartan will be calculated with the relative 95% CI and p-value by using the appropriate linear contrast. Additional differences between the adjusted means will be calculated (with the relative 95% CI and p-value) for the three doses of Rostafuroxin one another and each of the three doses versus Losartan.

Exploratory analysis

Additional exploratory analyses may be performed to identify the oral doses of Rostafuroxin which lead to statistically significant differences, in comparison with Losartan, in either the total CEU and CH patients or in the two separate sub-populations of patients carrying at least one gene included in the Genetic Profile 2 or a combination of the same genes either with the SNPs indicated in the Genetic Profile 2 or with other SNPs harboured on the genes of the Profile 2.

An additional analysis could be performed considering the subgroup of patients with LSS or other genes. Data about LSS, as part of the additional genetic information available to the Sponsor from the Division of Nephrology and Hypertension (University of Milan - San Raffaele Hospital, Milan – ITALY), will be incorporated into the final study database for potential statistical analysis purpose (see also section 7.1 above and modified PPS definition, referencing to such additional genetic information).

8. Evaluation of Safety

8.1 Adverse events

Definitions and data conventions

Date of first randomised study drug intake

The date of first randomized study drug intake is assumed to be the "date of first drug intake" reported in the "Randomisation" CRF form.

Pre-treatment adverse events

An AE will be classified as pre-treatment AE if it starts before the date of first randomised study drug intake (AE onset date/time < date of first randomised study drug intake).

Treatment-emergent adverse event (TEAE)

An AE will be classified as a TEAE if it starts on or after the date of first randomised study drug intake (AE onset date ≥ date of first randomised study drug intake).

In case of missing or incomplete dates/times not allowing a direct allocation to any of the two categories of AEs, a worst-case allocation will be done according to the available parts of the onset and the end dates. The AE will be allocated to the first category allowed by the available data, according to the following order:

- TEAE
- Pre-treatment.

Serious adverse event (SAE)

A SAE is an AE judged as serious.

Adverse drug reaction (ADR)

An ADR is an AE with the relationship to study treatment judged as "Suspected".

Adverse event leading to discontinuation

An AE leading to discontinuation is an AE with action taken equal to "Study drug permanently discontinued due to this AE".

Adverse event leading to death

An AE leading to death is an AE with outcome equal to "Death".

Count of adverse events

Two AEs with the same Preferred Term (PT) and classified in the same category (pre-treatment AE or TEAE) will be considered as two different events when calculating the "number of events" in the tables.

Statistical analysis

Pre-treatment AEs and TEAEs will be presented separately. Pre-treatment AEs will be presented in the listings only.

The number of treatment-emergent AEs, SAEs, ADRs, AEs leading to discontinuation and AEs leading to death, and the number and the percentage of patients experiencing treatment-emergent AEs, SAEs, ADRs, AEs leading to discontinuation and AEs leading to death will be summarised by treatment group.

AEs will be coded using the MedDRA dictionary (version 15.1). The SOCs and PTs will be used for tabulation. The number of AEs and the number and the percentage of patients with at least one AE will be presented by SOC and PT for treatment-emergent AEs, SAEs, ADRs, AEs leading to discontinuation and AEs leading to death by treatment group.

8.2 Clinical laboratory evaluation

Shift tables presenting the number and the percentage of patients in each bivariate category (baseline visit versus visit 6) with regards to normal range (Low, Normal, High) will be presented by treatment for all laboratory parameters (Haematology, Blood Chemistry).
Urinalysis parameters will be only listed.

8.3 Vital signs

Definitions and data conventions
The results of re-test of patient office blood pressure (OBP) within 1 week will not be considered in the calculation of descriptive statistics. These values will be only listed.

Standing position blood pressure (Systolic and Diastolic)
The average of the two measurements reported in the CRF will be calculated for all the study visits and considered in the statistical analysis.

Statistical analysis
Sitting heart rate, standing position blood pressure (Systolic and Diastolic), standing heart rate and body weight with the relative changes from baseline will be summarised by treatment group at each visit by means of descriptive statistics.

8.4 ECGs

Shift table presenting the number and the percentage of patients in each bivariate category (baseline versus Visit 5 and baseline versus Visit 7) with regards to investigator’s interpretation (Normal, Abnormal) will be provided by treatment group.

8.5 Physical examination

Significant physical abnormality will be presented in the listings only.

9. Tables, Figures and Listings

9.1 Programs and tables quality control

The statistician-programmer of the tables, listings and figures will carefully review the programs and will verify that no error message is highlighted in the 'LOG' file.
Moreover, a second statistician-programmer will verify the internal consistency of each table and figure by checking the results using different SAS programs.

The following level of validation will be implemented:

Validation of statistical datasets: via validation of the final output (e.g. tables).

Validation of statistical output:
- tables: independent programming, with a comparison of the results against the initial output.
- listings: peer review of the program code used to create the output.

9.2 Programming conventions

All tables/figures/listings will be presented in landscape format.
The standard font size is 9 points Courier New for all tables. Listings will be presented with an 8 or 7 points Courier New.

Titles will be center-aligned; footnotes will be left-aligned.
Each table/figure/listing will have 2 titles:

- The 1st title will be the table/figure/listing number with the description of the table/figure/listing;
- The 2nd title will be a description of the study population presented in the table/figure/listing.

Some tables will have a third title (before 2nd title) with a description of the statistical method used in those tables.

Any footnote added to explain the table/listing/figure contents will be presented in the following format:
Note 1: Percentages are calculated on the number of patients (N).
Note 2: A serious adverse event is an ....
Note 3: ……….

The last two footnotes of each table/figure will be footers indicating:

- the reference listing of the data;
- the program name, the date and time of generation and the SAS® version used.

The last footnote of each listing will be a footer indicating the program name, the date and time of generation and the SAS® version used.

In the tables, listing and figures the treatment under comparison will be labelled as "6 mcg Rostafuroxin", "50 mcg Rostafuroxin","500 mcg Rostafuroxin" and "Losartan".

Unless otherwise stated, listings will be presented by randomised treatment, and sorted by the patient number.

Except for the listings 16.2.1-2.1 (based on screening failure patients) and 16.2.7-1 (based on all patients), all the listings will be based on the randomised set.

In all the listings on safety variables, a column with a flag (§) for treatment misallocation will identify the treatment misallocations.
The derived variables will be identified in the listings with a flag (*).

In general, dates will be presented on listings in the format ddmmmyyyy (date9.) and time in the format hh:mm (time5.). In case of partial dates or times, missing information will be replaced by dashes. Numeric variables will be listed generally with the same number of decimal places as in the actual data.

The following rules on decimal places will be considered in the listings for the derived variables (in the analyses approximations will not be performed):

- Age (years), extent of exposure (days): 0 decimal place;
- AUC/width: 2 decimal places;
- change from baseline: same as the variable considered.

The following rules on decimal places will be considered for the results of the analyses (if the analyses are performed on derived variables, the level of precision of the actual data is derived from the previous list):

- Min, max: same as actual data;
- Mean and its confidence limits (unadjusted and adjusted), adjusted difference between means and its confidence limits, SD, median: actual data + 1 decimal;
- Percentage: 1 decimal place.

9.3 Tables

The list of tables is provided here below:

TABLE	TITLE	OPTIONAL	KFR
TABLE T14.1-1	DISPOSITION OF PATIENTS / SUMMARY OF ALL PATIENTS		
TABLE T14.1-2	PRIMARY REASONS FOR DISCONTINUATION FROM THE STUDY / RANDOMISED SET		
TABLE T14.1-3	POPULATIONS FOR ANALYSIS / RANDOMISED SET		
TABLE T14.1-4	DEMOGRAPHIC AND BASELINE CHARACTERISTICS / SAFETY POPULATION		X
TABLE T14.1-4.1	DEMOGRAPHIC AND BASELINE CHARACTERISTICS / CAUCASIAN GENETIC PROFILE 2 / SAFETY POPULATION	X	
TABLE T14.1-4.2	DEMOGRAPHIC AND BASELINE CHARACTERISTICS / CHINESE GENETIC PROFILE 2 / SAFETY POPULATION	X	

TABLE T14.1-5.1	PAST DISEASES / SAFETY SET		
TABLE T14.1-5.2	CONCOMITANT DISEASES / SAFETY SET		
TABLE T14.1-5.2.1	CONCOMITANT DISEASES / CAUCASIAN GENETIC PROFILE 2 / SAFETY SET	X	
TABLE T14.1-5.2.2	CONCOMITANT DISEASES / CHINESE GENETIC PROFILE 2 / SAFETY SET	X	
TABLE T14.1-6.1	PREVIOUS MEDICATIONS / SAFETY SET		
TABLE T14.1-6.2	CONCOMITANT MEDICATIONS / SAFETY SET		
TABLE T14.1-6.2.1	CONCOMITANT MEDICATIONS / CAUCASIAN GENETIC PROFILE 2 / SAFETY SET	X	
TABLE T14.1-6.2.2	CONCOMITANT MEDICATIONS / CHINESE GENETIC PROFILE 2 / SAFETY SET	X	
TABLE T14.1-7	EXPOSURE AND COMPLIANCE TO STUDY DRUG / SAFETY SET		
TABLE T14.1-7.1	EXPOSURE AND COMPLIANCE TO STUDY DRUG / CAUCASIAN GENETIC PROFILE 2 / SAFETY SET	X	
TABLE T14.1-7.2	EXPOSURE AND COMPLIANCE TO STUDY DRUG / CHINESE GENETIC PROFILE 2 / SAFETY SET	X	
TABLE T14.2-1.1.1.2	PRIMARY EFFICACY ENDPOINT – PRIMARY ANALYSIS: OFFICE MEASUREMENT OF SITTING SYSTOLIC BLOOD PRESSURE DURING THE STUDY / GENETIC PROFILE 2 / PER PROTOCOL SET		X
TABLE T14.2-1.1.2.2	PRIMARY EFFICACY ENDPOINT – PRIMARY ANALYSIS: ANALYSIS OF OFFICE SBP CHANGE FROM BASELINE TO VISIT 6 / ANALYSIS OF COVARIANCE MODEL / GENETIC PROFILE 2 / PER PROTOCOL SET		X
TABLE T14.2-1.1.3.2	PRIMARY ANALYSIS: RESPONDERS AT VISIT 6 / GENETIC PROFILE 2 / PER PROTOCOL SET		X
TABLE T14.2-1.2.1.2	PRIMARY EFFICACY ENDPOINT – PRIMARY ANALYSIS: OFFICE MEASUREMENT OF SITTING SYSTOLIC BLOOD PRESSURE DURING THE STUDY / GENETIC PROFILE 1 (OVERALL SAMPLE) / PER PROTOCOL SET	X	
TABLE T14.2-1.2.2.2	PRIMARY EFFICACY ENDPOINT – PRIMARY ANALYSIS: ANALYSIS OF OFFICE SBP CHANGE FROM BASELINE TO VISIT 6 / ANALYSIS OF COVARIANCE MODEL / GENETIC PROFILE 1 (OVERALL SAMPLE) / PER PROTOCOL SET	X	
T14.2-1.2.3.2	PRIMARY EFFICACY ENDPOINT – PRIMARY ANALYSIS: RESPONDERS AT VISIT 6 / GENETIC PROFILE 1 (OVERALL SAMPLE) / PER PROTOCOL SET	X	

TABLE T14.2-1.3.1.2	PRIMARY EFFICACY ENDPOINT – SUBGROUP ANALYSIS: OFFICE MEASUREMENT OF SITTING SYSTOLIC BLOOD PRESSURE DURING THE STUDY / GENETIC PROFILE 3 / PER PROTOCOL SET	X	
TABLE T14.2-1.3.2.2	PRIMARY EFFICACY ENDPOINT – SUBGROUP ANALYSIS: ANALYSIS OF OFFICE SBP CHANGE FROM BASELINE TO VISIT 6 / ANALYSIS OF COVARIANCE MODEL / GENETIC PROFILE 3 / PER PROTOCOL SET	X	
T14.2-1.3.3.2	PRIMARY EFFICACY ENDPOINT – SUBGROUP ANALYSIS: RESPONDERS AT VISIT 6 / GENETIC PROFILE 3 / PER PROTOCOL SET	X	
TABLE T14.2-1.4.1.2	PRIMARY EFFICACY ENDPOINT – SECONDARY ANALYSIS: SENSITIVITY ANALYSIS OF OFFICE SBP CHANGE FROM BASELINE TO VISIT 6 BY ADDING THE LEVEL OF SODIUM AT VISIT 3 AS COVARIATE / ANALYSIS OF COVARIANCE MODEL / GENETIC PROFILE 2 / PER PROTOCOL SET	X	
TABLE T14.2-1.4.2.2	PRIMARY EFFICACY ENDPOINT – SECONDARY ANALYSIS: SENSITIVITY ANALYSIS OF OFFICE SBP CHANGE FROM BASELINE TO VISIT 6 BY ADDING TEMPERATURE CHANGE AS COVARIATE / ANALYSIS OF COVARIANCE MODEL / GENETIC PROFILE 2 / PER PROTOCOL SET	X	
TABLE T14.2-1.5.1.2	PRIMARY EFFICACY ENDPOINT – SECONDARY ANALYSIS: SENSITIVITY ANALYSIS OF OFFICE SBP CHANGE FROM BASELINE TO VISIT 6 BY ADDING THE LEVEL OF SODIUM AT VISIT 3 AS COVARIATE / ANALYSIS OF COVARIANCE MODEL / GENETIC PROFILE 1 (OVERALL SAMPLE) / PER PROTOCOL SET	X	
TABLE T14.2-1.5.2.2	PRIMARY EFFICACY ENDPOINT – SECONDARY ANALYSIS: SENSITIVITY ANALYSIS OF OFFICE SBP CHANGE FROM BASELINE TO VISIT 6 BY ADDING TEMPERATURE CHANGE AS COVARIATE / ANALYSIS OF COVARIANCE MODEL / GENETIC PROFILE 1 (OVERALL SAMPLE) / PER PROTOCOL SET	X	
TABLE T14.2-2.1.1.2	OFFICE MEASUREMENT OF SITTING DIASTOLIC BLOOD PRESSURE DURING THE STUDY / GENETIC PROFILE 2 / PER PROTOCOL SET	X	
TABLE T14.2-2.1.2.2	ANALYSIS OF OFFICE DBP CHANGE FROM BASELINE TO VISIT 6 / ANALYSIS OF COVARIANCE MODEL / GENETIC PROFILE 2 / PER PROTOCOL SET	X	
TABLE T14.2-2.2.1.2	OFFICE MEASUREMENT OF SITTING DIASTOLIC BLOOD PRESSURE DURING THE STUDY / GENETIC PROFILE 1 (OVERALL SAMPLE) / PER PROTOCOL SET	X	
TABLE T14.2-2.2.2.2	ANALYSIS OF OFFICE DBP CHANGE FROM BASELINE TO VISIT 6 / ANALYSIS OF COVARIANCE MODEL / GENETIC PROFILE 1 (OVERALL SAMPLE) / PER PROTOCOL SET	X	
TABLE T14.2-4.1.1.2	24-HOURS SYSTOLIC AND DIASTOLIC BLOOD PRESSURE MONITORING: DAY-TIME WEIGHTED MEAN VALUES / GENETIC PROFILE 2 / PER PROTOCOL SET	X	
TABLE T14.2-	ANALYSIS OF 24 HOURS SYSTOLIC AND DIASTOLIC BLOOD PRESSURE - DAY-TIME WEIGHTED MEAN VALUES: CHANGE	X	

4.1.2.2	FROM BASELINE TO VISIT 6-VISIT 7 / ANALYSIS OF COVARIANCE MODEL / GENETIC PROFILE 2 / PER PROTOCOL SET		
TABLE T14.2-4.2.1.2	24-HOURS SYSTOLIC AND DIASTOLIC BLOOD PRESSURE MONITORING: DAY-TIME WEIGHTED MEAN VALUES / GENETIC PROFILE 1 (OVERALL SAMPLE) / PER PROTOCOL SET	X	
TABLE T14.2-4.2.2.2	ANALYSIS OF 24 HOURS SYSTOLIC AND DIASTOLIC BLOOD PRESSURE - DAY-TIME WEIGHTED MEAN VALUES: CHANGE FROM BASELINE TO VISIT 6-VISIT 7 / ANALYSIS OF COVARIANCE MODEL / GENETIC PROFILE 1 (OVERALL SAMPLE) / PER PROTOCOL SET	X	
TABLE T14.2-5.1.1.2	24-HOURS SYSTOLIC AND DIASTOLIC BLOOD PRESSURE MONITORING: NIGHT-TIME WEIGHTED MEAN VALUES / GENETIC PROFILE 2 / PER PROTOCOL SET	X	
TABLE T14.2-5.1.2.2	ANALYSIS OF 24 HOURS SYSTOLIC AND DIASTOLIC BLOOD PRESSURE - NIGHT-TIME WEIGHTED MEAN VALUES: CHANGE FROM BASELINE TO VISIT 6-VISIT 7 / ANALYSIS OF COVARIANCE MODEL / GENETIC PROFILE 2 / PER PROTOCOL SET	X	
TABLE T14.2-5.2.1.2	24-HOURS SYSTOLIC AND DIASTOLIC BLOOD PRESSURE MONITORING: NIGHT-TIME WEIGHTED MEAN VALUES / GENETIC PROFILE 1 (OVERALL SAMPLE) / PER PROTOCOL SET	X	
TABLE T14.2-5.2.2.2	ANALYSIS OF 24 HOURS SYSTOLIC AND DIASTOLIC BLOOD PRESSURE - NIGHT-TIME WEIGHTED MEAN VALUES: CHANGE FROM BASELINE TO VISIT 6-VISIT 7 / ANALYSIS OF COVARIANCE MODEL / GENETIC PROFILE 1 (OVERALL SAMPLE) / PER PROTOCOL SET	X	
TABLE T14.3.1-1	SUMMARY OF TREATMENT-EMERGENT ADVERSE EVENTS / SAFETY SET		
TABLE T14.3.1-2	TREATMENT-EMERGENT ADVERSE EVENTS BY SYSTEM ORGAN CLASS AND PREFERRED TERM / SAFETY SET		
TABLE T14.3.5-1.1	LABORATORY PARAMETERS - HAEMATOLOGY: SHIFT TABLE FROM VISIT 3 TO VISIT 6 / SAFETY SET		
TABLE T14.3.5-1.2	LABORATORY PARAMETERS - BLOOD CHEMISTRY: SHIFT TABLE FROM VISIT 3 TO VISIT 6 / SAFETY SET		
TABLE T14.3.6-1	VITAL SIGNS DURING THE STUDY AND CHANGE FROM BASELINE / SAFETY SET		
TABLE T14.3.6-2.1	ECG: SHIFT TABLE FROM VISIT 3 TO VISIT 5 ON INVESTIGATOR’S INTERPRETATION / SAFETY SET		
TABLE T14.3.6-2.2	ECG: SHIFT TABLE FROM VISIT 3 TO VISIT 7 ON INVESTIGATOR’S INTERPRETATION / SAFETY SET		
TABLE T14.2-	PRIMARY EFFICACY ENDPOINT – PRIMARY ANALYSIS: OFFICE MEASUREMENT OF SITTING SYSTOLIC BLOOD		

1.1.1.1	PRESSURE DURING THE STUDY / GENETIC PROFILE 2 / FULL ANALYSIS SET (ITT APPROACH)		
TABLE T14.2-1.1.2.1	PRIMARY EFFICACY ENDPOINT – PRIMARY ANALYSIS: ANALYSIS OF OFFICE SBP CHANGE FROM BASELINE TO VISIT 6 / ANALYSIS OF COVARIANCE MODEL / GENETIC PROFILE 2 / FULL ANALYSIS SET (ITT APPROACH)		
TABLE T14.2-1.1.3.1	PRIMARY EFFICACY ENDPOINT – PRIMARY ANALYSIS: RESPONDERS AT VISIT 6 / GENETIC PROFILE 2 / FULL ANALYSIS SET (ITT APPROACH)		
TABLE T14.2-1.2.1.1	PRIMARY EFFICACY ENDPOINT – PRIMARY ANALYSIS: OFFICE MEASUREMENT OF SITTING SYSTOLIC BLOOD PRESSURE DURING THE STUDY / GENETIC PROFILE 1 (OVERALL SAMPLE) / FULL ANALYSIS SET (ITT APPROACH)		
TABLE T14.2-1.2.2.1	PRIMARY EFFICACY ENDPOINT – PRIMARY ANALYSIS: ANALYSIS OF OFFICE SBP CHANGE FROM BASELINE TO VISIT 6 / ANALYSIS OF COVARIANCE MODEL / GENETIC PROFILE 1 (OVERALL SAMPLE) / FULL ANALYSIS SET (ITT APPROACH)		
T14.2-1.2.3.1	PRIMARY EFFICACY ENDPOINT – PRIMARY ANALYSIS: RESPONDERS AT VISIT 6 / GENETIC PROFILE 1 (OVERALL SAMPLE) / FULL ANALYSIS SET (ITT APPROACH)		
TABLE T14.2-1.3.1.1	PRIMARY EFFICACY ENDPOINT – SUBGROUP ANALYSIS: OFFICE MEASUREMENT OF SITTING SYSTOLIC BLOOD PRESSURE DURING THE STUDY / GENETIC PROFILE 3 / FULL ANALYSIS SET (ITT APPROACH)		
TABLE T14.2-1.3.2.1	PRIMARY EFFICACY ENDPOINT – SUBGROUP ANALYSIS: ANALYSIS OF OFFICE SBP CHANGE FROM BASELINE TO VISIT 6 / ANALYSIS OF COVARIANCE MODEL / GENETIC PROFILE 3 / FULL ANALYSIS SET (ITT APPROACH)		
T14.2-1.3.3.1	PRIMARY EFFICACY ENDPOINT – SUBGROUP ANALYSIS: RESPONDERS AT VISIT 6 / GENETIC PROFILE 3 / FULL ANALYSIS SET (ITT APPROACH)		
TABLE T14.2-1.4.1.1	PRIMARY EFFICACY ENDPOINT – SECONDARY ANALYSIS: SENSITIVITY ANALYSIS OF OFFICE SBP CHANGE FROM BASELINE TO VISIT 6 BY ADDING THE LEVEL OF SODIUM AT VISIT 3 AS COVARIATE / ANALYSIS OF COVARIANCE MODEL / GENETIC PROFILE 2 / FULL ANALYSIS SET (ITT APPROACH)		
TABLE T14.2-1.4.2.1	PRIMARY EFFICACY ENDPOINT – SECONDARY ANALYSIS: SENSITIVITY ANALYSIS OF OFFICE SBP CHANGE FROM BASELINE TO VISIT 6 BY ADDING TEMPERATURE CHANGE AS COVARIATE / ANALYSIS OF COVARIANCE MODEL / GENETIC PROFILE 2 / FULL ANALYSIS SET (ITT APPROACH)	X	
TABLE T14.2-1.5.1.1	PRIMARY EFFICACY ENDPOINT – SECONDARY ANALYSIS: SENSITIVITY ANALYSIS OF OFFICE SBP CHANGE FROM BASELINE TO VISIT 6 BY ADDING THE LEVEL OF SODIUM AT VISIT 3 AS COVARIATE / ANALYSIS OF COVARIANCE MODEL / GENETIC PROFILE 1 (OVERALL SAMPLE) / FULL ANALYSIS SET (ITT APPROACH)		
TABLE T14.2-	PRIMARY EFFICACY ENDPOINT – SECONDARY ANALYSIS: SENSITIVITY ANALYSIS OF OFFICE SBP CHANGE FROM	X	

1.5.2.1	BASELINE TO VISIT 6 BY ADDING TEMPERATURE CHANGE AS COVARIATE / ANALYSIS OF COVARIANCE MODEL / GENETIC PROFILE 1 (OVERALL SAMPLE) / FULL ANALYSIS SET (ITT APPROACH)		
TABLE T14.2-1.6.1.1	PRIMARY EFFICACY ENDPOINT – SUBGROUP ANALYSIS: OFFICE MEASUREMENT OF SITTING SYSTOLIC BLOOD PRESSURE DURING THE STUDY / CAUCASIAN GENETIC PROFILE 2 / FULL ANALYSIS SET (ITT APPROACH)		
TABLE T14.2-1.6.1.2	PRIMARY EFFICACY ENDPOINT – SUBGROUP ANALYSIS: OFFICE MEASUREMENT OF SITTING SYSTOLIC BLOOD PRESSURE DURING THE STUDY / CAUCASIAN GENETIC PROFILE 2 / PER PROTOCOL SET		X
TABLE T14.2-1.6.2.1	PRIMARY EFFICACY ENDPOINT – SUBGROUP ANALYSIS: ANALYSIS OF OFFICE SBP CHANGE FROM BASELINE TO VISIT 6 / ANALYSIS OF COVARIANCE MODEL / CAUCASIAN GENETIC PROFILE 2 / FULL ANALYSIS SET (ITT APPROACH)		
TABLE T14.2-1.6.2.2	PRIMARY EFFICACY ENDPOINT – SUBGROUP ANALYSIS: ANALYSIS OF OFFICE SBP CHANGE FROM BASELINE TO VISIT 6 / ANALYSIS OF COVARIANCE MODEL / CAUCASIAN GENETIC PROFILE 2 / PER PROTOCOL SET		X
TABLE T14.2-1.6.3.1	PRIMARY EFFICACY ENDPOINT – SUBGROUP ANALYSIS: RESPONDERS AT VISIT 6 / CAUCASIAN GENETIC PROFILE 2 / FULL ANALYSIS SET (ITT APPROACH)		
TABLE T14.2-1.6.3.2	PRIMARY EFFICACY ENDPOINT – SUBGROUP ANALYSIS: RESPONDERS AT VISIT 6 / CAUCASIAN GENETIC PROFILE 2 / PER PROTOCOL SET		X
TABLE T14.2-1.7.1.1	PRIMARY EFFICACY ENDPOINT – SUBGROUP ANALYSIS: OFFICE MEASUREMENT OF SITTING SYSTOLIC BLOOD PRESSURE DURING THE STUDY / CHINESE GENETIC PROFILE 2 / FULL ANALYSIS SET (ITT APPROACH)		
TABLE T14.2-1.7.1.2	PRIMARY EFFICACY ENDPOINT – SUBGROUP ANALYSIS: OFFICE MEASUREMENT OF SITTING SYSTOLIC BLOOD PRESSURE DURING THE STUDY / CHINESE GENETIC PROFILE 2 / PER PROTOCOL SET		X
TABLE T14.2-1.7.2.1	PRIMARY EFFICACY ENDPOINT – SUBGROUP ANALYSIS: ANALYSIS OF OFFICE SBP CHANGE FROM BASELINE TO VISIT 6 / ANALYSIS OF COVARIANCE MODEL / CHINESE GENETIC PROFILE 2 / FULL ANALYSIS SET (ITT APPROACH)		
TABLE T14.2-1.7.2.2	PRIMARY EFFICACY ENDPOINT – SUBGROUP ANALYSIS: ANALYSIS OF OFFICE SBP CHANGE FROM BASELINE TO VISIT 6 / ANALYSIS OF COVARIANCE MODEL / CHINESE GENETIC PROFILE 2 / PER PROTOCOL SET		X
TABLE T14.2-1.7.3.1	PRIMARY EFFICACY ENDPOINT – SUBGROUP ANALYSIS: RESPONDERS AT VISIT 6 / CHINESE GENETIC PROFILE 2 / FULL ANALYSIS SET (ITT APPROACH)		
TABLE T14.2-1.7.3.2	PRIMARY EFFICACY ENDPOINT – SUBGROUP ANALYSIS: RESPONDERS AT VISIT 6 / CHINESE GENETIC PROFILE 2 / PER PROTOCOL SET		X
TABLE T14.2-	OFFICE MEASUREMENT OF SITTING DIASTOLIC BLOOD PRESSURE DURING THE STUDY / GENETIC PROFILE 2 / FULL		

2.1.1.1	ANALYSIS SET (ITT APPROACH)		
TABLE T14.2-2.1.2.1	ANALYSIS OF OFFICE DBP CHANGE FROM BASELINE TO VISIT 6 / ANALYSIS OF COVARIANCE MODEL / GENETIC PROFILE 2 / FULL ANALYSIS SET (ITT APPROACH)		
TABLE T14.2-2.2.1.1	OFFICE MEASUREMENT OF SITTING DIASTOLIC BLOOD PRESSURE DURING THE STUDY / GENETIC PROFILE 1 (OVERALL SAMPLE) / FULL ANALYSIS SET (ITT APPROACH)		
TABLE T14.2-2.2.2.1	ANALYSIS OF OFFICE DBP CHANGE FROM BASELINE TO VISIT 6 / ANALYSIS OF COVARIANCE MODEL / GENETIC PROFILE 1 (OVERALL SAMPLE) / FULL ANALYSIS SET (ITT APPROACH)		
TABLE T14.2-3.1.1.1	24-HOURS SYSTOLIC AND DIASTOLIC BLOOD PRESSURE MONITORING: 24 HOURS WEIGHTED MEAN VALUES / GENETIC PROFILE 2 / FULL ANALYSIS SET (ITT APPROACH)	X	
TABLE T14.2-3.1.1.2	24-HOURS SYSTOLIC AND DIASTOLIC BLOOD PRESSURE MONITORING: 24 HOURS WEIGHTED MEAN VALUES / GENETIC PROFILE 2 / PER PROTOCOL SET	X	
TABLE T14.2-3.1.2.1	ANALYSIS OF 24 HOURS SYSTOLIC AND DIASTOLIC BLOOD PRESSURE WEIGHTED MEAN VALUES: CHANGE FROM BASELINE TO VISIT 6-VISIT 7 / ANALYSIS OF COVARIANCE MODEL / GENETIC PROFILE 2 / FULL ANALYSIS SET (ITT APPROACH)	X	
TABLE T14.2-3.1.2.2	ANALYSIS OF 24 HOURS SYSTOLIC AND DIASTOLIC BLOOD PRESSURE WEIGHTED MEAN VALUES: CHANGE FROM BASELINE TO VISIT 6-VISIT 7 / ANALYSIS OF COVARIANCE MODEL / GENETIC PROFILE 2 / PER PROTOCOL SET	X	
TABLE T14.2-3.2.1.1	24-HOURS SYSTOLIC AND DIASTOLIC BLOOD PRESSURE MONITORING: 24 HOURS WEIGHTED MEAN VALUES / GENETIC PROFILE 1 (OVERALL SAMPLE) / FULL ANALYSIS SET (ITT APPROACH)	X	
TABLE T14.2-3.2.1.2	24-HOURS SYSTOLIC AND DIASTOLIC BLOOD PRESSURE MONITORING: 24 HOURS WEIGHTED MEAN VALUES / GENETIC PROFILE 1 (OVERALL SAMPLE) / PER PROTOCOL SET	X	
TABLE T14.2-3.2.2.1	ANALYSIS OF 24 HOURS SYSTOLIC AND DIASTOLIC BLOOD PRESSURE WEIGHTED MEAN VALUES: CHANGE FROM BASELINE TO VISIT 6-VISIT 7 / ANALYSIS OF COVARIANCE MODEL / GENETIC PROFILE 1 (OVERALL SAMPLE) / FULL ANALYSIS SET (ITT APPROACH)	X	
TABLE T14.2-3.2.2.2	ANALYSIS OF 24 HOURS SYSTOLIC AND DIASTOLIC BLOOD PRESSURE WEIGHTED MEAN VALUES: CHANGE FROM BASELINE TO VISIT 6-VISIT 7 / ANALYSIS OF COVARIANCE MODEL / GENETIC PROFILE 1 (OVERALL SAMPLE) / PER PROTOCOL SET	X	
TABLE T14.2-4.1.1.1	24-HOURS SYSTOLIC AND DIASTOLIC BLOOD PRESSURE MONITORING: DAY-TIME WEIGHTED MEAN VALUES / GENETIC PROFILE 2 / FULL ANALYSIS SET (ITT APPROACH)		
TABLE T14.2-4.1.2.1	ANALYSIS OF 24 HOURS SYSTOLIC AND DIASTOLIC BLOOD PRESSURE - DAY-TIME WEIGHTED MEAN VALUES: CHANGE FROM BASELINE TO VISIT 6-VISIT 7 / ANALYSIS OF		

	COVARIANCE MODEL / GENETIC PROFILE 2 / FULL ANALYSIS SET (ITT APPROACH)		
TABLE T14.2-4.2.1.1	24-HOURS SYSTOLIC AND DIASTOLIC BLOOD PRESSURE MONITORING: DAY-TIME WEIGHTED MEAN VALUES / GENETIC PROFILE 1 (OVERALL SAMPLE) / FULL ANALYSIS SET (ITT APPROACH)		
TABLE T14.2-4.2.2.1	ANALYSIS OF 24 HOURS SYSTOLIC AND DIASTOLIC BLOOD PRESSURE - DAY-TIME WEIGHTED MEAN VALUES: CHANGE FROM BASELINE TO VISIT 6-VISIT 7 / ANALYSIS OF COVARIANCE MODEL / GENETIC PROFILE 1 (OVERALL SAMPLE) / FULL ANALYSIS SET (ITT APPROACH)		
TABLE T14.2-5.1.1.1	24-HOURS SYSTOLIC AND DIASTOLIC BLOOD PRESSURE MONITORING: NIGHT-TIME WEIGHTED MEAN VALUES / GENETIC PROFILE 2 / FULL ANALYSIS SET (ITT APPROACH)		
TABLE T14.2-5.1.2.1	ANALYSIS OF 24 HOURS SYSTOLIC AND DIASTOLIC BLOOD PRESSURE - NIGHT-TIME WEIGHTED MEAN VALUES: CHANGE FROM BASELINE TO VISIT 6-VISIT 7 / ANALYSIS OF COVARIANCE MODEL / GENETIC PROFILE 2 / FULL ANALYSIS SET (ITT APPROACH)		
TABLE T14.2-5.2.1.1	24-HOURS SYSTOLIC AND DIASTOLIC BLOOD PRESSURE MONITORING: NIGHT-TIME WEIGHTED MEAN VALUES / GENETIC PROFILE 1 (OVERALL SAMPLE) / FULL ANALYSIS SET (ITT APPROACH)		
TABLE T14.2-5.2.2.1	ANALYSIS OF 24 HOURS SYSTOLIC AND DIASTOLIC BLOOD PRESSURE - NIGHT-TIME WEIGHTED MEAN VALUES: CHANGE FROM BASELINE TO VISIT 6-VISIT 7 / ANALYSIS OF COVARIANCE MODEL / GENETIC PROFILE 1 (OVERALL SAMPLE) / FULL ANALYSIS SET (ITT APPROACH)		
TABLE T14.3.1-3	SERIOUS TREATMENT-EMERGENT ADVERSE EVENTS BY SYSTEM ORGAN CLASS AND PREFERRED TERM / SAFETY SET	X	
TABLE T14.3.1-4	TREATMENT-EMERGENT ADVERSE DRUG REACTIONS BY SYSTEM ORGAN CLASS AND PREFERRED TERM / SAFETY SET	X	
TABLE T14.3.1-5	TREATMENT-EMERGENT ADVERSE EVENTS LEADING TO DISCONTINUATION BY SYSTEM ORGAN CLASS AND PREFERRED TERM / SAFETY SET	X	
TABLE T14.3.1-6	TREATMENT-EMERGENT ADVERSE EVENTS LEADING TO DEATH BY SYSTEM ORGAN CLASS AND PREFERRED TERM / SAFETY SET	X	

9.4 Figures

Not foreseen.

9.5 Listings

The list of listings is provided here below:

LISTING	TITLE
LISTING 16.1.7	RANDOMISATION SCHEME AND CODES / RANDOMISED SET
LISTING 16.2.1-1	DISPOSITION OF PATIENTS / RANDOMISED SET
LISTING 16.2.1-2.1	SCREENING FAILURES / SCREENING FAILURE PATIENTS
LISTING 16.2.1-2.2	STUDY TERMINATION / RANDOMISED SET
LISTING 16.2.2-1	PROTOCOL VIOLATIONS / RANDOMISED SET
LISTING 16.2.2-2	VIOLATIONS OF INCLUSION/EXCLUSION CRITERIA / RANDOMISED SET
LISTING 16.2.3-1	POPULATIONS FOR ANALYSIS / RANDOMISED SET
LISTING 16.2.4-1	DEMOGRAPHICS AND BASELINE CHARACTERISTICS / RANDOMISED SET
LISTING 16.2.4-2	PREGNANCY TEST / RANDOMISED SET
LISTING 16.2.4-4	PHYSICAL EXAMINATIONS / RANDOMISED SET
LISTING 16.2.4-5	PAST-CONCOMITANT DISEASES / RANDOMISED SET
LISTING 16.2.5-1	MEDICATIONS / RANDOMISED SET
LISTING 16.2.5-2.1	STUDY DRUG DISPENSING AND ACCOUNTABILITY / RANDOMISED SET
LISTING 16.2.5-2.2	STUDY DRUG LOG / RANDOMISED SET
LISTING 16.2.5-2.2.2	STUDY TREATMENT INTERRUPTIONS / RANDOMIZED SET
LISTING 16.2.5-2.3	EXTENT OF EXPOSURE AND COMPLIANCE TO STUDY MEDICATION / RANDOMISED SET
LISTING 16.2.6-1	OFFICE MEASUREMENT OF SITTING SYSTOLIC AND DIASTOLIC BLOOD PRESSURE / RANDOMISED SET
LISTING 16.2.6-2	24-HOURS BLOOD PRESSURE MONITORING / RANDOMISED SET
LISTING 16.2.6-3	24-HOURS BLOOD PRESSURE MONITORING DERIVED VARIABLES: WEIGHTED MEAN VALUES (AUC/WIDTH) / RANDOMISED SET
LISTING 16.2.6-4	DIARY DATA / RANDOMISED SET
LISTING 16.2.6-5	TEMPERATURE DATA / RANDOMISED SET
LISTING 16.2.7-1	PRE-TREATMENT ADVERSE EVENTS / SUMMARY OF ALL PATIENTS
LISTING 16.2.7-2.1	TREATMENT-EMERGENT ADVERSE EVENTS / RANDOMISED SET
LISTING 16.2.8-1	VITAL SIGNS / RANDOMISED SET
LISTING 16.2.8-2	12-LEAD ECG / RANDOMISED SET
LISTING 16.2.8-3	LABORATORY PARAMETERS / RANDOMISED SET

10. Literature

- A. Lawrence Gould. *Planning and revising the sample size for a trial*. Statistics in Medicine. Volume 14, Issue 9, pages 1039–1051, 15/30 May 1995.
- CROS NT Standard Operating Procedure SOP ST03/V01 “Statistical Analysis Plans”;
- European Medicines Agency (EMEA), International Conference on Harmonisation (ICH) E3 Harmonised Guideline (1996) “Structure and Content of Clinical Study Reports”;
- EMEA, ICH E9 Harmonised Guideline (1998) “Statistical Principles for Clinical Trials”.

11. Appendices

See document “AppendixI_PST2238DM10001_CVTCV001_200418(Final_2.0).docx”.

11.1 Table shells

See document “AppendixI_PST2238DM10001_CVTCV001_200418(Final_2.0).docx”.

11.2 Figure shells

Figure shells not applicable for this study.

11.3 Listing shells

Listing shells not applicable for this study.
