# Supplementary material for: Antihypertensive treatment guided by genetics: PEARL-HT, the randomized proof-of-concept trial comparing rostafuroxin with losartan
Source: Pharmacogenomics J. 2021 Mar 1;21(3):346–58. doi: 10.1038/s41397-021-00214-y (PMC8159753; doi:10.1038/s41397-021-00214-y)
Supplement: Supplementary file 5 — Text S3 [file 41397_2021_214_MOESM5_ESM.pdf]

DATA REVIEW REPORT

Study Information	
Sponsor:	CVie Therapeutics Company Limited
Protocol Number:	PST 2238-DM-10-001 (Italy) / CVT-CV-001 (Taiwan)
Protocol Title:	ANTIHYPERTENSIVE EFFECT OF DIFFERENT DOSES OF ROSTAFUROXIN IN COMPARISON WITH LOSARTAN, ASSESSED BY OFFICE AND AMBULATORY BLOOD PRESSURE MONITORING IN A HYPERTENSIVE POPULATION SELECTED ACCORDING TO A SPECIFIC GENETIC PROFILE

Document Information	
Document Author:	Valeria Bandiera
Document Version:	2.0
Document Date:	*(24 May 2018)*

Lead Biostatistician details:	
Name:	Valeria Bandiera
Job Role:	Senior Biostatistician
Company:	CROS NT
Signature:	
Date of signature:	*(DD Mmm YYYY)*

Sponsor Approver details:	
Name:	Giuseppe Bianchi
Job Role:	Appointed Medical Expert
Company:	CVie Therapeutics Company Limited
Signature:	
Date of signature:	*(DD Mmm YYYY)*

Contents

1. Introduction 4
2. Subject Disposition 4
3. Protocol Violations 5
   3.1 Subjects Violating Inclusion / Exclusion Criteria 5
   3.2 Subjects with Protocol Violations During the Study 5
      3.2.1 Subjects with non-permitted medications 5
      3.2.2 Study Treatment number not assigned in chronological order 6
      3.2.3 Subjects who received a drug different from the one assigned by randomization 6
      3.2.4 Randomization code broken 6
      3.2.5 Exposure to treatment 7
      3.2.6 Non-compliance to study treatments 7
      3.2.7 Not respecting the visit schedule 11
      3.2.8 Other protocol violations during the study 11
4. Statistical Analysis Plan Review 11
   4.1 Primary Efficacy Variables 11
   4.2 Safety Variables 12
   4.3 Factors to be included in the models 12
5. Other Considerations 14
6. Populations for Analysis 15
   6.1 Analysis Populations 15
      6.1.1 Screened population 15
      6.1.2 Randomized population 15
      6.1.3 Safety population 16
      Subjects excluded from the safety population: 4 subjects. 16
      6.1.4 FAS population 16
      6.1.5 PP population 17
      6.1.6 Population Summary 20
7. Appendices 20

Version Number	Changes Made	Document Date
0.1	Draft specification based on the following documents:<br>- Study protocol (Final Version, 11July 2017);<br>- CRF (Version Final 2, 13 April 2015);<br>- SAP (Version Final 1.0, 15 January 2018);<br>- Data Review Listings (Version Draft 1, 03 April 2018).	16 Apr 2018
0.2	Version updated after the Sponsor review and based on the following updated documents:<br>- Data Review Listings (Version Draft 2, 17 April 2018).	19 Apr 2018
1.0	Final version confirmed after the Sponsor review.	20 Apr 2018
2.0	Final version with correction about FAS population and Final Protocol Violations appendix	24 May 2018

1. Introduction

The day April 05, 2018, at CROS NT s.r.l. (Verona) and via teleconference, the Data Review Meeting of the study PST 2238-DM-10-001 / CVT-CV-001 was held. According to the SOP OP04_V01, the purpose of the meeting was to make all the decisions, preliminarily to the database hard lock, necessary for the correct assignment of each patient populations defined in the Protocol (SAF, FAS, PPS), including Protocol Deviations (PDs) identification and categorization, as well as to review the statistical analysis planned, investigating its feasibility and identifying changes needed, and to take decisions on potential pending data issues.

Participants:

Valeria Bandiera, Senior Biostatistician, CROS NT

Marco Pannacci, Senior Biostatistician, CROS NT

Giuseppe Bianchi, Appointed Medical Expert, CVie Therapeutics Company Limited

All the participants were provided with the blind review listings (version: Draft 1, 03 April 2018).

2. Subject Disposition

The total number of screened patient was 902 and 623 patients withdrew from the study before randomisation.

The total number of randomised patient was 279.

253 patients completed the treatment period according to the protocol and 26 patients withdrew during the treatment period.

A total of 275 patients took at least one dose of study treatment.

A listing of screening failure subjects (Listing 16.2.1-2.1, appendix E, G) and a listing of subjects who withdrew from the study (Listing 16.2.1-2.2 , appendix E, G) are presented in the appendices.

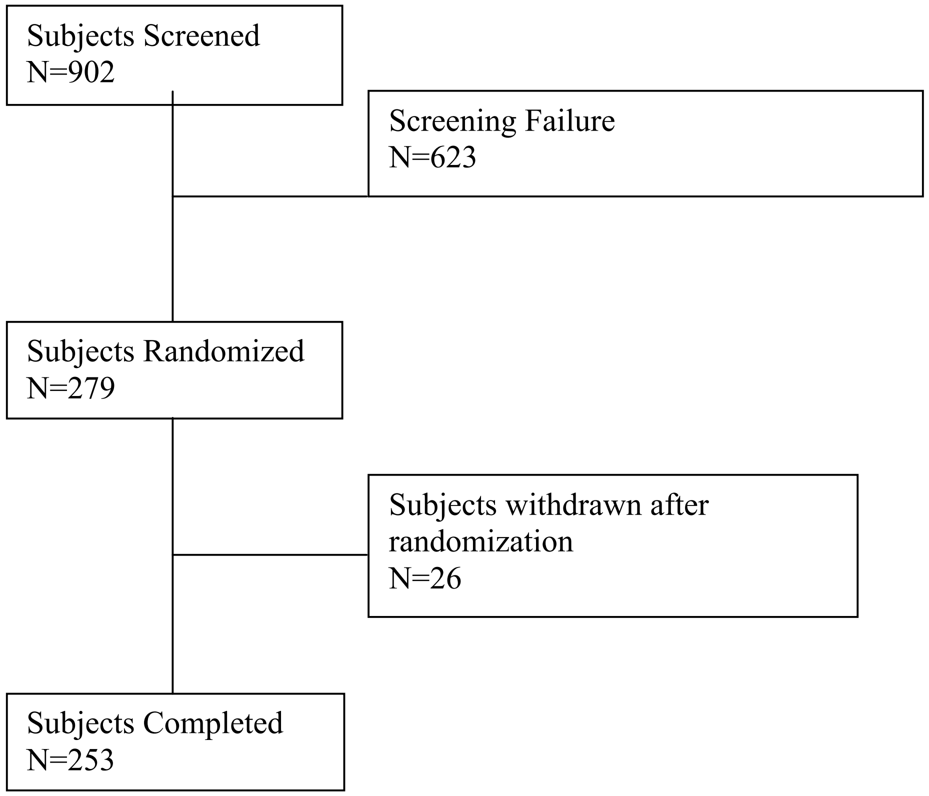

3. Protocol Violations

Review of data was performed on randomized subjects.

All violations will be summarised in a listing that includes Subject number, Deviation decode, Deviation term, Deviation category (No deviation, Minor, Major) and Comment (it will contain any relevant details to justify the deviation category).

The Sponsor will provide a final integrated version of all protocol violations (major as well as minor ones), including the below mentioned and any further minor deviation identified during medical monitoring activities.

3.1 Subjects Violating Inclusion / Exclusion Criteria

All violations of inclusion/exclusion criteria as reported in the listing 16.2.2-2 were reviewed and categorized as minor.

3.2 Subjects with Protocol Violations During the Study

3.2.1 Subjects with non-permitted medications

The Sponsor's Appointed Medical Expert reviewed the Listing 16.2.5-1 (appendix A) and identified the following patients who used non-permitted concomitant medications (as per section 7.5 of the clinical study protocol) to be considered of major impact:

**Subject ID**	**Deviation term**	**Deviation Decode**	**Deviation category**	**Comment**
10542	ANGIOTENSIN II ANTAGONISTS, PLAIN, C09CA/ CARDIOVASCULAR SYSTEM, C/ OLMESARTAN/ OLMESARTAN	Non-permitted medications	major	Medical judgment is that Olmesartan substantially contribute to the BP decrease.<br>The patient will be excluded from PPS

All other non-permitted medications identified have been confirmed as minor violations.

The following patients, considered as violators according to monitoring notes (appendix J) were further reviewed by the Sponsor after the DR meeting to confirm their deviation category and they were finally considered as minor violations: 20502, 20606, 20807, 21624, 20603.

3.2.2 Study Treatment number not assigned in chronological order

The randomization took place automatically and was never forced.

The Sponsor confirmed no issues regarding the study treatment number chronological assignation.

3.2.3 Subjects who received a drug different from the one assigned by randomization

None.

3.2.4 Randomization code broken

Randomization code was broken for the following subjects: 11303,11309,11313,10704 e 10801.

The blind was broken as a precaution for safety reasons, because of the development of mold in the 50 micro batch of capsules at the stability test batches.

Specifically, the random code was broken for subject 10704, on 27 November 2013, because the investigator considered as essential to know which treatment the patient is taking because of the emergency situation. The study eCRF documents the open of the blind only for this subject.

Subject 10801 already completed the study when the issue about mold was found, so no specific reporting on eCRF was done.

For the other three subjects (11303,11309,11313) only a comment was included in the End of Study eCRF page, justifying the forced drop-out from study per Sponsor decision.

Details of the aforementioned cases of randomization code broken will be included in the final Clinical Study Report.

3.2.5 Exposure to treatment

The extent of exposure (days) was calculated using the following formula:

Extent of exposure (days) = Date of last randomised study drug intake - Date of first randomised study drug intake +1.

During the Data Review Meeting, the Sponsor reviewed the first draft version of listing 16.2.5-2.1; this listing will be re-run in order to update compliance calculation (see section 3.2.6 below for details) and will be reviewed again to identify any potential issue.

3.2.6 Non-compliance to study treatments

Compliance to study drug was evaluated on the basis of the information recorded on the CRF.

On a per patient basis, the evaluation of the compliance during the treatment period was done using the following formula:

$$\text{Compliance}(\%) = \frac{\text{Total number of study drugs taken during the treatment period}}{\text{Total number of scheduled study drugs during the treatment period}} \times 100$$

In particular, the formula above was used to calculate overall compliance, by considering the following indications (as per Final 1.0 SAP Section 6.1):

Overall	Total number of	
	Study drugs taken	Scheduled study drugs
	Sum of the differences between "Number of capsules/tablets given to the patient" and "Number of capsules/tablets returned by the patient" by considering all the records reported in the "List of supplies administered to the patient" CRF form.	2 capsules/tablets x (Date of last last randomized study drug intake - Date of first randomized study medication intake +1).

In the CRF, the field "Number of capsules/tablets given to the patient" was pre-filled, so in the database drug accountability is present also for patients withdrawn prematurely from the study or who did not perform a scheduled visit and the corresponding field "Number of capsules/tablets returned by the patient" was not completed uniformly.

So, in the first draft version of listing 16.2.5-2.1 compliance calculation was done not considering records with unknown number of capsules/tablets returned by the patient as well as number of capsules/tablets returned by the patient equal to zero.

The listing was reviewed by the study team and the Data Management team was asked investigate the aforementioned potential data issue detected; in particular, the following patients with low

compliance, calculated with restrictions applied as above, were provided to the Lead Data Manager for specific check: 10702, 11315, 11327, 11342, 11401, 21504, 21614, 21624, 21627.

Cases not affected by such pending action were reviewed by the Sponsor and the following patients with confirmed compliance lower than 80% have been detected:

**Subject ID**	**Deviation Term**	**Deviation Decode**	**Deviation Category**	**Comment**
10904	Compliance at 75%	Poor medication compliance	Major	The patient will be excluded from PPS
21004	Compliance at 72%	Poor medication compliance	Major	The patient will be excluded from PPS
21430	Compliance at 80%	Poor medication compliance	Major	The patient will be excluded from PPS

In order to complete the compliance review, the listing 16.2.5-2.1 will be re-run, calculating compliance based on study treatment periods when effectively patients received the study medication only; such information, included in the study medication dispensing section (i.e. patients for which "Yes" has been reported in the study medication dispensing CRF section for question "Has study medication (for 1st Treatment Period Week 1-5) been dispensed to the patient" for the related treatment period) will be merged with Drug Accountability and used for final listing production. The use of study drug dispensing information as mentioned here above allows a correct calculation of compliance to study treatment for the statistical analysis; it's to be noted that the related listing will display exactly what was reported in the eCRF, so inconsistencies for the variable "No. of caps/tabs returned" will be also displayed, but they will not affect the analysis results anyhow.

The study team then reviewed the final version of the data review listing 16.2.5-2.1 and confirmed no further cases of poor compliance were identified.

Additionally, for the following patients the study drug compliance was not computable, because the "Number of capsules/tablets returned by the patient" is unknown (the patient lost the drug blister):

**Subject ID**	**Deviation Term**	**Deviation Decode**	**Deviation Category**	**Comment**
21624	Compliance missing	Compliance not computable	Major	The patient will be excluded from PPS
21627	Compliance missing	Compliance not computable	Major	The patient will be excluded from PPS
21614	Compliance missing	Compliance not computable	Major	The patient will be excluded from PPS

For the following subjects, study medication dispensing was not reported for the first treatment period in the eCRF but from Drug Accountability Form it's evident that the drug was taken for both

treatment periods, so, the answer "Yes" for "Has the medication been dispensed" will be forced (hardcoding) for statistical analysis programming purpose:

Subject ID	Week	No. of caps/tabs returned	No. of caps/tabs given	Extent of exposure (days)	Has study medication been dispensed?
10503	WEEK 1-5	4	45	72	MISSING
10503	WEEK 1-5	15	56	72	MISSING
10503	WEEK 6-9	14	45	72	Yes
10503	WEEK 6-9	25	56	72	Yes
10555	WEEK 1-5	7	45	69	MISSING
10555	WEEK 1-5	18	56	69	MISSING
10555	WEEK 6-9	16	45	69	Yes
10555	WEEK 6-9	27	56	69	Yes

Finally, during the blind review of the data the following cases with compliance higher than 110% were recognized and reasons of such result was investigated:

Subject ID	Week	No. of caps/tabs returned	No. of caps/tabs given	Extent of exposure (days)	Has study medication been dispensed?	Overall Compliance	Comments
10122	WEEK 1-5	9	45	9	Y	461%	Query done and data confirmed
10122	WEEK 1-5	9	56	9	Y		
10122	WEEK 6-9	45	45#	9			
10122	WEEK 6-9	56	56#	9			
10401	WEEK 1-5	24	45	14	Y	150%	No additional information available
10401	WEEK 1-5	35	56	14	Y		
10401	WEEK 6-9	0	45#	14			
10401	WEEK 6-9	0	56#	14			
10508	WEEK 1-5	41	45	3	Y	133%	No additional information available
10508	WEEK 1-5	52	56	3	Y		
10508	WEEK	.	45#	3			

	6-9						
10508	WEEK 6-9	.	56#	3			
10570	WEEK 1-5	8	45	64	Y	122%	Patient fails to return period 2 box with unused blisters
10570	WEEK 1-5	19	56	64	Y		
10570	WEEK 6-9	1	45	64	Y		
10570	WEEK 6-9	18	56	64	Y		
10589	WEEK 1-5	9	45	55	Y	118%	No additional information available
10589	WEEK 1-5	20	56	55	Y		
10589	WEEK 6-9	16	45	55	Y		
10589	WEEK 6-9	27	56	55	Y		
11315	WEEK 1-5	0	45	68	Y	124%	Patient declared the correct assumption of drug - period 1 but the whole box was thrown away by mistake
11315	WEEK 1-5	0	56	68	Y		
11315	WEEK 6-9	11	45	68	Y		
11315	WEEK 6-9	22	56	68	Y		
21626	WEEK 1-5	11	45	68	Y	111%	Rostafuroxin/placebo 4 capsules and Losartan/ placebo 19 tablets were missing and not returned by subject
21626	WEEK 1-5	22	56	68	Y		
21626	WEEK 6-9	11	45	68	Y		
21626	WEEK 6-9	7	56	68	Y		

The data, as included in the db, were confirmed by the Data Management and the Sponsor agreed to not proceed with any further action at site level for these cases.

Data review meeting decision: Patients 10904, 21004, 21430, 21624, 21627, 21614, have been identified as major violators due to poor or not computable medication compliance.

3.2.7 Not respecting the visit schedule

Violations regarding visit schedule time windows not respected were collected during monitoring activities and reported in the related protocol deviation listings (appendix I, J, K, L); these deviations were reviewed by the Sponsor and categorized as minor.

3.2.8 Other protocol violations during the study

As per Clinical Study Protocol, the second drug kit (for Treatment Period 2) should have been dispensed to patients at Visit 5, but subject 21504 this was dispensed at Visit 4 (on 05Oct2016) by mistake, which was reported as a protocol deviation. At Visit 4, drug kit regarding Treatment Period 1 (which was correctly dispensed at Visit 3 on 20Sep2016) was returned by subject and well stored in the fridge of our pharmacy. In order to ensure subject have sufficient study drug during whole study period, Covance Medical Monitor suggested to re-dispense the drug kit related to Treatment Period 1 at Visit 5 (31Oct2016).

Since the patient was finally provided with all the needed amount of drug to be able to correctly complete both treatment periods without any issue, this deviation can be considered as minor.

In order to calculate study drug compliance properly for this subject (see section 3.2.6), considering the dispensation was done at the wrong visit, study medication dispensing in the CRF, which actually reports "No" to question "Has study medication (for last Treatment Period) been dispensed to the patient?" was corrected to "Yes" (and date of Visit 4 will be included as date of dispensing) in order to not lost the information.

4. Statistical Analysis Plan Review

Review of data was performed on randomized subjects.

4.1 Primary Efficacy Variables

Primary efficacy endpoints are

- Change from baseline to Visit 6 in office sitting SBP.
- Proportions of responders at Visit 6, defined as “Patients having the mean office SBP (mean of last three measurements) <= 135 mmHg or having a reduction in mean office SBP (mean of the three last measurements) > or = 10% with respect to the baseline measurement” at Visit 6 (after two months of therapy).

No particular issues have been identified during the Data Review Meeting with regards to primary efficacy variables.
The blind review of the data revealed important differences between the two ethnic subpopulations (Caucasian and Chinese), in particular regarding the baseline SBP and the average SBP fall at visit 6 as well as genetic differences (gene pair frequencies); so, the inclusion of the country as factor in

the statistical model for the analysis of the primary efficacy variable (see also section 4.3 below) as well as the analysis by subgroup were confirmed.
Considerations on genetic confounding factors for primary analysis were done and the definition of PPS was revised accordingly (see section 6.1.5 below).

4.2 Safety Variables

The safety endpoints are the following:

- Physical examinations.
- ECG.
- Vital signs.
- Laboratory tests.
- Adverse events.
- Concomitant medications.

No particular issues have been identified during the Data Review Meeting with regards to safety efficacy variables.
Reviewing medications, the Sponsor recognized a considerable difference in medications taken by patients belonging to the Italian and Chinese subpopulations. As a result of such consideration, some optional subgroup analysis on baseline characteristics will be included, as optional, in the Final 2.0 SAP.

4.3 Factors to be included in the models

In order to compensate for any imbalance between groups due to the possible relationship between values measured after treatment and baseline, all ANCOVA models used for the analysis will include the baseline value of the dependent variable as covariate and treatment, country and treatment x country as factors.

The baseline used in the statistical analysis for each variable is reported in the table below:

**Endpoint**	**Baseline**
Office measurement of systolic and diastolic blood pressure (SBP/DBP)	Visit 3(if available)*
24-Hours Systolic and Diastolic Blood Pressure Monitoring: 24 hours, day-time and night-time weighted mean values	Visit 2-Visit 3
Laboratory parameters (Haematology, Blood Chemistry and Urinalysis)	Visit 3
Vital signs	Visit 3(if available)*

ECG	Visit 3

*Baseline measurement is Visit 3 if available, otherwise if the baseline measurement is missing but there is a prior measurement recorded at Visit 2, this value will be carried forward and used as the baseline measurement.

The blind review of the data revealed a considerable variation among the frequencies of the four gene-pairs of the profile 2 much greater than what was observed in the previous OASIS trial. Therefore the Sponsor required the integration in the study database of a new variable including specific gene pair information; such data, which are actually not part of the planned data collection, are available to the Sponsor from the Division of Nephrology and Hypertension (University of Milan - San Raffaele Hospital, Milan – ITALY).These external data will be provided to CROS NT for all randomized patients belonging to genetic profile 2 and will be imported into the analysis datasets for the statistical analysis (appendix M).

The study team discussed the possibility to use the aforementioned new variable as additional factor in the models for primary efficacy analysis on FAS (genetic profile 2 subgroup only) and finally considered useful this approach. However, after the DR meeting, the frequencies of levels of such variable were investigated with the following observed results:

Table 1 Frequencies of gene pair variable levels in the FAS population, genetic profile 2 subgroup

**Gene Pairs**	**IT (N=89)**
ADD1/ADD3	Yes 10 (11.2%)
ADD1/ADD3-MDR1/HSD18	1 (1.1%)
ADD1/ADD3-MDR1/LSS	2 (2.2%)
ADD1/ADD3-MDR1/LSS-ADD1/LSS	1 (1.1%)
ADD1/LSS	5 (5.6%)
MDR1/HSD18	Yes 16 (18.0%)
MDR1/LSS	51 (57.3%)
MDR1/LSSADD1/LSS	1 (1.1%)
MDR1/LSS-ADD1/LSS	2 (2.2%)
**Gene Pairs**	**TW (N=54)**
ADD1/ADD3	9 (16.7%)
ADD1/ADD3-MDR1/LSS	4 (7.4%)

ADD1/LSS	5 (9.3%)
MDR1/HSD3B1	3 (5.6%)
MDR1/LSS	26 (48.1%)
MDR1/LSS-ADD1/LSS	7 (13.0%)

Due to some gene pair representatives with low numerosity, from a statistical point of view it was deemed necessary to agree on some levels aggregation according to clinical judgment, if any is possible.

However, the Sponsor finally considered that a valid scientific basis (rational) for this gene pair aggregation cannot be provided, thus the new variable will not be included as factor in any statistical model and it will be used only for potential patients exclusion from PPS, as explained in section 6.1.5 below.

5. Other Considerations

During the DR meeting, the Sponsor provided the following considerations about 24 h ABPM extreme values:

whether 24 h ABPM readings with outliers or missing values should be rejected and not considered for data analyses has been matter of controversy for years. Some studies have shown that the data editing process may not influence the calculation of average BP values in large groups or populations or subjects, while in small groups and even more so in individual patients, it may mainly affect the calculation of the average level of systolic BP and the standard deviation of both systolic and diastolic BP. Different editing procedures have been adopted by many authors, but none could be demonstrated as being superior to the others.

As we are dealing with small size subsets of naive patients with mild, stage 1, hypertension, to remove patients from the analysis, the following four main clinical criteria have been applied, according to the protocol:

A) Less than 10 readings between 10.00 and 20.00 hours.
B) Less than 5 readings between midnight and 06.00 in the morning
C) The recording cover less than 20 hours
D) No valid readings are available for 3 or more hours

After a further inspection of the blind data the following were also be added:

D1) when the difference between the extreme values of the single readings of one hour is above 50 mmHg, the corresponding mean value cannot be valid

E) As we are measuring the antihypertensive effect of the study drugs in naïve mild hypertensive patients at office BP, patients with SBP in the low range of the normal values at the basal visit 2 have to be excluded

As a result of the aforementioned Sponsor's review and evaluation of extreme values of 24-Hours Systolic and Diastolic Blood Pressure Monitoring, the following patients will be finally excluded from this secondary efficacy analysis:

Subject ID	Comment
10518	Patient excluded according to above criterion C
11339	Patient excluded according to above criteria C and E
105106	Patient excluded according to above criterion E
105112	Patient excluded according to above criterion C
105222	Patient excluded according to above criterion C
10734	Patient excluded according to above criteria E and C
10502	Patient excluded according to above criterion C
10531	Patient excluded according to above criterion C
11004	Patient excluded according to above criterion C and E
10590	Patient excluded according to above criterion C
11350	Patient excluded according to above criterion C
105159	Patient excluded according to above criterion C
105187	Patient excluded according to above criterion C
10711	Patient excluded according to above criterion C
21632	Patient not evaluable, since performed only visit 2
21626	Patient excluded according to above criterion C
20202	Patient excluded according to above criterion C
20216	Patient excluded according to above criterion C
21443	Patient excluded according to above criterion C
21004	Patient not evaluable, since performed only visit 2
20904	Patient excluded according to above criterion C
20111	Patient excluded according to above criteria E and C
21312	Patient excluded according to above criterion C

Also, possible site pooling was discussed during the DR meeting. The Sponsor emphasized the main interest in ethnic subgroup investigations and, since country was already included as factor in the analysis as well as some subgroup analysis (for Italian and Chinese ethnicity) was already planned (as optional) in the SAP, the Sponsor did not consider needed any particular pooling of sites.

6. Populations for Analysis

6.1 Analysis Populations

6.1.1 Screened population

Screened population: 902 subjects.

6.1.2 Randomized population

Randomized population: 279 subjects.

6.1.3 Safety population

The Safety Analysis Set (SAF) includes all randomised patients who receive at least one dose of the study drug.

Listing 8.1 (appendix A) about randomized patients who did not take any study medication was reviewed and the following subjects were confirmed to be excluded from SAF:

Subject ID	Comment
105174	The subject was not treated
20529	The subject withdrew the consent and left the study without taking any study drug.

However, during the DR, a data issue was also recognized for the following patients, who were not treated according to drug accountability information but a date of first drug intake was wrongly included in the db:

Subject ID	Comment
21632	Date of first drug intake, 28SEP2017, but the subject was effectively never treated according to drug accountability information available
21643	Date of first drug intake, 20OCT2017, but the subject was effectively never treated according to drug accountability information available

Such data issue was reported to Data Management team for correction and the listing 8.1 was finally re-produced (appendix E, G), showing all the aforementioned subjects exclusions from SAF.

Safety population: 275 subjects.

Subjects excluded from the safety population: 4 subjects.

6.1.4 FAS population

The definition of the Full Analysis Set (FAS) included in the SAP Final 1.0, 15 Jan 2018, is:

The FAS includes all randomised patients who receive at least one dose of the study drug and have at least one post-baseline efficacy assessment at visit 5 or visit 6.

All the treated patients without any post-baseline efficacy evaluation at visit 5 or visit 6, as included in listing 8.2 (appendix A), were reviewed during the Data Review meeting and confirmed to be excluded from FAS, according to the above definition.

However, after study team re-discussion on ITT principle compliance and protocol alignment, it was agreed to change the FAS definition previously included in the SAP with the following:

The FAS includes all randomised patients who receive at least one dose of the study drug and have at least one post-baseline efficacy assessment.

Restriction to visit 5 or visit 6 for post-baseline efficacy assessments will be included in the per-protocol set definition.

As a consequence of the modified FAS definition, listing 8.2 (appendix A) has been modified (new listing will be 8.3 included in Appendix C, E, G) in order to identify only treated patients without any post-baseline efficacy assessment, who should be finally excluded from FAS.

So, the patients finally confirmed to be excluded from FAS are the following:

Subject ID	Comment
105174	The subject was not treated
20529	The subject withdrew the consent and left the study without taking any study drug.
21632	The subject was not treated
21643	The subject was not treated
10542	The subject has no post-baseline efficacy assessments

FAS population: 274 subjects.

Subjects excluded from the FAS population: 5 subjects.

6.1.5 PP population

The definition of the Per-protocol Set included in the SAP Final 1.0, 15 Jan 2018, is:

The Per-protocol Set (PPS) includes all randomised patients of the FAS who fulfil the eligibility criteria of the Protocol, perform the Visit at the end of the Treatment Period, complete the treatment with a compliance of at least 80%, don't take prohibited drugs during the course of the Study and don't discontinue the Study Drug treatment during the day before or the day of each scheduled Visit.

During the Data Review Meeting was evaluated if a patient interrupting the Study because of uncontrolled hypertension (i.e. office SBP ≥ 179 mmHg or DBP ≥ 110 mmHg) should be included or not in the statistical analysis with his/her last blood pressure evaluation.

The following cases were reviewed:

Subject ID	Reason of Treatment Withdrawal	Sponsor comment
11364	OTHER: UNCONTROLLED HYPERTENSION	Mean of sitting blood pressure values at visit 5 is 165,3-98.7 mmHg
10514	OTHER: UNCONTROLLED HYPERTENSION	Mean of sitting blood pressure values at visit 4 is 172-108.3 mmHg
10401	OTHER: UNCONTROLLED HYPERTENSION	Mean of sitting blood pressure values at visit 4 is 167.7-115.7 mmHg
10122	OTHER: UNCONTROLLED HYPERTENSION	Mean of sitting blood pressure values at visit 5 is171,7-105,7 mmHg

Data review meeting decision: all the aforementioned patients, withdrew from study due to uncontrolled hypertension, will be excluded from the PPS as all other discontinued patients.

Violations of eligibility criteria, prohibited drugs, discontinuations and cases with low compliance to the study medication (less than 80%) were reviewed as reported in the protocol violations section 3.2 here above and the following patients were confirmed to be excluded from the PPS:

Subject ID	Comment
10102	The patient didn't complete the study and is a major violator.
10107	The patient didn't complete the study and is a major violator.
10118	The patient didn't complete the study and is a major violator.
10122	The patient didn't complete the study and is a major violator.
10127	The patient didn't complete the study and is a major violator.
10401	The patient didn't complete the study and is a major violator.
10507	The patient didn't complete the study and is a major violator.
10508	The patient didn't complete the study and is a major violator.
10514	The patient didn't complete the study and is a major violator.
10517	The patient didn't complete the study and is a major violator.
105174	The patient didn't complete the study and is a major violator.
10519	The patient didn't complete the study and is a major violator.
105233	The patient didn't complete the study and is a major violator.
10542	The patient didn't complete the study and took non-permitted medications and is a major violator.
10704	The patient didn't complete the study and is a major violator.
10720	The patient didn't complete the study and is a major violator.
11303	The patient didn't complete the study and is a major violator.
11309	The patient didn't complete the study and is a major violator.
11313	The patient didn't complete the study and is a major violator.
11364	The patient didn't complete the study and is a major violator.
20511	The patient didn't complete the study and is a major violator.
20529	The patient didn't complete the study and is a major violator.
20903	The patient didn't complete the study and is a major violator.
21643	The patient didn't complete the study and is a major violator.
21705	The patient didn't complete the study and is a major violator.
10904	Poor medication compliance, major violation
21004	Poor medication compliance, major violation
21430	Poor medication compliance, major violation
21614	Medication compliance not computable, major violation
21624	Medication compliance not computable, major violation
21627	Medication compliance not computable, major violation

Since the efficacy of the study drugs is well-known to be achieved in the long period (15-30 days), the Sponsor considered as minor impact all study drug treatment discontinuations occurred on the day before or the day of each scheduled visit, which was a criterion for patients exclusion according to the PPS definition included the clinical study protocol; thus no patient will be excluded from PPS due to this type of (minor) deviation.

During the Data Review meeting the definition of the Per-protocol Set was also re-discussed and it was decided to modify it as follows:

The PPS includes all randomised patients of the FAS ***who have at least one post-baseline efficacy assessment at visit 5 or visit 6*** *and who fulfil the eligibility criteria of the Protocol, perform the Visit at the end of the Treatment Period, complete the treatment with a compliance of at least 80%, don't take prohibited drugs during the course of the Study and don't discontinue the Study Drug treatment during the day before or the day of each scheduled Visit.*

During the Data Review Meeting will be evaluated if a patient interrupting the Study because of uncontrolled hypertension (i.e. office SBP ≥ 179 mmHg or DBP ≥ 110 mmHg) will or not be included in the statistical analysis with his/her last blood pressure evaluation.

If, within each treatment group, in the subpopulations of Italian or Chinese patients respectively, a patient belongs to genetic profile 2 because a gene pair alone (i.e. gene pair not included as part of gene pairs combinations) which is not represented by any other within the same group, then ALL the patients carrying by such gene pair will be excluded from the PPS.

The reason for the change of PPS definition as above (potential exclusion of patients with particular gene pair belonging to genetic profile 2) is given by a purely clinical consideration: standing the complexity of these genotype-phenotype relationships, that are not fully understood yet, the sponsor highlighted the need to minimize the genetic heterogeneity across the treatment arms by maximizing the frequency similarity of the gene-pairs across the different arms.

In the majority of the patients, the inclusion in the genetic profile 2 was due to the presence of only 1 gene-pair; so, for the reasons given above, the Sponsor decided to exclude from the PPS those patients that were included in the profile 2 because of the presence of only one gene-pair if this will not be present in all the treatment arms which will be compared.

As a consequence of the modified PPS definition, all patients in listing 8.2 (appendix E, G) and reported here below will be excluded from PPS, because they don't have a post-baseline efficacy assessment at visit 5 or visit 6:

**Subject ID**	**Comment**
10107	Patient without any post-baseline efficacy evaluation at visit 5 or visit 6
10118	Patient without any post-baseline efficacy evaluation at visit 5 or visit 6
10122	Patient without any post-baseline efficacy evaluation at visit 5 or visit 6
10127	Patient without any post-baseline efficacy evaluation at visit 5 or visit 6
10401	Patient without any post-baseline efficacy evaluation at visit 5 or visit 6
10507	Patient without any post-baseline efficacy evaluation at visit 5 or visit 6
10508	Patient without any post-baseline efficacy evaluation at visit 5 or visit 6
10514	Patient without any post-baseline efficacy evaluation at visit 5 or visit 6
10542	Patient without any post-baseline efficacy evaluation at visit 5 or visit 6
10704	Patient without any post-baseline efficacy evaluation at visit 5 or visit 6
10720	Patient without any post-baseline efficacy evaluation at visit 5 or visit 6
20511	Patient without any post-baseline efficacy evaluation at visit 5 or visit 6
21643	Patient without any post-baseline efficacy evaluation at visit 5 or visit 6

21705	Patient without any post-baseline efficacy evaluation at visit 5 or visit 6

PP population: 247 subjects

Subjects excluded from the PP population: 32 subjects

Further exclusions of patients from PPS, due to the additional a-priori criteria on gene pairs for Profile 2 within treatment groups highlighted in the updated PPS definition here above, will be identified after unblinding.

6.1.6 Population Summary

Population	Number of Subjects
Screened subjects	902
Randomized subjects	279
Safety population	275
FAS population	274
PP population	247

7. Appendices

A. PST2238-DM-10-001_ListingsforStatisticalAnalysis_Draft1(DataReview)_20180403_1

B. PST2238-DM-10-001_ListingsforStatisticalAnalysis_Draft1(DataReview)_20180403_2

C. PST2238-DM-10-001_ListingsforStatisticalAnalysis_Draft2(DataReview)_20180417_1

D. PST2238-DM-10-001_ListingsforStatisticalAnalysis_Draft2(DataReview)_20180417_2

E. PST2238-DM-10-001_ListingsforStatisticalAnalysis_Final1 (DataReview)_20180420_1

F. PST2238-DM-10-001_ListingsforStatisticalAnalysis_Final1(DataReview)_20180420_2

G. PST2238-DM-10-001_ListingsforStatisticalAnalysis_Final2.0 (DataReview)_20180420_1

H. PST2238-DM-10-001_ListingsforStatisticalAnalysis_Final2.0(DataReview)_20180420_2

I. PEARL-HT_Italy_Protocol Violation_tracker_180328_GB

J. CVT-CV-001_ctms_Protocol deviation list_20180330 updated

K. PEARL-HT_Italy_Protocol Violation_tracker_180328_final

L. Abby protocol deviati Tai Rosta 18.4.10

M. Geneticinfoadd.xlsx
